# Enhancing the *β*‐Oxidation‐Like Pathway for the Optimal Production of the Immunosuppressant Mycophenolic Acid

**DOI:** 10.1002/advs.202508826

**Published:** 2025-08-11

**Authors:** Baoqiang Fan, Yinuo Liu, Lu‐ping Chi, Chaofan Yang, Shengying Li, Wei Zhang

**Affiliations:** ^1^ State Key Laboratory of Microbial Technology Shandong University No. 72 Binhai Road Qingdao Shandong 266237 China; ^2^ CAS and Shandong Province Key Laboratory of Experimental Marine Biology Institute of Oceanology Chinese Academy of Sciences Qingdao Shandong 266071 China; ^3^ Laboratory for Marine Biology and Biotechnology Qingdao Marine Science and Technology Center Qingdao Shandong 266237 China; ^4^ Shenzhen Research Institute of Shandong University Shenzhen 518057 China

**Keywords:** mycophenolic acid, biosynthesis, fungal natural product, metabolic engineering, *β*‐oxidation

## Abstract

The recruitment of catabolic *β*‐oxidation enzyme cascades and their reaction logic for natural product biosynthesis remains underexplored, representing a significant opportunity for synthetic biology to engineer novel pathways for structurally unique metabolites. In this study, the first functional reconstitution of the fungal *β*‐oxidative cascade responsible for assembling the immunosuppressant mycophenolic acid (MPA) is reported. Through in vitro enzyme assays, five peroxisomal enzymes are identified that cooperatively mediate two iterative rounds of side‐chain cleavage of the biosynthetic precursor MFDHMP‐3C and revealed a key oxidative strategy for pharmacophore formation of MPA. These enzymes catalyzed sequential oxidation, dehydrogenation, hydration, reduction, isomerization, and reverse Claisen condensation reactions, mirroring canonical *β*‐oxidation while adapting it for biosynthetic purposes. Furthermore, integrated overexpression of the rate‐limiting peroxisomal acyl‐CoA oxidase PbACOX323, peroxisomal biogenesis factor PbPex337, and endoplasmic reticulum (ER)‐localized oxygenase MpaB’ in *Penicillium brevicompactum* NRRL864 increased MPA production by 50% (from 0.77 to 1.15 g L^−1^), demonstrating the biotechnological efficacy of pathway optimization. This work establishes the first example of a full *β*‐oxidation‐like enzyme cascade in fungal natural product biosynthesis, providing a paradigm for the evolutionary repurposing of catabolic modules to drive synthetic innovation.

## Graphical abstract

The most elaborate known example of *β*‐oxidation‐like pathway recruitment in fungi is reported, involving a 10‐step oxidative chain‐shortening cascade catalyzed by five dedicated enzymes to generate the pharmacophore of mycophenolic acid (MPA)—a novel clinical immunosuppressant. Our findings illustrate how nature repurposes catabolic enzymes for complex biosynthesis, offering fundamental insights into biocatalysis and metabolic engineering for unnatural pathway design.

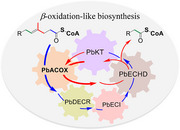

## 1 Introduction

**Figure 1:**
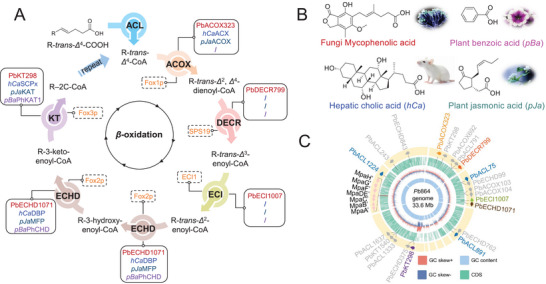
A), Peroxisomal *β*‐oxidative enzymes and pathway involved in natural product biosynthesis. Dash Boxes, Canonical *β*‐oxidation enzymes involved in unsaturated fatty acid degradation in *Saccharomyces cerevisiae* Fox1p, SPS19, ECI1, Fox2p, and Fox3p. Solid Boxes: *β*‐Oxidation enzymes reprogrammed for natural product biosynthesis. In the biosynthesis of fungal‐derived mycophenolic acid, a complete *β*‐oxidation‐like enzymatic cascade catalyzes two iterative rounds of side‐chain modification. This cascade includes: the acetyl‐CoA oxidase (PbACOX323), the 2,4‐dienoyl‐CoA reductase (PbDECR799), the *Δ* ^3^‐enoyl‐CoA isomerase (PbECI1007), the enoyl‐CoA hydratase/3‐hydroxyacetyl‐CoA dehydrogenase (PbECHD1071) and the 3‐ketoacyl‐CoA thiolase (PbKT298), which catalyze two *β*‐oxidation cycles. In contrast, only partial enzyme subsets are recruited in natural product biosynthetic systems: three enzymes, including ACOX, multifunctional protein (MFP), and KAT are found in hepatic cholic acid (*hCa*) biosynthesis; three enzymes of ACX/DBP/SCPx and two enzymes PhCHD/PhKAT1 are involved in the partial chain shortening in plant jasmonic acid (*pJa*) and benzoic acid (*pBa*) biosynthesis. The “/” indicates the absence of a corresponding enzyme. B), Structural formulas of mycophenolic acid, benzoic acid, jasmonic acid, and cholic acid. C), Genomic organization of peroxisomal *β*‐oxidation‐like enzymes and the *mpa’* BGC in *Pb*864.

Fatty acid synthases (FASs) are fundamental to fatty acid biosynthesis, utilizing an evolutionarily conserved modular architecture to iteratively extend carbon chains through coordinated catalytic steps.^[^ ^1^, ^2^ ^]^ This primary metabolic framework has been widely adopted as a blueprint for secondary metabolic systems.^[^ ^3^ ^]^ Polyketide synthases (PKSs) exemplify this evolutionary repurposing, leveraging the core FAS logic—thioester‐mediated chain elongation—to generate structurally diverse polyketides with pharmaceutical and industrial relevance.^[^ ^4^, ^5^ ^]^ In contrast, the *β*‐oxidation pathway, which catabolizes fatty acids through sequential carbon chain cleavage (**Figure** **1**A), has seen minimal adaptation in natural product biosynthesis. While partial *β*‐oxidation components are occasionally recruited, such as for chain shortening during plant jasmonate production^[^ ^6^ ^]^ or intermediate utilization in bacterial rhamnolipid assembly,^[^ ^7^ ^]^ no natural pathway fully integrates the complete *β*‐oxidation enzymatic cascade (Figure 1B, Figure S1, Supporting Information).

The fungal‐derived natural product mycophenolic acid (MPA, **1**), the active metabolite of immunosuppressants mycophenolate mofetil (e.g., Roche's CellCept) and mycophenolate sodium (e.g., Novartis’ Myfortic)^[^ ^8^ ^]^ first isolated and crystallized in 1893 as the first characterized antibiotic from nature, represents a landmark of natural product.^[^ ^9^ ^]^ Biosynthetically, the scaffold of **1** arises from the convergence of a polyketide synthase (PKS) and terpenoid biosynthetic systems.^[^ ^10^, ^11^ ^]^ Despite its historical significance, the genetic and enzymatic basis of the biosynthesis of **1** remained unresolved for over a century until the identification of four homologous biosynthetic gene clusters (BGCs) by us and other groups between 2011 and 2016^[^ ^12^, ^13^, ^14^, ^15^ ^]^ (Figure S2, Supporting Information). In 2019, our study elucidated the compartmentalized biosynthesis of **1** in *Penicillium brevicompactum* NRRL864 (*Pb*864), revealing an unprecedented cooperation between PKS machinery and peroxisomal *β*‐oxidation catabolism^[^ ^16^ ^]^ (Figure S3, Supporting Information). The peroxisomal activation of intermediate MFDHMP‐3C by acyl‐CoA ligase PbACL891 initiates a *β*‐oxidative chain‐shortening cascade essential for MPA maturation.^[^ ^16^ ^]^ However, the enzymatic drivers of this *β*‐oxidative cleavage step have remained uncharacterized, underscoring unresolved mechanistic complexities in this evolutionarily significant pathway.

This study represents the first systematic elucidation and characterization of a peroxisomal *β*‐oxidation‐like enzymatic cascade in fungal natural product biosynthesis, revealing two successive side‐chain shortening cycles mediated by iterative oxidation, dehydrogenation, hydration, reduction, isomerization, and reverse Claisen condensation reactions (Figure 1A). Specifically, we first identified two previously uncharacterized peroxisomal acyl‐CoA ligases (PbACL75 and PbACL1224) for maintaining the efficient metabolic flux through the *β*‐oxidation spiral. Further, we systematically characterized five peroxisomal enzymes from *Pb*864 genome —including acyl‐CoA oxidase ACOX323, enoyl‐CoA hydratase and 3‐hydroxyacyl‐CoA dehydrogenase ECHD1071, 3‐ketoacyl‐CoA thiolase KT298, 2,4‐dienoyl‐CoA reductase DECR799, and enoyl‐CoA isomerase ECI1007 – through integrated in vitro functional reconstitution and heterologous expression. These enzymes orchestrate the stepwise *β*‐oxidative processing of MFDHMP‐3C‐CoA (**2**‐CoA) and its demethylated derivative FDHMP‐3C‐CoA (**3**‐CoA), resolving a complete degradative biosynthesis of **1**. Furthermore, integrated overexpression of the rate‐limiting peroxisomal PbACOX323, peroxisomal biogenesis factor PbPex337, and endoplasmic reticulum (ER)‐localized oxygenase MpaB’ in *Pb*864 enhanced production of **1** from 0.77 to 1.15 g L^−1^, demonstrating the biotechnological potential of *β*‐oxidation pathway engineering to optimize natural product yields.

## 2 Results and Discussion

### 2.1 Genome Mining of Peroxisomal Acyl‐CoA Ligases

The exploration of the *β*‐oxidation pathway for **1** began with intermediate **2**, bearing a terminal carboxylic acid group. Since CoA acylation of **2** represents the first committed step in the *β*‐oxidation cycle, we initiated genome mining for acyl‐CoA ligases (ACLs) in *Pb*864. By utilizing the previously identified peroxisomal PbACL891 as a query for local gene Blast, 43 ACL‐encoding genes were identified, six of which encode a *C*‐terminal type I peroxisomal targeting signal (PTS1) (Figure 1C, Table S1, Supporting Information). Protein sequence alignment of these *Pb*864 ACLs with *Saccharomyces cerevisiae* FAA2 revealed that PbACL1637 exhibited a significantly truncated sequence compared to other homologs (Figure S4, Supporting Information). Phylogenetic analysis further classified them into distinct evolutionary clades, which indicated the divergent evolution of CoA‐ligases (Figure S5, Supporting Information). Transcriptome data demonstrated co‐expression of these peroxisomal ACLs with the *mpa’* BGC during production of **1** (Table S1, Figure S6, Supporting Information).

To verify their enzymatic activity, **2** or **3** was incubated with individual purified PbACLs in the presence of ATP, CoA, and Mg^2+^ (Figure S7, Supporting Information). HPLC and LC‐HRMS analyses identified three PbACLs (PbACL75, PbACL1224, PbACL891) capable of converting **2** and **3** to **2**‐CoA ([M+H]^+^, *obs*. 1138.2991, *calc*. 1138.3005) and **3**‐CoA ([M+H]^+^, *obs*. 1124.2827, *calc*. 1124.2849) (Figures S8 and S9, Supporting Information), respectively. Steady‐state kinetic assays using the malachite green phosphate method revealed that PbACL75 exhibited optimal catalytic efficiency toward both substrates, with comparable *k*_cat_/*K*_m_ values for **3** (175.61 mm ^−1^ min^−1^) and **2** (171.01 mm ^−1^min^−1^) (Figure S10, Table S2, Supporting Information).^[^ ^17^ ^]^ Further more, the incubation of PbACL75 toward compound **4**, which is the CoA hydroxylated intermediate derived right after one round of *β*‐oxidation was performed. HPLC and LC‐HRMS analyses identified PbACL75 capable of converting **4** to **4**‐CoA ([M+H]^+^, *obs*. 1110.2678, *calc*. 1110.2692; Figures S11, Supporting Information). The steady‐state kinetic assays revealed a higher *k*_cat_/*K*_m_ value for **4** (601.10 mm ^−1^ min^−1^, Figure S10G, Table S2, Supporting Information). These results suggest functional redundancy among peroxisomal PbACLs to ensure efficient *β*‐oxidation during **1** biosynthesis.

It is worth noting that the CoA ligation step is not rare in fungal secondary metabolism, as exemplified by phenylacetic acid CoA ligase (PCL) in penicillin G and isopenicillin N‐CoA ligase (CefD1) in cephalosporin C biosynthesis,^[^ ^18^, ^19^ ^]^ respectively. The homologs of PCL (PbACL791) and CefD1 (PbACL243) were identified in *Pb*864, however, neither demonstrated activity toward **2** and **3**, underscoring substrate specificity in ACL‐mediated *β*‐oxidation.

### 2.2 Identification of Peroxisomal Acyl‐CoA Oxidase

In principle, the second reaction of peroxisomal *β*‐oxidation should be classically catalyzed by an acyl‐CoA oxidase (ACOX), which mediates the *α*/*β* desaturation of acyl‐CoAs to produce the essential 2‐*trans*‐enoyl group (Figure 1A).^[^ ^20^ ^]^ Using the gene sequence of the ACOX Pox1p/Fox1p from *S. cerevisiae* S288C as a query,^[^ ^21^ ^]^ we identified a single peroxisome‐localized ACOX encoded by *PbACOX323* containing a PTS1 signal in the genome of *Pb*864 (Figure 1C). Sequence alignment indicated PbACOX323 as a dimeric FAD‐binding protein belonging to the acyl‐CoA dehydrogenase superfamily^[^ ^22^ ^]^ (Figures S12 and S13, Supporting Information).

**Figure 2:**
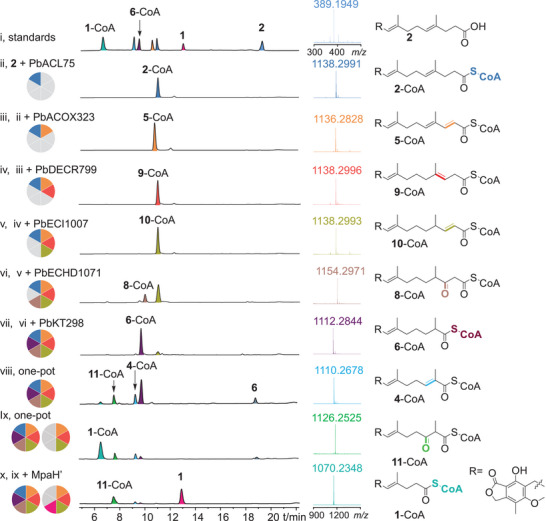
In vitro reconstitution of the *β*‐oxidation pathway for the biosynthesis of **1**. i) Mixed standards. ii) The CoA ligation reaction of **2** mediated by PbACL75 for 1 h. iii) The finished reaction (ii) followed by the addition of acyl‐CoA oxidase PbACOX323, converting **2**‐CoA to **5**‐CoA. iv) The finished reaction (iii) followed by the addition of peroxisomal 2,4‐*trans*‐dienoyl‐CoA reductase PbDECR799, leading to the production of **9**‐CoA. v) The finished reaction (iv) followed by the addition of enoyl‐CoA isomerase PbECI1007, isomerizing **9**‐CoA to **10**‐CoA. vi) The finished reaction (v) followed by the addition of hydratase‐dehydrogenase enzyme PbECHD1071, resulting in the installation of the 3‐keto group of **8**‐CoA. vii) The finished reaction (vi) followed by the addition of 3‐ketoacyl‐CoA thiolase PbKT298 leading to the detection of the two carbon‐shorter product **6**‐CoA together with acetyl‐CoA (see Figure S41, Supporting Information). The production of **6** might be resulted from spontaneous hydrolysis of **6**‐CoA. viii) One‐pot reaction including the 6 peroxisomal enzymes (5 µm each) and **2** for 3 h. ix) Further addition of PbACOX323, PbECHD1071, and PbKT298 (5 µm each) into the one‐pot reaction mixture of (viii) for another 3 h. x) Introduction of hydrolase MpaH’ into the reaction mixture (ix), resulting in the production of **1**.

Functional reconstitution showed that adding purified PbACOX323 to PbACL75‐mediated acylation reactions of **2** yielded a new product by HPLC and LC‐HRMS analysis (**Figure** **2**, trace iii; [M+H]^+^, *obs*. 1136.2828, *calc*. 1136.2849; Figures S14B,C, Supporting Information). The chemical structure of the new product was deduced as 2,4‐*trans*‐dienoyl‐MFDHMP‐3C‐CoA (**5**‐CoA) with the presence of a characteristic C2‐C3 double bond at 5.69 and 7.06 *ppm* in the ^1^H NMR spectrum (Figures S15 and S16, Table S3, Supporting Information). Additionally, the incubation of PbACOX323 and PbACL75 with MFDHMP‐5C (**6**, identified in our previous work^[^ ^16^ ^]^) produced MFDHMP‐5C‐CoA (**6**‐CoA; [M+H]^+^, *obs*. 1112.2844, *calc*. 1112.2849; Figure S17, trace ii, Figure S18B, Supporting Information) and 2‐*trans*‐MFDHMP‐5C‐CoA (**4**‐CoA; *obs*. 1110.2678, *calc*. 1110.2692; Figure S17, trace iii, Figure S18B, Supporting Information). The latter was further confirmed by direct acylation of **4** with PbACL75 (Figure S18, trace vi–vii, Supporting Information). Steady‐state kinetic assays revealed that PbACOX323 exhibited good catalytic efficiency toward **2**‐CoA, with a *k*_cat_/*K*_m_ value at 1484.78 mm ^−1^ min^−1^ (Figure S10H, Table S2, Supporting Information)

Interestingly, robing the *Pb*864 genome with four potential ACOX from *Penicillium chrysogenum* ^[^ ^23^ ^]^ identified three additional peroxisomal oxidases, including PbACOX103, PbACOX104, and PbACOX692 (Figures 1C, S12 and S13, Supporting Information). However, none of these three enzymes could catalyze the *α*/*β*‐desaturation of **2**‐CoA or **6**‐CoA (Figure S19, Supporting Information), indicating the functional specificity of PbACOX323 in this *β*‐oxidative cascade. Phylogenetic tree analysis indicates that PbACOX323 forms a separate branch from PbACOX692, PbACOX104, and PbACOX103 cluster with *Aspergillus* ACOXs and yeast Fox1p, suggesting functional specialization. It's interesting to find that fungi‐derived ACOXs form a distinct evolutionary clade separate from those of animals and plants (Figure S20, Supporting Information).

### 2.3 Functional Characterization of Peroxisomal 2,4‐Dienoyl‐CoA Reductase

Initially, we hypothesized that the subsequent metabolic steps would involve the hydration and dehydrogenation of **5**‐CoA at the C2═C3 double bond, reactions typically catalyzed by enoyl‐CoA hydratase and 3‐hydroxyacyl‐CoA dehydrogenase (ECHD, also known as multifunctional protein, MFP).^[^ ^22^ ^]^ Accordingly, genome mining using *S. cerevisiae* Fox2p^[^ ^24^ ^]^ as a query identified three putative ECHD homologs (PbECHD1071, PbECHD375, and PbECHD99). Unexpectedly, recombinant PbECHD1071 showed no activity toward **5**‐CoA in the presence of NAD^+^ and FAD,^[^ ^25^, ^26^ ^]^ suggesting this compound is not its physiological substrate (Figure S21A, Supporting Information). However, enzymatic assays with 3‐hydroxy‐MFDHMP‐3C‐CoA (**7**‐CoA, CoA acylation of 3‐hydroxy‐MFDHMP‐3C (**7**), which was identified in our previous work^[^ ^16^ ^]^) revealed successful conversion to 3‐keto‐MFDHMP‐3C‐CoA (**8**‐CoA, [M+H]^+^, *obs*. 1154.2971, *calc*. 1154.2954, Figures S21B–D, Supporting Information), as evidenced by HPLC and LC‐HRMS analysis. These results demonstrate that the recombinant PbECHD1071 was successfully purified in its catalytically active form, yet it does not function as the predicted downstream enzyme responsible for modifying **5**‐CoA.

Reexamination of the *β*‐oxidation pathway of unsaturated fatty acids indicates that the conversion of 2,4‐trans (*Δ* ^2^,*Δ*⁴) conjugated double bonds as seen in **5**‐CoA to a *Δ* ^3^ double bond is a prerequisite for subsequent oxidation, which is a transformation typically mediated by 2,4‐dienoyl‐CoA reductase (DECR) (Figure 1A).^[^ ^27^ ^]^ Consistent with this mechanistic requirement, genomic analysis using SPS19 from *S. cerevisiae S288C* as a query identified PbDECR799, a putative peroxisomal DECR bearing a canonical PTS1 motif in *Pb*864 (Figures 1C and S22, Supporting Information). Phylogenetic analysis reveals that PbDECR799 branches independently, indicating potential functional specialization possibly in mediating the biosynthesis of **1**. Furthermore, the results indicates that DECR is significantly separated in fungi, animals, and plants in terms of evolution. In the fungal evolutionary branch, DECR in *Penicillium*, *Aspergillus*, and *S. cerevisiae* is clearly differentiated (Figure S23, Supporting Information).

Further functional validation was performed by adding recombinant PbDECR799 to a reaction mixture containing PbACL75, PbACOX323, and substrate **2**, which yielded 3‐*trans*‐enoyl‐MFDHMP‐3C‐CoA (**9**‐CoA, [M+H]^+^, *obs*. 1138.2996, *calc*. 1138.3005) by HPLC and LC‐HRMS analysis (Figure 2, trace iv, Figure S24, Supporting Information). The chemical structure of **9** (alkaline hydrolysis product of **9‐**CoA) was further determined by the presence of the 3‐*trans* olefin signal at 5.20 *ppm* and the disappearance of the original *∆* ^2^,*∆* ^4^ conjugated system, which are replaced by two new methylene groups (2.96 *ppm* and 1.95 *ppm*) in the ^1^H NMR spectrum (Figures S25 and S26; Table S4, Supporting Information). The *k*_cat_/*K*_m_ value of PbDECR799 toward **5**‐CoA was determined as 362.49 mm ^−1^ min^−1^ by monitoring NADPH consumption (Figures S10I and Table S2, Supporting Information).^[^ ^28^ ^]^

### 2.4 Characterization of Peroxisomal Enoyl‐CoA Isomerase

The *β*‐oxidation process requires isomerization of the 3‐*trans* double bond to 2‐*trans* configuration via enoyl‐CoA isomerase (ECI) to facilitate the *β*‐oxidation process^[^ ^29^ ^]^ (Figure 1A). One single putative peroxisomal enzyme, PbECI1007 with a PTS1, was detected in the genome of *Pb*864 by using the encoding sequence of ECI1 from *S. cerevisiae* S288C as a probe^[^ ^29^ ^]^ (Figures 1C and S27, Supporting Information). Phylogenetic analysis of the ECI genes revealed a clear evolutionary divergence among fungal, animal, and plant lineages, indicating independent evolution of peroxisomal ECI in these eukaryotic kingdoms. Notably, PbECI1007 clusters within the *Penicillium* clade but displayed significant sequence divergence from both *Aspergillus* and yeast orthologs, suggesting potential functional specialization (Figure S28, Supporting Information).

To demonstrate its function, recombinant PbECI1007 was added to a reaction system containing PbACL75, PbACOX323, PbDECR799, and substrate **2**. Initial HPLC and LC‐HRMS analyses showed no detectable differences compared to the control (Figure 2, trace v, Figure S29B, Supporting Information), likely due to overlapping physicochemical properties (identical molecular weight and polarity) between the substrate **9**‐CoA and the expected product 2‐*trans*‐enoyl‐MFDHMP‐3C‐CoA (**10**‐CoA).

To prove this hypothesis, **9**‐CoA was purified and incubated with PbECI1007, followed by alkaline hydrolysis (0.1 m NaOH) to remove the CoA moiety. Chiral HPLC analysis of the hydrolyzed products (**9** vs **10**) revealed distinct retention times (Figure S30, Supporting Information), confirming isomerization. Further confirmation of the structure of **10** was accomplished by detecting the presence of C2 and C3 olefins at, respectively, 5.70 and 6.71 *ppm* in the ^1^H NMR spectrum (Figures S31 and S32, Table S5, Supporting Information). These results unequivocally demonstrate the role of PbECI1007 in isomerizing the 3‐*trans* double bond of **9**‐CoA to generate **10**‐CoA (Figure 2, trace v, Figures S29B,C, Supporting Information), completing a critical step in the peroxisomal *β*‐oxidative cascade.

### 2.5 Genome Mining and Characterization of Peroxisomal Hydratase‐Dehydrogenase Enzymes

After oxidative reduction and isomerization of 2,4‐trans (Δ^2^,Δ⁴) conjugated double bonds of **4**‐CoA, the recombinant PbECHD1071 was incubated with purified **10**‐CoA in the presence of NAD^+^ and FAD.^[^ ^25^, ^26^ ^]^ A new product with 16 atomic mass units more than that of the substrate was detected, which was revealed identical retention times and molecular weight with 3‐keto‐MFDHMP‐3C‐CoA (**8**‐CoA, [M+H]^+^, *obs*. 1154.2971, *calc*. 1154.2954), consistent with *β*‐diketone formation (Figure S33B, Supporting Information). However, the instability of *β*‐diketone group precluded further isolation and NMR analysis.^[^ ^30^ ^]^ The previously identified 3‐hydroxy‐MFDHMP‐3C (**7**) was demonstrated, which incubated with PbACL75 and PbECHD1071, yielding a product **8**‐CoA ([M+H]^+^, *obs*. 1154.2971, *calc*. 1154.2954; Figure S21B, Supporting Information). This supports a two‐step mechanism: the hydratase domain of PbECHD1071 converts **10**‐CoA to 3‐hydroxy‐MFDHMP‐3C‐CoA (**7**‐CoA), followed by dehydrogenase‐mediated oxidation to **8**‐CoA (Figure 2, trace vi). Parallel assays with substrate **6** in the presence of PbACL75, PbACOX323 and PbECHD1071, sequentially produced **4**‐CoA and 3‐keto‐MFDHMP‐5C‐CoA (**11**‐CoA, [M+H]^+^, *obs*. 1126.2625, *calc*. 1126.2641; Figures S18 and S34; **6**‐CoA→**4**‐CoA→**11**‐CoA) by LC‐HRMS detection. Functional characterization of recombinant PbECHD1071 revealed its dual catalytic activity. PbECHD1071 showed no reactivity with **9**‐CoA with the 3‐*trans* olefin group (Figure S35A, Supporting Information), confirming its strict substrate dependency on prior DECR and ECI processing. PbECHD1071 strictly requires FAD for activity, unlike its homologs Fox2p and petunia PhCHD,^[^ ^31^, ^32^ ^]^ suggesting divergent cofactor utilization potentially linked to structural stabilization—a feature more akin to bacterial FAD‐dependent hydratases. This functional divergence warrants further investigation of the FAD role in PbECHD1071 catalysis. The *k*_cat_/*K*_m_ value of PbECHD1071 toward **10**‐CoA was determined as 128.42 mm ^−1^ min^−1^ by steady‐state kinetic analysis (Figure S10J, Table S2, Supporting Information).

In the exception of PbECHD1071, genome mining using the Fox2p from *S. cerevisiae* ^[^ ^24^ ^]^ as the probe identified another two ECHD candidates (PbECHD375, PbECHD99). Additional homology‐based searches using ECHDs from *P. chrysogenum* ^[^ ^23^ ^]^ revealed two more isoforms (PbECHD843 and PbECHD762). Sequence alignment showed that PbECHD1071 retains both an *N*‐terminal dehydrogenase domain and a *C*‐terminal type II hydratase domain (48% protein identity to Fox2p), while the other four homologs lack the dehydrogenase domain (Figure 1C, Figures S36 and S37, Supporting Information).

Phylogenetic analysis of ECHD genes revealed a deep divergence between fungi, animals, and plants, supporting independent evolutionary trajectories of this enzyme family across eukaryotes. PbECHD1071 was grouped within a fungal‐specific clade alongside *Aspergillus* Fox2 but exhibited significant divergence from the *Saccharomyces cerevisiae* Fox2p. This distinct placement implies that PbECHD1071 may represent a lineage‐specific peroxisomal multifunctional enzyme in *P. brevicompactum*, potentially adapted for specialized metabolic demands (Figure S38, Supporting Information). On the other hand, the other four homologs (PbECHD762, PbECHD843, PbECHD375, and PbECHD99) formed a distinct clade, clustering with orthologs from animals and plants (Figure S38, Supporting Information). Functional validation of these homologs confirmed their lack of activity against **4**‐CoA and **10**‐CoA (Figure S35B, Supporting Information), reinforcing their divergence and suggesting potential substrate specificity.

### 2.6 Functional Reconstitution of Peroxisomal 3‐Ketoacyl‐CoA Thiolase

The final step of the *β*‐oxidation cycle is mediated by 3‐ketoacyl‐CoA thiolase (KT), which catalyzes the cleavage of *β*‐ketoacyl‐CoA substrates into a shortened acyl‐CoA derivative and acetyl‐CoA^[^ ^22^ ^]^ (Figure 1A). Using the encoding sequence of the peroxisomal thiolase Pot1p/Fox3p from *S. cerevisiae* ^[^ ^33^ ^]^ and three predicted thiolases from *P. chrysogenum* ^[^ ^23^ ^]^ as probes, three peroxisomal thiolases (PbKT298, PbKT339 and PbKT1540) harboring the *N*‐terminal type II peroxisomal targeting signal (PTS2) were mined from the *Pb*864 genome (Figures 1C and S39; Table S1, Supporting Information). Phylogenetic tree analysis indicates that KTs of fungi, animals, and plants diverged significantly. PbKT298 belongs to the core branch of thiolase in the *Penicillium* genus and is highly conserved with other species in the same genus. PbKT1540 and PbKT339 belong to another fungal branch, closely related to the *Trichoderma* genus, and may represent different thiolase subtypes (Figure S40, Supporting Information).

Functional screening revealed substrate specificity among these enzymes. When individually added to a reconstituted reaction system containing PbACL75, PbACOX323, PbDECR799, PbECI1007, and PbECHD1071 with **2** serving as substrate, only PbKT298 catalyzed chain shortening reaction to yield **6**‐CoA ([M+H]^+^, *obs*. 1112.2844, *calc*. 1112.2849) alongside acetyl‐CoA, as confirmed by HPLC and LC‐HRMS analysis (Figures 2, trace vii and S41, Supporting Information). Similarly, the incubation of PbKT298 with **6** in the presence of PbACL75, PbACOX323, and PbECHD1071 generated the final product **1**‐CoA by HPLC and LC‐HRMS, along with propionyl‐CoA (Figures S18 and S42B, Supporting Information). In contrast, neither PbKT339 nor PbKT1540 exhibited activity toward the diketone intermediate **8**‐CoA or **11**‐CoA (Figure S43, Supporting Information), underscoring the exclusive role of PbKT298 in this *β*‐oxidation‐like enzyme cascade.

### 2.7 “One‐pot” Reconstitution of *β*‐Oxidative Reactions In Vitro

After establishing each enzymatic steps involved in *β*‐oxidation‐like side chain cleavage, the one‐pot conversion of **2** to **1** was carried out in vitro. Particularly. when **2** was incubated with an equal concentration (5 µm) of the six cascade enzymes (PbACL75, PbACOX323, PbDECR799, PbECI1007, PbECHD1071, and PbKT298) and required cofactors for 3 h, resulted in near‐complete substrate consumption, yielding **6**‐CoA as the major product, alongside minor intermediates **4**‐CoA, **11**‐CoA, and trace **1**‐CoA (Figure 2, trace viii). This observation indicated rate‐limiting downstream processing (**6**‐CoA→**4**‐CoA→**11**‐CoA→**1**‐CoA), likely due to insufficient enzymatic activity. To address this bottleneck, after the first 3 h incubation, the concentration of PbACOX323, PbECHD1071, and PbKT298 was individually doubled, achieving progressive conversion of accumulated intermediates (Figure S44A, trace iii–v, Supporting Information). Subsequent supplementation with additional 5 µm aliquots of these three enzymes after the initial 3 h reaction markedly enhanced production of **1**‐CoA (Figure 2, trace ix). Further introduction of the pathway specific hydrolase MpaH’ quantitatively converted **1**‐CoA into the final product **1** (Figure 2, trace x), which is consistent to our previously identified results.^[^ ^16^ ^]^

To our delight, LC‐HRMS analysis of the one‐pot reaction identified the key intermediates of 3‐hydroxy‐MFDHMP‐3C‐CoA (**7**‐CoA, [M+H]^+^, *obs*. 1156.3161, *calc*. 1156.311; Figure S44B, trace iii, Supporting Information) and 3‐hydroxy‐MFDHMP‐5C‐CoA (**12**‐CoA, [M+H]^+^, *obs*. 1128.2769, *calc*. 1128.2798; Figure S44B, trace v, Supporting Information), further confirmed the function of PbECHD1071‐mediated hydroxylation.

Parallel functional reconstitution with the demethylated substrate **3** revealed analogous processing by the six‐enzyme cascade: **3** was sequentially converted to **3**‐CoA (PbACL75), 2,4‐*trans*‐dienoyl‐FDHMP‐3C‐CoA (**13**‐CoA; PbACOX323), 3‐*trans*‐enoyl‐FDHMP‐3C‐CoA (**14**‐CoA; PbDECR799), 2‐*trans*‐enoyl‐FDHMP‐3C‐CoA (**15**‐CoA; PbECI1007), 3‐hydroxy‐FDHMP‐3C‐CoA (**16**‐CoA) with 3‐keto‐FDHMP‐3C‐CoA (**17**‐CoA; PbECHD1071), and ultimately FDHMP‐5C‐CoA (**18**‐CoA) with acetyl‐CoA as a byproduct (PbKT298) based on HPLC and LCMS analysis (Figures S45–S47, Supporting Information).

Further validation using the carbon‐shortened intermediate **18** as a substrate for PbACL75, PbACOX323, PbECHD1071, and PbKT298 demonstrated successive formation of **18**‐CoA, 2‐*trans*‐FDHMP‐5C‐CoA (**19**‐CoA), 3‐hydroxy‐FDHMP‐5C‐CoA (**20**‐CoA) with 3‐keto‐FDHMP‐5C‐CoA (**21**‐CoA), and demethylmycophenolic acid‐CoA (**22**‐CoA) (Figures S48 and S49, Supporting Information). These results collectively confirm the evolutionary specialization of the six‐enzyme cascade for efficient *β*‐oxidative processing to the final product **1**‐CoA, highlighting its strict substrate selectivity and catalytic coordination.

### 2.8 Subcellular Localization of the Peroxisomal *β*‐Oxidation Enzymes

To demonstrate the peroxisomal localization of the six enzymes involved in the biosynthesis of **1**, we performed fluorescent protein tagging coupled with confocal laser scanning microscopy (CLSM).^[^ ^16^ ^]^ For example, to determine the subcellular location of PbACOX323, a green fluorescent protein (GFP) tag was fused to the *N*‐terminal of PbACOX323 (pTAex3‐*gfp*‐*PbACOX323*), and co‐expressed with the recombinant peroxisomal‐specific red fluorescent protein (RFP) reporter tagged with the *C*‐terminal SKL tripeptide (pTAex3‐*rfp^SKL^*) in the protoplasts of the *Aspergillus oryzae* M‐2‐3 strain (Figure S50, Tables S6 and S7, Supporting Information).

**Figure 3:**
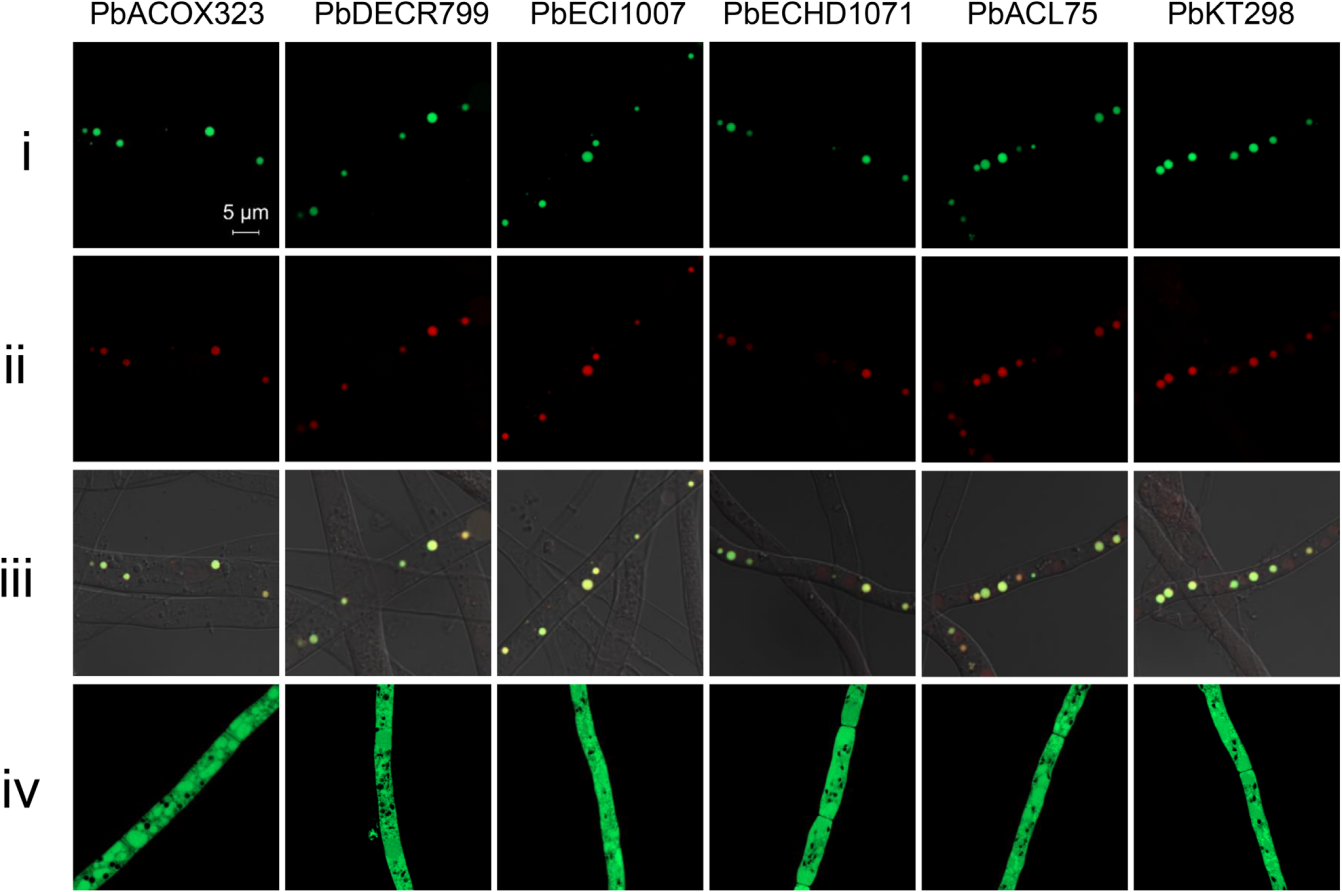
High‐resolution confocal images for subcellular localization of peroxisomal enzymes involved in the side chain cleavage of **1** (Magnification, 5  µm). i) A GFP tag was fused to the *N*‐terminus or *C*‐terminus of the peroxisomal enzymes (GFP‐PE). ii) Expression of the recombinant peroxisome‐specific RFP^SKL^ reporter. iii) Merged images of (i) and (ii) in bright field. iv) GFP‐PE fusion proteins with truncated PTS signal sequences.

CLSM observations revealed that the green fluorescence signals of GFP‐PbACOX323 were concentrated in small, dot‐like structures, which co‐localized with the peroxisomal RFP^SKL^ reporter (**Figure** **3**, PbACOX323‐i‐iii). When the PTS1‐type SKL‐tripeptide of GFP‐PbACOX323 was truncated, the distribution of GFP signals changed to a faint, diffuse pattern in the cytosol (Figure 3, PbACOX323‐iv). Thus, these results confirmed the PTS1‐dependent peroxisomal targeting of PbACOX323. Parallel analyses of PbACL75, PbECHD1071, PbDECR799, and PbECI1007 with the intact PTS1 sequences demonstrated the peroxisomal localization through an identical experimental strategy (Figure 3).

For PbKT298, which has an *N*‐terminal PTS2, the GFP tag was fused to its *C*‐terminus to generate the fusion protein PbKT298‐GFP. CLSM observations showed co‐localization of the green fluorescence signals of PbKT298‐GFP and the red fluorescence signals of the peroxisomal RFP^SKL^ reporter (Figure 3, PbKT298‐i‐iii). In contrast, the absence of the *N*‐terminal PTS2 resulted in the cytosolic diffusion of GFP signals (Figure 3, PbKT298‐iv). Taken together, we confirmed that PbACL75, PbACOX323, PbDECR799, PbECI1007, PbECHD1071, and PbKT298 are indeed peroxisomal proteins.

### 2.9 Metabolic Engineering for Enhanced Production of **1**

Having characterized the peroxisomal *β*‐oxidation‐like enzymatic steps involved in the side chain tailoring of **1**, we sought to increase its production. Considering that the ACOX‐catalyzed desaturation of acyl‐CoAs to form the 2‐*trans*‐enoyl moiety typically serves as the key rate‐limiting step during *β*‐oxidation,^[^ ^21^, ^22^ ^]^ we aimed to enhance the expression level of PbACOX323 by exchanging its native promoter with a stronger one. Specifically, we screened the promoters PgpdA (promoter of the 3‐phosphoglyceraldehyde dehydrogenase gene *gpdA* from *Aspergillus nidulans*),^[^ ^34^ ^]^ PtrpC (promoter of the tryptophan synthesis gene *trpC* from *A. nidulans*),^[^ ^35^ ^]^ and Ppgk (promoter of 3‐phosphoglycerate kinase gene *pgk* from *A. nidulans*).^[^ ^36^ ^]^ As expected, the yield of **1** in the *Pb*ACOX323^PgpdA^ mutant was succussed raising to 0.84 from 0.77 g L^−1^ of *Pb*864^WT^. However, no significant effects were observed in the *Pb*ACOX323^PtrpC^ and *Pb*ACOX323^Ppgk^ mutants (Figure S51A, Supporting Information). The evaluation of other identified *β*‐oxidation enzymes including PbDECR799, PbECI1007, PbECHD1071 and PbKT298 were overexpressed individually by promoter swapping. While each improved the production yield of **1** (0.79–0.83 g L^−1^ vs *Pb*864^WT^, Figures S51B–F, Supporting Information), none matched the significant boost from PbACOX323, confirming its role as the key pathway bottleneck. Future work will explore synergistic effects of multi‐enzyme engineering to further increase the yield of **1**.

**Figure 4:**
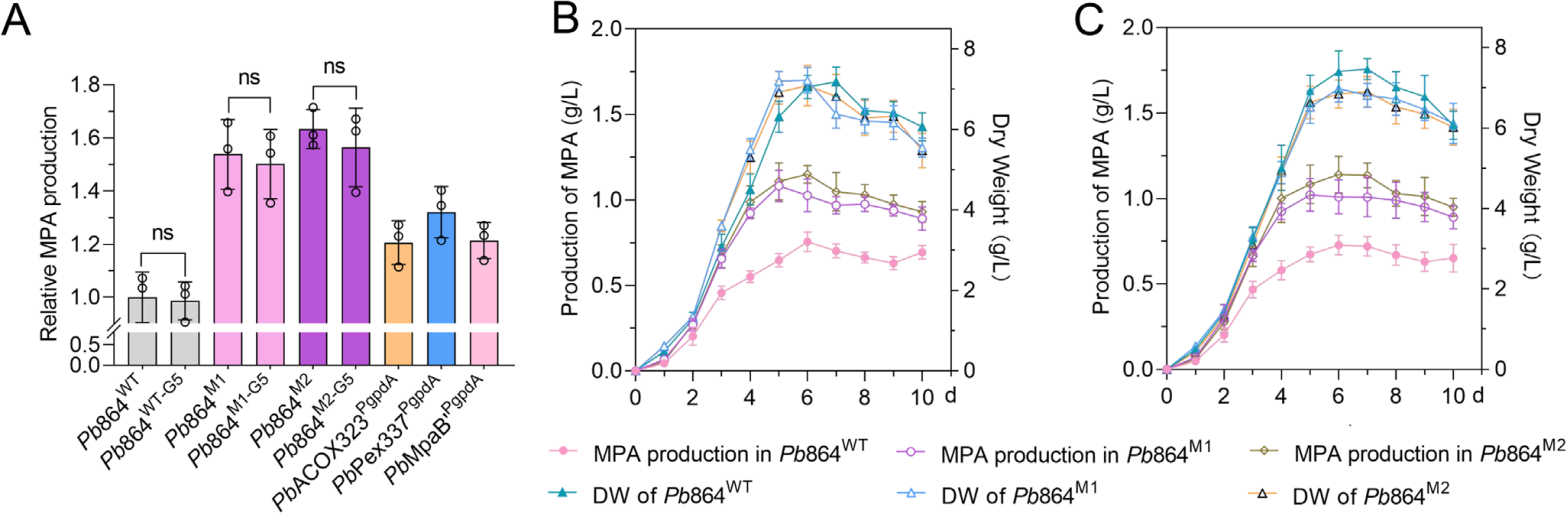
Metabolic engineering to increase the production of **1** in *Pb*864. A), Relative production of **1** by *Pb*864^WT^ and mutants *Pb*ACOX323^PgpdA^, *Pb*Pex337^PgpdA^, *Pb*MpaB’^PgpdA^, *Pb*864^M1^, *Pb*864^WT‐G5^, *Pb*864^M1‐G5^, and *Pb*864^M2‐G5^ and *Pb*864^M2^ by 6 days of fermentation. B), Dry cell weight (DW) and the production of **1** by *Pb*864^WT^, *Pb*864^M1^, and *Pb*864^M2^ during 10 days of fermentation. C), Dry cell weight (DW) and the production of **1** by *Pb*864^WT‐G5^, *Pb*864^M1‐G5^, and *Pb*864^M2‐G5^ during 10 days of fermentation. The production of **1** was calculated by plotting the integrated peak area to the standard curve; chloramphenicol was used as an internal standard. Data are shown as the mean ± SD (n = 3) of triplicate experiments (ns, *p* > 0.05).

We next targeted peroxisome proliferation by overexpression of the peroxisome biogenesis factor PbPex337, a homolog (26% protein identity) of *S. cerevisiae* peroxisome biogenesis factor Pex11p.^[^ ^37^ ^]^ Notably, the yield of **1** was increased to 0.93 g L^−1^ in the *Pb*Pex337^PgpdA^ mutant (**Figure** **4**A,B), suggesting a possible positive correlation between the number of peroxisomes and the production of **1**.

Next, we focused on increasing the supply of **2**, a key intermediate that precedes the entry into peroxisomal *β*‐oxidation and that is produced by the ER‐bound oxygenase MpaB’.^[^ ^16^ ^]^ Upon swapping the natural promoter of MpaB’ with PgpdA, the yield of **1** in the *Pb*MpaB’^PgpdA^ mutant reached 0.85 g L^−1^. However, spatially re‐localization of MpaB’ from the ER to peroxisomal membranes failed to enhance production (Figure S51A, Supporting Information), likely due to the disrupted peroxisomal enzyme import, mismatched biochemical environments, and metabolic pathway imbalance.

Finally, we constructed two mutants by integrating the overexpression constructs *Pb*ACOX323^PgpdA^ and *Pb*Pex337^PgpdA^ to generate *Pb*864^M1^; and constructs *Pb*ACOX323^PgpdA^, *Pb*Pex337^PgpdA^, and *Pb*MpaB’^PgpdA^ to generate *Pb*864^M2^. Monitoring of the biomass and production of **1** showed parallel growth for *Pb*864^M1^, *Pb*864^M2^, and *Pb*864^WT^ during 10 d of fermentation in 250 mL shake flasks. To our delight, the yield of **1** was indeed increased in both mutants, specifically, the maximum yield of **1** was 1.08 g L^−1^ at 5 d for *Pb*864^M1^ and 1.15 g L^−1^ at 6 d for *Pb*864^M2^ (Figure 4B, Table S8, Supporting Information). After five sequential passages on PDA medium, *Pb*864^M1‐G5^ and *Pb*864^M2‐G5^ maintained consistent production of **1**, with yields comparable to their first‐generation counterparts (Figure 4A,C). This stability confirms that the genetic modifications in these mutants remain intact without observable degradation in productivity.

## 3 Conclusion

The recruitment of *β*‐oxidation machinery for natural product biosynthesis exemplifies a remarkable evolutionary innovation in nature.^[^ ^38^, ^39^, ^40^ ^]^ Although *β*‐oxidation is traditionally recognized as a catabolic pathway central to energy homeostasis, its repurposing for natural product biosynthesis remains extraordinarily rare. Previous studies have only documented fragmented *β*‐oxidation components involved in specialized metabolic processes, such as partial chain shortening in plant jasmonic acid^[^ ^6^ ^]^ and benzoic acid biosynthesis,^[^ ^31^, ^32^ ^]^ C24 bile acid maturation in hepatic systems,^[^ ^41^ ^]^ or intermediate channeling in bacterial surfactants^[^ ^7^ ^]^ (Figure 1A,B). Nevertheless, the full reprogramming of this enzymatic cascade for construction of secondary metabolites had remained unprecedented.

**Figure 5:**
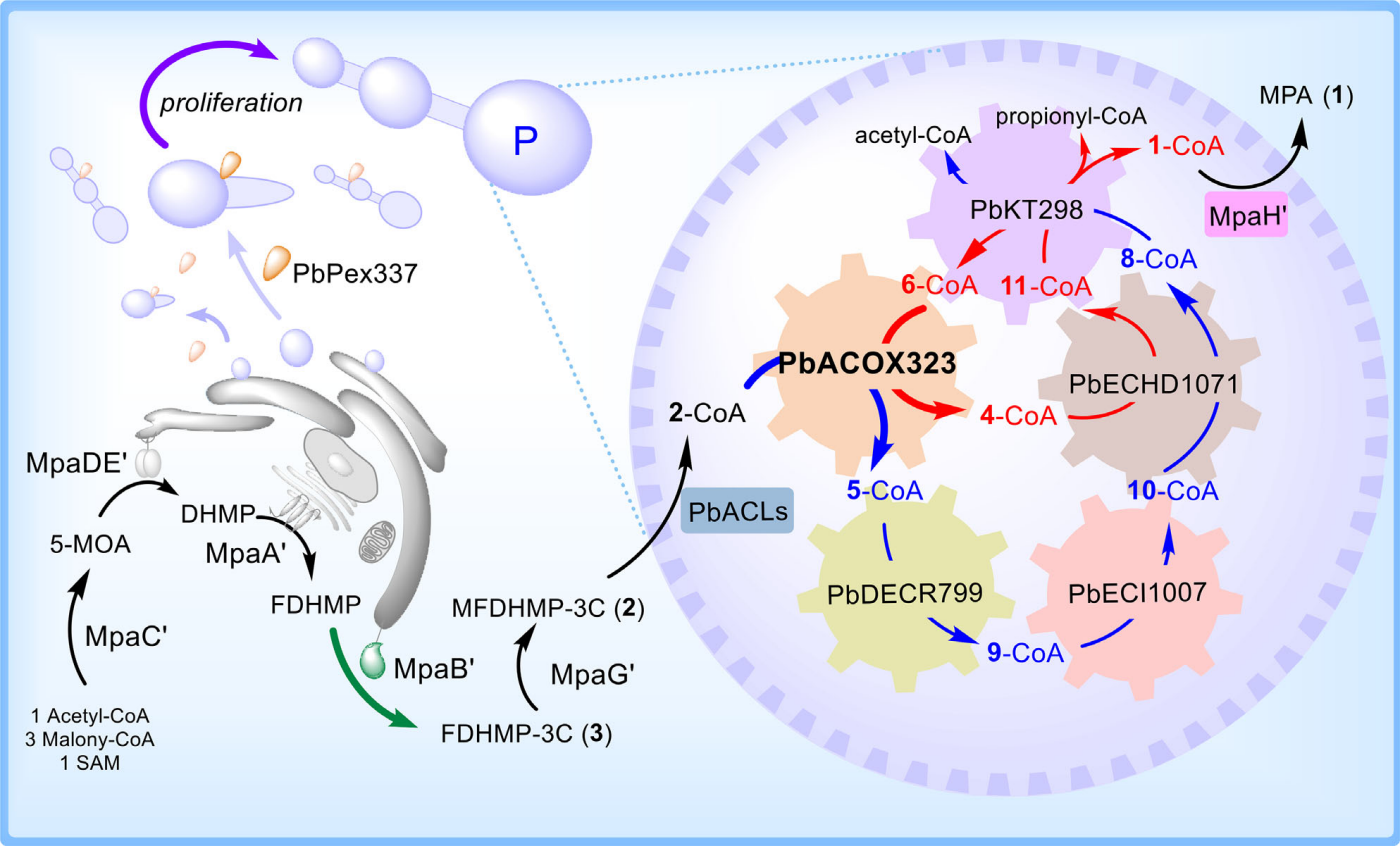
The compartmentalized biosynthesis of **1** and the *β*‐oxidation‐like pathway engineering for enhancing the production of **1** in *Pb*864. The purple arrow: overexpression of PbPex337; the bold dark green arrow: overexpression of MpaB’; the bold blue and bold red arrows: overexpression of PbACOX323. The red arrow: the metabolic pathway for *∆* ^2^ double bond intermediate; the blue arrow: the metabolic pathway for intermediate with *∆* ^2^,*∆* ^4^ conjugated double bonds system.

Here, we present the first functional reconstitution of a *β*‐oxidation‐inspired enzymatic cascade dedicated to the biosynthesis of the immunosuppressant drug—MPA. Three phylogenetically distinct acyl‐CoA ligases (PbACL75, PbACL1224, and PbACL891) are further identified as critical flux regulators to establish substrate activation efficiency. Through comprehensive in vitro and in vivo analyses, we resolved a peroxisomal *β*‐oxidation‐like pathway wherein five core enzymatic components—PbACOX323, PbDECR799, PbECI1007, PbECHD1071, and PbKT298—mechanistically coordinate an iterative chain‐shortening mechanism for product maturation (Figures 1A and **5**).

Confocal microscopy confirmed the peroxisomal localization of the identified enzymes (Figure 3), complementing the previously established spatial organization of the biosynthesis of **1**, wherein cytosolic polyketide assembly, ER‐bound oxygenation, and Golgi‐localized prenylation converge with peroxisomal chain tailoring.^[^ ^16^ ^]^ This subcellular partitioning likely prevents cytotoxic intermediate accumulation^[^ ^42^ ^]^ while facilitating efficient substrate channeling—an evolutionary solution to balance metabolic efficiency and cellular fitness.^[^ ^43^, ^44^ ^]^ The translational potential of this mechanistic insight was further realized through rational engineering. By enhancing the rate‐limiting *β*‐oxidation enzymes (PbACOX323), the precursor‐supplying oxygenase (MpaB’), and the peroxisome proliferation via PbPex337 overexpression, a 49% yield improvement (0.77 → 1.15 g L^−1^) in the production of **1** was achieved (Figures 4 and **5**).

This study marks a paradigm shift in metabolic engineering by revealing nature's extraordinary capacity to reprogram the canonical catabolic *β*‐oxidation pathway for the biosynthesis of secondary products. By integrating enzymology with bioprocessing principles, we provide new evolutionary insights into nature's metabolic repurposing strategies while delivering actionable engineering blueprints for optimizing subcellularly organized biosynthesis systems. These advances herald a new way of pharmaceutical innovation through precise spatial control of metabolic pathways.

## Conflict of Interest

The authors declare no conflict of interest.

## Supporting information



Supporting Information

## Data Availability

The data that support the findings of this study are available in the supplementary material of this article.
