# Supplementary material for: Enhancing the β‐Oxidation‐Like Pathway for the Optimal Production of the Immunosuppressant Mycophenolic Acid
Source: Adv Sci (Weinh). 2025 Aug 11;12(41):e08826. doi: 10.1002/advs.202508826 (PMC12591190; doi:10.1002/advs.202508826)
Supplement: Supplementary file 1 — Supporting Information [file ADVS-12-e08826-s001.docx]

Supporting Information

Enhancing the *β*-Oxidation-like Pathway for the Optimal Production of the Immunosuppressant Mycophenolic Acid

*Baoqiang Fan, Yinuo Liu, Lu-ping Chi, Chaofan Yang, Shengying Li^*^ and Wei Zhang^*^*

**Experimental Section**

*Materials and Methods*: Unless otherwise specified, all chemicals and reagents used in this study were purchased from Sigma-Aldrich (MO, USA). Fast-digest restriction endonucleases used for cloning experiments were purchased from Thermo Scientific (PA, USA). Kits for plasmid mini-preparations and DNA gel extractions were acquired from Omega Bio-tek, Inc. (GA, USA). ClonExpress Ultra One Step Cloning Kit was purchased from Vazyme (Nanjing, China). The FlexiRun^TM^ premixed gel solution for SDS-PAGE, GelRed, Luria-Bertani broth (LB), and Terrific broth were obtained from MDBio. (Qingdao, China). Otherwise, growth medium components were bought from Hope Bio-Technology Co., Ltd. (Qingdao, China). All proteins were purified using Ni-NTA Sefinose^TM^ Resin (Settled Resin) from Sangon Biotech. Oligonucleotide primers and DNA sequencing service was obtained from Sangon Biotech (Shanghai, China). Synthetic genes were ordered from Beijing Genomics Institution (Shenzhen, China). Organic solvents for compound isolation and purification were bought from SINOPHARM (Shanghai, China). PCR reactions were carried out with 2 × Phanta Flash Master (Vazyme, Nanjing) or 2 × TsingKe Master Mix (TsingKe Biotech, Beijing).

*General Experimental Procedures*: HPLC analysis was performed using a YMC-Triart C18 (250 × 4.6 mm, 5 μm) column with a biphasic solvent system of acetonitrile (ACN) and water (containing 10 mM ammonium acetate) on an Agilent 1260 HPLC system, and the data was analyzed using Agilent OpenLab (version 3.2.0). The HPLC forward system employed a chiral column of CHIRALPAK^®^ IB N-3 (250 × 4.6 mm, 3 μm) with *n*-hexane and ethanol (93% : 7%) as the biphasic solvent. HPLC-HR-ESI-MS analyses were carried out on a Bruker impact HD High Resolution Q-TOF mass spectrometer. Data analysis of the MS results was performed using Bruker DataAnalysis (version 4.2). NMR spectra were acquired on a Bruker AVANCE III 600 spectrometer and processed using MestReNova software (version 14.1). Gene/protein sequence analysis was performed using NCBI blast + Mega-X + ESPript (version 3.0).

*Strains and Culture Conditions*: The *P. brevicompactum* NRRL 864 (*Pb*864) and mutants were cultivated in Potato Dextrose Broth (PDB), Potato Dextrose Agar (PDA) or M-PDA (PDA with 1 M sorbitol) medium. The *Aspergillus oryzae* M-2-3 was cultivated in CMP medium. *Escherichia coli* DH5*α* strain for vector construction and plasmid preparation was cultivated in LB or LBA (LB with 15% Agar) media. *E. coli* BL21(DE3) (for expression of *N*-His6-tagged protein) strains for protein expression was cultured in Terrific Broth media.

*Construction of pET28b-PbACLs for Protein Expression in E. coli*: The vector pET28b-PbACLs for expression of the *N*-His_6_-tagged CoA ligase PbACLs and other *β*-oxidation enzymes of *Pb*864 were synthetized with codon optimization by Wuxi Qinglan Biotech. Inc. (Wuxi, China). The vectors were confirmed by restriction digestion and DNA sequencing (Sangon Biotech Shanghai, China).

*Protein Expression and Purification*: A single colony of a certain *E. coli* BL21(DE3) transformant that carries a specific expression vector was inoculated into 5 mL LB medium containing 50 mg/L kanamycin and shaking incubated at 37°C overnight. The resultant seed culture (5 mL) was used to inoculate 500 mL LB medium containing 50 mg /L kanamycin. The cells were grown at 37°C for 2-3 h until OD_600_ reached 0.4-0.6. Then, isopropyl-D-thiogalactopyranoside (IPTG) was added to a final concentration of 0.2 mM to induce gene expression at 16°C, 150 rpm for 18 h. The culture was centrifuged at 6,000 *×g*, 4°C for 10 min at 4°C to collect cells.

The freeze-thaw cell pellets were resuspended with 50 mL lysis buffer (50 mM NaH_2_PO_4_, 300 mM NaCl, 10 mM imidazole, 10% glycerol, pH 8.0) and applied to sonication. Cell debris was removed by centrifugation at 10,000 *×g*, 4°C for 60 min. The supernatant was mixed with 1 mL Ni-NTA agarose (Qiagen, Venlo, Netherlands) for 1 h at 4°C. The slurry was loaded onto an empty column, and the column was washed stepwise with 100 mL lysis buffer and 400-600 mL wash buffer (50 mM NaH_2_PO_4_, 300 mM NaCl, 20 mM imidazole, 10% glycerol, pH 8.0). The bound His6-tagged proteins were eluted with 5-10 mL elution buffer (50 mM NaH_2_PO_4_, 300 mM NaCl, 250 mM imidazole, 10% glycerol, pH 8.0).

The proteins were further purified and concentrated with 30 kDa size-exclusion filters (Amicon, Millipore Corporation, Bedford, MA, US). The final desalting step was attained by buffer exchange into storage buffer (50 mM NaH_2_PO_4_, 150 mM NaCl, 10% glycerol, pH 7.4) with a PD-10 column (GE Healthcare, Illinois, US). It's worth noting that 2 mM ATP was added into all buffers for PbACLs purification.

*In-frame Promoter Replacement in P. brevicompactum NRRL 864*: The split-marker recombination strategy was employed to insert the promoters of PgpdA, PtrpC and Ppgk. Briefly, the plasmid pET28b-*hygB* was first constructed by inserting the hygromycin B resistance gene into the pET28b backbone between *Xho*I and *Hind*III restriction sites by ligation. Two DNA fragments corresponding to ~1.4 kb and ~1.3 kb of the 5’ and 3’ flanking sequences of PbACOX323 native promoter were amplified from *Pb*864 genomic DNA and promoter sequences inserted into the *Sal*I/*Hind*III and *Kpn*I/*Xho*I sites of pET28b-*HygR* vector sequentially, giving rise to pET28b-*PbACOX323HR-hygB-promoter*-*PbACOX323HL*.

Next, two DNA fragments were amplified from pET28b-*PbACOX323HR-HygR-promoter-PbACOX323HL* using the primers 323HL-FP/323HL-RP and 323HR-FP/323HR-RP. The PCR products were purified using the Cycle-pure Kit (Omega Bio-tek, Inc., Georgia, US), and then used to transform the protoplasts of *Pb*864 as previously described^[1]^. Transformants were selected on minimal medium supplemented with 1 M sorbitol, 2% glucose, and 200 μg mL^-1^ hygromycin.

*In vitro Enzymatic Reconstitution*: Unless otherwise specified, all enzymatic assays were carried out in a total volume of 100 μL reaction buffer (50 mM NaH_2_PO_4_, 150 mM NaCl, 10% glycerol, pH 7.4) at 30°C, and the boiled enzymes were used for negative control reactions. Following the reported protocol^[1]^, the *in vitro* assay of PbACLs, PbACL75 as an example, containing 1 μM PbACLs, 0.1 mM (M)FDHMP-3C (**2**/**3**), 2 mM ATP, 2 mM CoA, 10 mM MgCl_2_, 1 mM dithiothreitol (DTT), and 0.05% Triton X-100 in 100 μL reaction buffer was performed at 30°C for 1 h. The *in vitro* assay of PbACOX323, 1 μM PbACOX323 enzyme was added to the PbACL75 reaction mixture. *In vitro* assay of PbDECR799, 1 μM PbDECR799 and **2** mM NADPH were added into the reaction mixture of PbACOX323. For the *in vitro* assay of PbECI1007 enzyme, 1 μM PbECI1007 was added into the reaction mixture of PbDECR799. In vitro assay of PbECHD1071, 1 μM PbECHD1071, 2 mM FAD and 5 mM NAD^+^ were added into the reaction mixture of PbECI1007. For the *in vitro* assay of PbKT298 enzyme, 1 μM PbKT298 and 2 mM CoA was added into the reaction mixture of PbECHD1071.

The one-pot reaction for *in vitro* reconstitution of the pathway from MFDHMP-3C to **1** was performed for 3 h after mixing 0.2 mM of **2**, an equal concentration (5 μM) of PbACL75, PbACOX323, PbDECR799, PbECI1007, PbECHD1071, PbKT298 and MpaH’ in the presence of co-factors. The *in vitro* enzymatic reaction was quenched by adding a one-fold volume of methanol, vortexed for 10 min, and centrifuged at 14,000 *× g* for 10 min. Subsequently, the organic extract was directly applied for LC-HRMS analysis.

*Confocal Laser Scanning Microscopy (CLSM)*: The transformant bearing GFP or RFP fusion constructs was grown for 3-5 d at 28°C in CMP medium (Czapek-Dox supplemented with 3% maltose for induction and 1% peptone, 100 mL) to induce protein expression with the *α*-amylase promoter in a 250 mL Erlenmeyer flask, respectively. The fresh mycelia were transferred to slid after washing with sterilized water for three times. Confocal laser scanning fluorescence images of fungal structures were acquired using Zeiss LSM 900 confocal microscope equipped with Airyscan detectors. A krypton-argon laser was used as the source of excitation at 488 nm. The green fluorescence signals, the red fluorescence signals, and the bright field signals were recorded at 505 nm, 559 nm and 609 nm, respectively. The images were processed with ZEN 3.8 software.

*Construction of Aspergillus oryzae M-2-3 Expression Vectors*: The pTAex3 plasmid was used as the heterologous expression vector in the arginine auxotrophic mutant *Aspergillus oryzae* M-2-3 (*A*o_M-2-3_)^[1]^. The *PbACL75*, *PbACOX323*, *PbDECR799*, *PbECI1007*, *PbECHD1071*, *PbKT298* genes were amplified from cDNA of *Pb*864, using the corresponding primers (Table S7) as follows: *PbACL75*: pTAex3-GFP-PbACL75-*Nde*I-FP and pTAex3-GFP-PbACL75-*Nde*I-RP; *PbACOX323*: pTAex3-GFP-PbACO323-*Nde*I-FP and pTAex3-GFP-PbACO323-*Nde*I-RP; *PbDECR799*: pTAex3-GFP-PbDECR799-*Nde*I-FP and pTAex3-GFP-PbDECR799-*Nde*I-RP; *PbECI1007*: pTAex3-GFP-PbECI1007-*Nde*I-FP and pTAex3-GFP-PbECI1007-*Nde*I-RP; *PbECHD1071*: pTAex3-GFP-PbECHD1071-*Nde*I-FP and pTAex3-GFP-PbECHD1071-*Nde*I-RP; *PbKT298:* pTAex3-PbKT298-GFP-*Kpn*I-FP and pTAex3-PbKT298-GFP-*Kpn*I-RP. The PCR fragments of *PbACL75*, *PbACOX323*, *PbDECR799*, *PbECI1007*, *PbECHD1071* were individually cloned into the linearized pTAex3-GFP by *Nde*I single digestion with ClonExpress II One Step Cloning Kit (Vazyme Biotech, Nanjing, China) to generate pTAex3-*gfp*-*PbACL75*, pTAex3-*gfp*-*PbACOX323*, pTAex3-*gfp*-*PbDECR799*, pTAex3-*gfp*-*PbECI1007*, pTAex3-*gfp*-*PbECHD1071* respectively. For the PbKT298 with the *N*-terminal peroxisomal targeting signals (PTS2), the GFP tag was fused to its *C*-termini to give the fusion protein PbKT298-GFP. The PCR fragments of *PbKT298* was cloned into the linearized pTAex3-GFP by *Kpn*I single digestion with ClonExpress II One Step Cloning Kit to generate pTAex3-*PbKT298*-*gfp*. These vectors were constructed for expression of *N*-GFP-PbACL75-C, *N*-GFP-PbACO323-C, *N*-GFP-PbDECR799-C, *N*-GFP-PbECI1007-C, *N*-GFP-PbECHD1071-C and *N*-PbKT298-GFP-C, respectively. The pTAex3-*rfp^SKL^* vector was constructed before^[1]^.

To investigate the function of the PTS1-like signal at the *C*-terminus of PbACL75, PbACOX323, PbDECR799, PbECI1007, PbECHD1071, or PTS2-like signal at the *N*-terminus of PbKT298. The PCR fragments of *PbACL75*^Δ^*^PKL^, PbACOX323*^Δ^*^SKL^, PbDECR799*^Δ^*^SKL^, PbECI1007*^Δ^*^SHL^, PbECHD1071*^Δ^*^AKL^, PbKT298*^ΔP^*^TS2^* were individually cloned into the linearized pTAex3-GFP with ClonExpress II One Step Cloning Kit to generate the vectors pTAex3-*gfp*-*PbACL75*^Δ^*^PKL^*, pTAex3-*gfp*-*PbACOX323*^Δ^*^SKL^*, pTAex3-*gfp*-*PbDECR799*^Δ^*^SKL^*, pTAex3-*gfp*-*PbECI1007*^Δ^*^SHL^*, pTAex3-*gfp*-*PbECHD1071*^Δ^*^AKL^*, pTAex3-*PbKT298*^ΔP^*^TS2^*-*gfp*, using the corresponding primers as follows: pTAex3-GFP-PbACL75-*Nde*I-FP and pTAex3-GFP-PbACL75ΔPKL-RP, pTAex3-GFP-PbACO323-*Nde*I-FP/pTAex3-GFP-PbACOX323ΔSKL-RP, pTAex3-GFP-PbDECR799-*Nde*I-FP/pTAex3-GFP-PbDECR799ΔSKL-RP, pTAex3-GFP-PbECI1007-*Nde*I-FP/pTAex3-GFP-PbECI1007ΔSHL-RP, pTAex3-GFP-PbECHD1071-*Nde*I-FP/pTAex3-GFP-PbECHD1071ΔAKL-RP, pTAex3-PbKT298-GFP-*Kpn*I-FP/pTAex3-PbKT298ΔPTS2-GFP-RP. All vectors were confirmed by DNA sequencing (Sangon Biotech Shanghai, China).

*Fungal Transformation and Cultivation*: The plasmids were used to transform *A*o_M-2-3_ by the protoplast-polyethylene glycol method as described previously^[1-2]^. Briefly, the linear DNA containing the target gene expression cassette was PCR amplified or enzyme digested from an appropriate plasmid. The spores of *A*o_M-2-3_ were collected and inoculated to 50 mL DPY liquid medium (2% dextrin, 1% polypeptone, 0.5% yeast extract, 0.5% KH_2_PO_4_, and 0.05% MgSO_4_,). After growing at 30°C, 180 rpm for 18 h, mycelia were collected by centrifugation at 8000 *×g* for 5 min, and then used for preparation of protoplasts with 1% lywallzyme, 1% cellulase, and 0.5% lysozyme in 0.8 M NaCl solution by gentle shaking at 28°C for 1-2 h. Protoplasts were filtered and centrifuged at 2500 *×g* for 5 min. The recovered protoplasts were diluted to 1 × 10^8^ mL^-1^ in Solution I (0.8 M NaCl, 10 mM CaCl_2_, 50 mM Tris-HCl, pH 7.5). Subsequently, 10 µg (in 10 µL ddH_2_O) of linear DNA fragment was added into 120 µL each aliquot of protoplasts. The mixtures were incubated on ice for 2 min, 1 mL of Solution II (60% PEG 6000, 0.8 M NaCl, 50 mM CaCl_2_, 50 mM Tris-HCl, pH 7.5) was then added and further incubated at room temperature for 20-30 min. CDS soft-top agar (7 mL, 3.5% Czapek-Dox broth, 0.7% agar, 1 M sorbitol) was added to the protoplasts mixtures and poured onto Czapek-Dox plates supplemented with 1 M sorbitol, and incubated at 30°C for 5-7 d. Single colonies of transformants were picked and transferred onto fresh Czapek-Dox plates for another 3 d cultivation^[3]^.

The genotype of each transformant was evaluated by PCR with the specific primers (Supplementary Table 7). The confirmed transformant was inoculated into 100 mL CMP medium (Czapek-Dox supplemented with 3% maltose for induction and 1% peptone) to induce gene expression under the *α*-amylase promoter in a 250 mL Erlenmeyer flask for 3-4 d at 30°C, 180 rpm.

*Steady-state Kinetic Analysis*: The kinetic analysis of the PbACL75, PbACL1224 and PbACL891 were performed by following a previous procedure with minor modifications. Briefly, a standard assay contained 0.2 μM PbACLs, 2 mM ATP, 2 mM CoA, 10 mM MgCl_2_, 1 mM dithiothreitol (DTT), and 0.05% Triton X-100, varying concentrations of substrates (0-300 μM) in 200 μL reaction buffer. The boiling-inactivated PbACLs was added in the control experiment. Each reaction was initiated by adding PbACLs and carried out at 30°C for 0, 15, 30, 45, 60 s on a 96-well plate. The three parallel experiments reactions were treated with the malachite green phosphate assay kit and detected by microplatereader. The kinetic analysis of the PbACOX323 and PbECHD1071 were determined by HPLC. Briefly, a standard assay contained 5 μM PbACOX323/PbECHD1071, 2 mM FAD/NAD^+^, varying concentrations of substrates **2**-CoA/**10**-CoA (0-300 μM) in 100 μL reaction buffer. The boiling-inactivated PbACOX323/PbECHD1071 was added in the control experiment. Each reaction was initiated by adding PbACOX323/ PbECHD1071 and carried out at 30°C for 0, 10, 20, 30, 40 s in 1.5 mL Eppendorf tube. Add an equal volume of methanol to the reaction system to terminate the reaction. The kinetic analysis of the PbDECR799 was determined by NADPH consumption, which was monitored at 340 nm using the microplatereader. Obtain the standard curve of NADPH at a wavelength of 340 nm by setting different concentrations of NADPH. Finally, the triplicated data (mean ± SD) were fitted to the Michaelis-Menten equation for calculating the *k*_cat_ and *K*_m_ values of different substrate using Origin 8.5.

*Statistical Analysis*: All statistical analysis was conducted using Prism 8.0 (GraphPad Software). All graphs depict mean ± SD unless otherwise indicated. Statistical significances were denoted as not significant (ns, *p* > 0.05, * *p* < 0.05). To assess the statistical significance of single metabolites between two groups, unpaired T-test was used. Enzyme kinetic curves were were fitted to the Michaelis-Menten equation for calculating the *k*_cat_ and *K*_m_ values of different substrate using Origin 8.5.

**Figure S1.** The peroxisomal *β*-oxidation process is partly involved in the biosynthesis of benzoic acid, C24 bile acid and jasmonic acid. A), the biosynthesis of benzoic acid: benzoic acid biosynthesis occurs through multiple routes that arise from the phenylpropanoid pathway and begins with the deamination of _L_-phenylalanine (Phe) to *trans*-cinnamic acid (CA) by phenylalanine ammonia lyase (PAL). CA is converd to cinnamoyl-CoA (CA-CoA), by a peroxisomal cinnamate-CoA ligase (PhCNL, ACL). Subsequent formation of benzoyl-CoA (BA-CoA) requires the participation of the bifunctional protein *pBa*PhCHD (ECHD) and 3-ketoacyl-CoA thiolase *pBa*PhKAT1 (KT)^[4]^. B), the biosynthesis of cholic acid: the cholic acid biosynthesis begins with the conversion of cholesterol into 7α-hydroxycholesterol by cholesterol 7α-hydroxylase CYP7A1 (CYP). Subsequently, 7α-hydroxycholesterol is catalyzed by 3*β*-hydroxy-Δ^5^-C27-steroid oxidoreductase (HSD3B7), 12α-hydroxylase (CYP8B1), Δ^4^-3-oxosteroid 5*β*-reductase (AKR1D1), 3α-hydroxysteroid dehydrogenase (AKR1C4), and CYP27A1 to generate 3α, 7α, 12α-trihydroxycholestanoic acid (THCA). THCA is converd to (25*R*)-THCA-CoA by the very long-chain acyl-CoA synthetase (VLCS) or bile acyl-CoA synthetase (BACS). (25*R*)-THCA-CoA is future catalyzed by *α*-methylacyl-CoA racemase (AMACR) to form (25*S*) THC-CoA. Its activity is necessary, because the peroxisomal *β*-oxidation system can only handle (25*S*)-THC-CoA. (25*S*)-THC-CoA is catalyzed by *β*-oxidation enzyme branched chain acyl-CoA oxidase *hCa*ACX (ACOX), D-bifunctional protein *hCa*DBP, (homolog of ECHD), sterol carrier protein X *hCa*SCPx (homolog of KT) to form choloyl-CoA^[5]^. C), the biosynthesis of jasmonic acid: the jasmonic acid biosynthesis begins with the conversion of linolenic acid into 3-oxo-2(2’(*Z*)-pentenyl)-cyclopentane-1-octanoic acid (OPC-8:0) by oxygenated by lipoxygenase (LOX), allene oxide synthase (AOS) and allene oxide cyclase (AOC), 12-oxophytodienoate reductase 3 (OPR3). OPC-8:0 is converd to OPC-8:0-CoA by CoA ligase. OPC-8:0-CoA THC-CoA is catalyzed by *β*-oxidation enzyme acyl-CoA oxidase *pJa*ACOX (ACOX), D-bifunctional protein *pJa*MFP, (homolog of ECHD), 3-ketoacyl-CoA thiolase *pJa*KAT (KT) to form jasmonic acid by three cycles^[6]^.

**Figure S2.** Gene organization of the reported biosynthetic gene clusters of **1**^[2, 7-9]^.

**Figure S3.** The biosynthetic pathway of **1**^[1]^. Briefly, the biosynthesis of **1** begins with the production of 5-methylorsellinic acid (5-MOA) by the cytosolic PKS-MpaC’, which is converted to 3,5-dihydroxy-7-(hydroxymethyl)-6-methylbenzoic acid (DHMP, **23**) by the endoplasmic reticulum (ER) membrane-bound natural fusion enzyme MpaDE’ consisted by a cytochrome P450 and a hydrolase. Next, the Golgi-associated prenyltransferase MpaA’ introduces the farnesyl pyrophosphate (FPP) group to **23** to yield 4-farnesyl-3,5-dihydroxy-6-methylphthalide (FDHMP, **24**)^[10-12]^. The terminal C19=C20 double bond of **24** is then oxidatively cleaved by the ER-bound globin-like oxygenase MpaB’ to form the three-carbon shortened intermediate FDHMP-3C, which is further methylated at C4 by the cytosolic methyltransferase MpaG’ to MFDHMP-3C. The peroxisomal acyl-CoA ligase PbACL891 subsequently activates **2** to MFDHMP-3C-CoA (**2**-CoA) inside peroxisomes. This activation is the critical step to enter the peroxisomal *β*-oxidation and **2**-CoA is proposed to undergo a successive chain-shortening process to produce MPA-CoA (**1**-CoA). The final production of **1** is controlled by the peroxisomal "gatekeeper" *α*/*β* acyl-CoA hydrolase MpaH’.


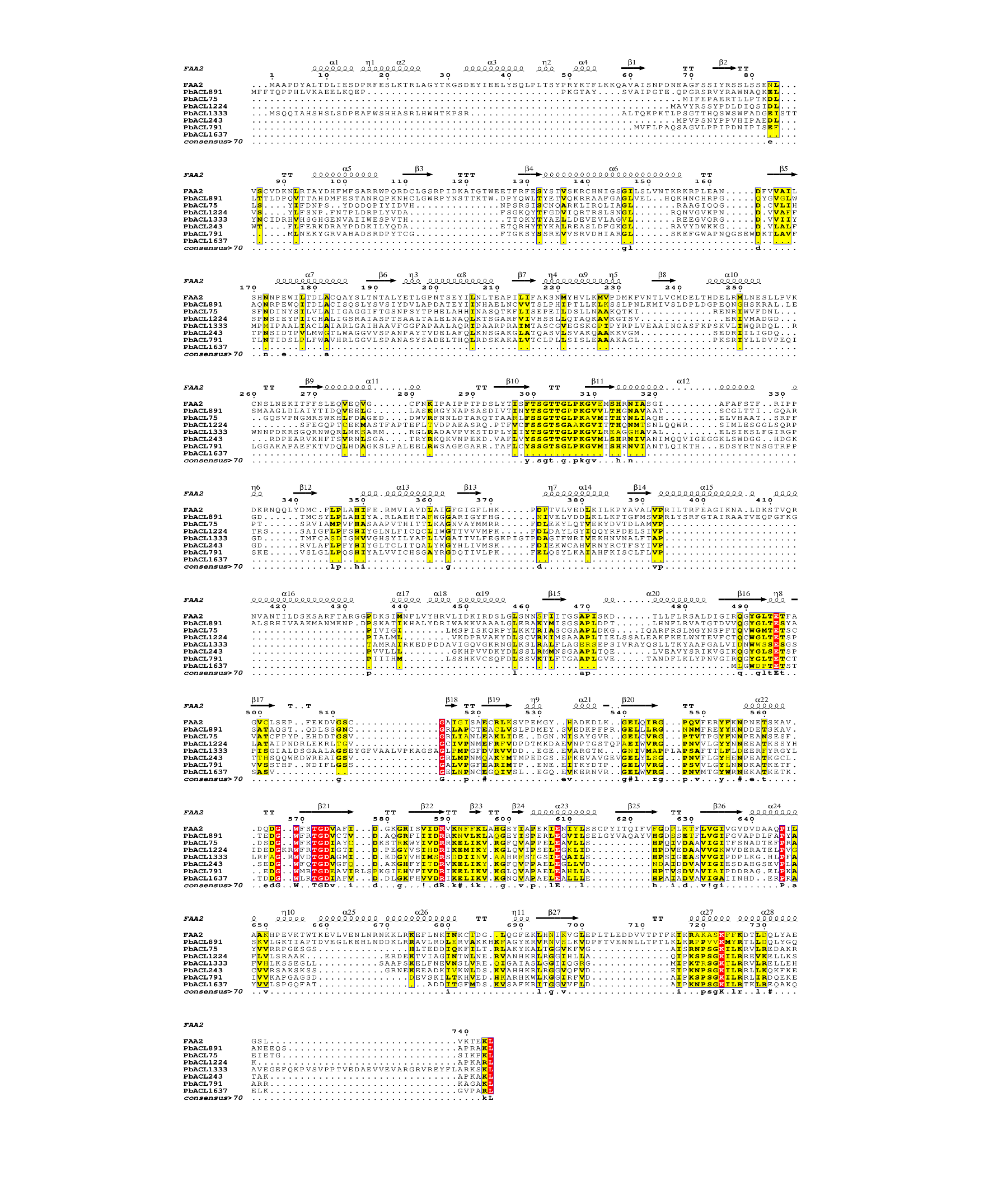


**PTS1**

**Figure S4.** Protein sequence alignment of seven CoA ligases from *Pb*864 with FAA2 (Gene ID: 856734) from *S. cerevisiae* S288C^[13]^ (Red boxes: PTS1-Type I Peroxisome target signal).


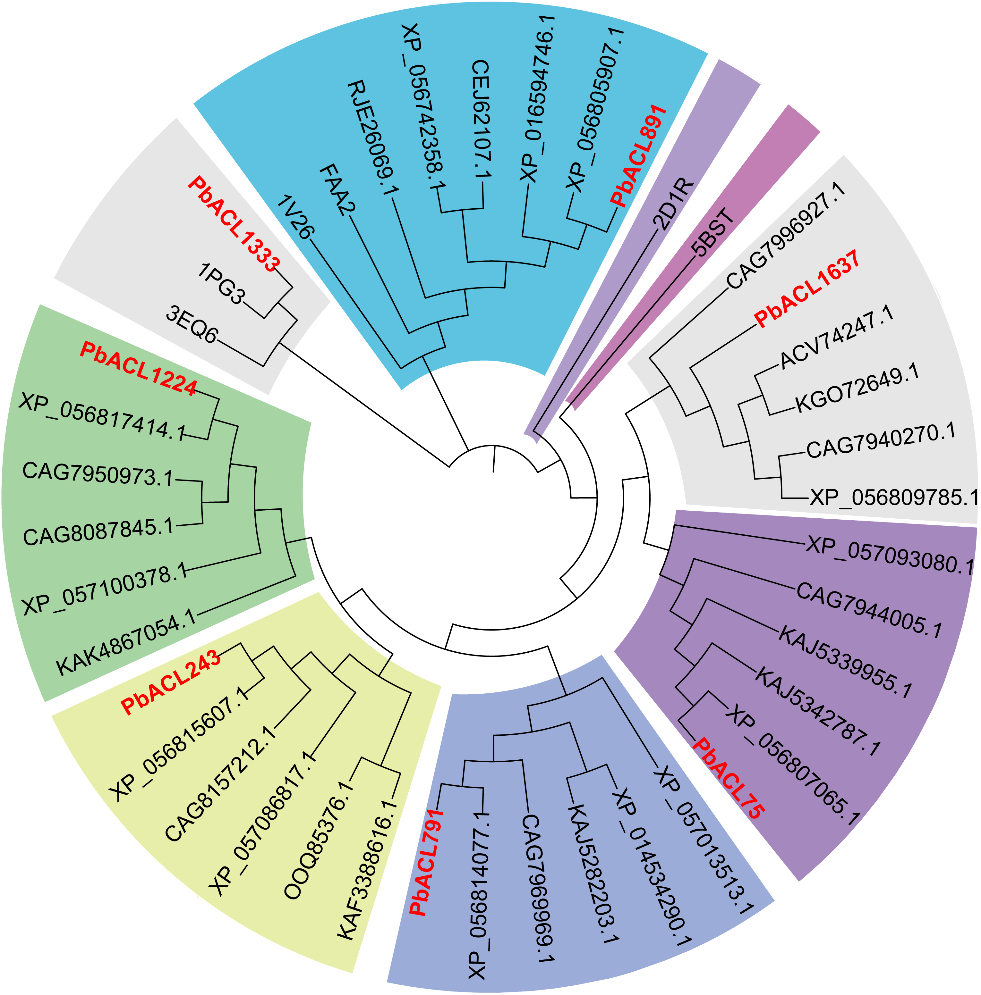


**Figure S5.** The phylogenetic tree of the representative acyl-CoA ligase. The following sequences were obtained by comparative search in the NCBI database using PbACL75, PbACL1224, PbACL891, PbACL791, PbACL243, PbACL1333 and PbACL1637 (from *Pb*864) as probes, respectively. FAA2, from *S. cerevisiae* S288C, long-chain fatty acid CoA ligase 2; 1V26, from *Thermus thermophiles*, long-chain fatty acyl-CoA synthetase; 3EQ6, from *Homo sapiens*, medium-chain fatty acyl-CoA synthetase; 1PG3, from *Salmonella enterica*, acetyl CoA synthetase; 5BST, from *Nicotiana tabacum*, 4-coumarate CoA ligase; 2D1R, from *Nipponoluciola cruciate*, Luciferase; PCL, from *Penicillium chrysogenum*, phenylacetyl-CoA ligase *phl*; CefD1, from *Acremonium chrysogenum*, isopenicillin N-CoA (IPN-CoA) synthetase; XP_056807065.1, from *P. brevicompactum* IBT 35665, AMP-dependent synthetase/ligase; KAJ5342787.1, from *P. brevicompactum*, AMP-dependent synthetase/ligase; KAJ5339955.1, from *P. brevicompactum*, AMP-dependent synthetase/ligase; CAG7944005.1, from *Penicillium salami*, unknown functional protein; XP_057093080.1, from *Penicillium soppii*, AMP-dependent synthetase/ligase; XP_056805907.1, from *P. brevicompactum*, uncharacterized protein; XP_016594746.1, from *Penicillium expansum*, AMP-dependent synthetase/ligase; CEJ62107.1, from *Penicillium brasilianum*, putative long-chain acyl-CoA synthetase; XP_056742358.1, from *Penicillium atrosanguineum*, citrate synthase; RJE26069.1, from *Aspergillus sclerotialis*, acyl-CoA synthetase; XP_056817414.1, from *P. brevicompactum*, AMP-dependent synthetase/ligase; CAG7950973.1, from *Penicillium olsonii*, unknown functional protein; CAG8087845.1, from *P salami*, unknown functional protein; XP_057100378.1, from *Penicillium soppii*, AMP-dependent synthetase/ligase; KAK4867054.1, from *P. expansum*, unknown functional protein; XP_056815607.1, from *P. brevicompactum*, uncharacterized protein; CAG8157212.1, from *P. salami*, unknown functional protein; XP_057086817.1, from *Penicillium robsamsonii*, AMP-dependent synthetase/ligase; OOQ85376.1, from *P. brasilianum*, phenylacetyl-CoA ligase; KAF3388616.1, from *Penicillium rolfsii*, putative 4-coumarate-CoA ligase 1; XP_056814077.1, from *P brevicompactum*, phenylacetyl-CoA ligase; CAG7969969.1, from *Penicillium salami*, unknown functional protein; KAJ5282203.1, from *Penicillium chrysogenum*, phenylacetyl-CoA ligase; XP_014534290.1, from *Penicillium digitatum* Pd1, phenylacetyl-CoA ligase; XP_057013513.1, from *Penicillium subrubescens*, Acyl-CoA ligase inpC; XP_056809785.1, from *P. brevicompactum*, uncharacterized protein; CAG7996927.1, from *P. olsonii*, uncharacterized protein; CAG7940270.1, from *Penicillium salami*, uncharacterized protein; KGO72649.1, from *Penicillium italicum*, AMP-dependent synthetase/ligase; ACV74247.1, from *P. chrysogenum*, phenylacetyl-CoA ligase.


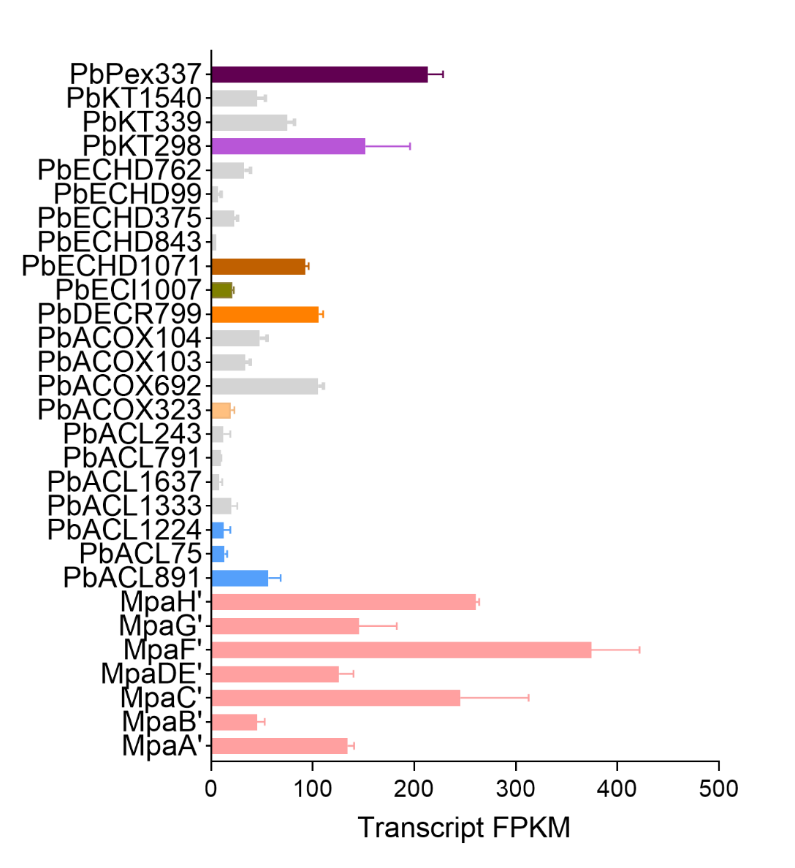


**Figure S6.** Comparison of transcript level of enzymes involved in the biosynthesis of **1** (FPKM: Fragments Per Kilobase of exon model per Million mapped fragments).


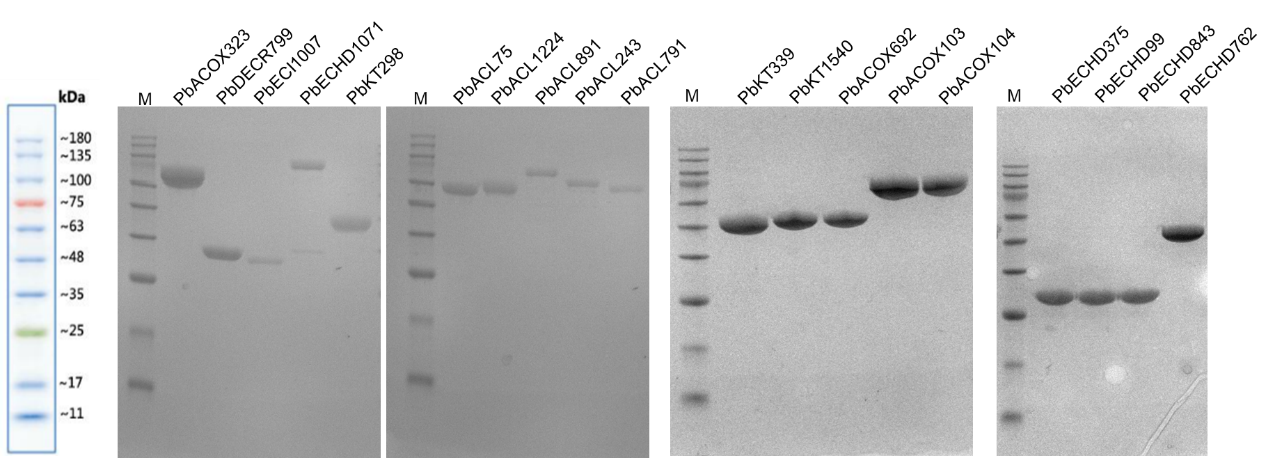


**Figure S7.** SDS-PAGE analysis of purified PbACLs, PbACOXs, PbDECR799, PbECI1007, PbECHDs and PbKTs from *Pb*864. (PbACL75: 61 kDa; PbACL1224: 62.4 kDa; PbACL891: 78.1 kDa; PbACL243: 62.5 kDa; PbACL791: 62.9 kDa; PbACOX323: 78 kDa; PbACOX692: 56 kDa; PbACOX103: 76 kDa; PbACOX104: 78 kDa; PbECHD1071: 101 kDa; PbECHD762: 51 kDa; PbECHD375: 28 kDa; PbECHD99: 29 kDa; PbECHD843: 31 kDa; PbKT298: 48 kDa; PbKT339: 48 kDa; PbKT1540: 49 kDa; PbDECR799: 38 kDa; PbECI1007: 32 kDa.)


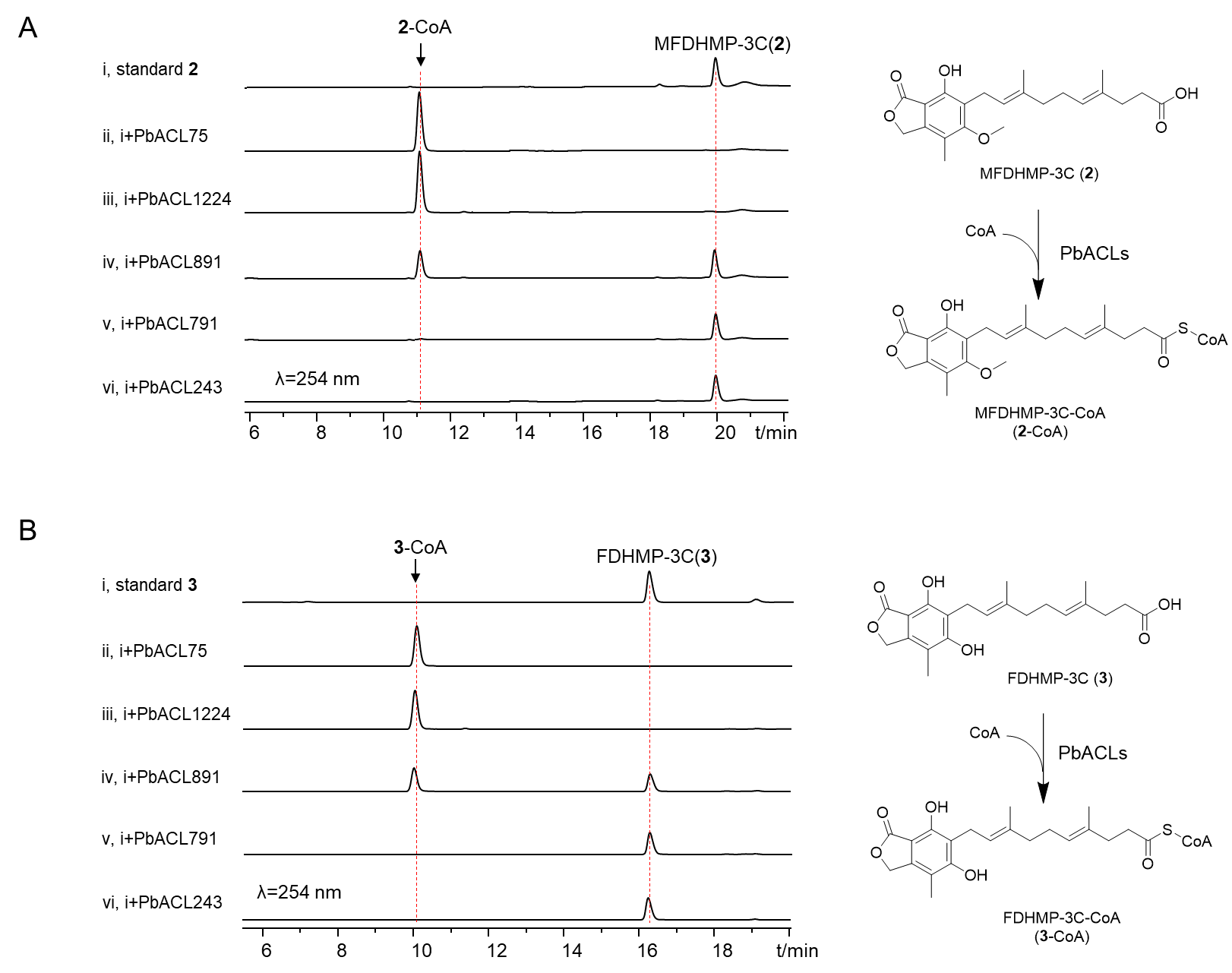


**Figure S8.** HPLC analysis (254 nm) reaction of (M)FDHMP-3C (**2**/**3**) with CoA ligases. A), (i) Standard of MFDHMP-3C; (ii-vi) The reaction of **2** with PbACL75, PbACL1224, PbACL891, PbACL791 and PbACL243, respectively. B), (i) Standard of FDHMP-3C; (ii-vi) The reaction of **3** with PbACL75, PbACL1224, PbACL891, PbACL791 and PbACL243 (from *Pb*864) in the presence of ATP, CoA, and Mg^2+^, respectively.


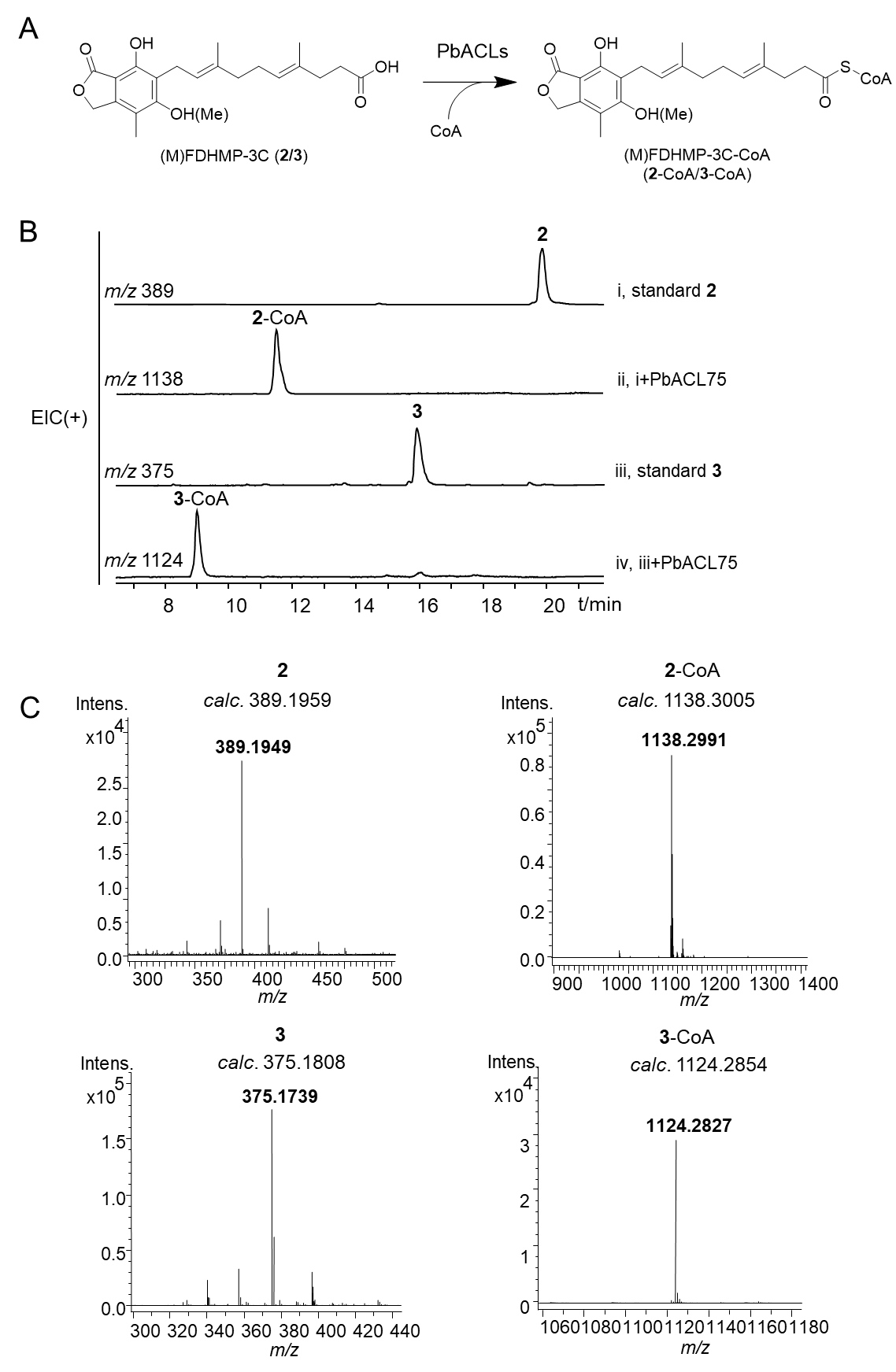


**Figure S9.** LC-HRMS analysis of the *in vitro* conversion from (M)FDHMP-3C (**2/3**) to (M)FDHMP-3C-CoA (**2**-CoA/**3**-CoA) catalyzed by purified PbACL75. A), The reaction schematic from **2**/**3** to **2**-CoA/**3**-CoA. B), (i) The EIC of MFDHMP-3C in the control reaction with boiled PbACL75; (ii) The EIC of MFDHMP-3C-CoA (**2**-CoA) in the reaction with PbACL75; (iii) The EIC of FDHMP-3C in the control reaction with boiled PbACL75; (iv) The EIC of FDHMP-3C-CoA (**3**-CoA) in the reaction with PbACL75. C), The *m/z* values of **3**-CoA: [M+H]^+^= 1124.2827 (*obs*.), *calc*. 1124.2854; **3**: [M+H]^+^ = 375.1739 (*obs*.), *calc*. 375.1808; **2**-CoA: [M+H]^+^= 1138.2991 (*obs*.), *calc*. 1138.3005; and **2**: [M+H]^+^ = 389.1949 (*obs*.), *calc*. 389.1959.


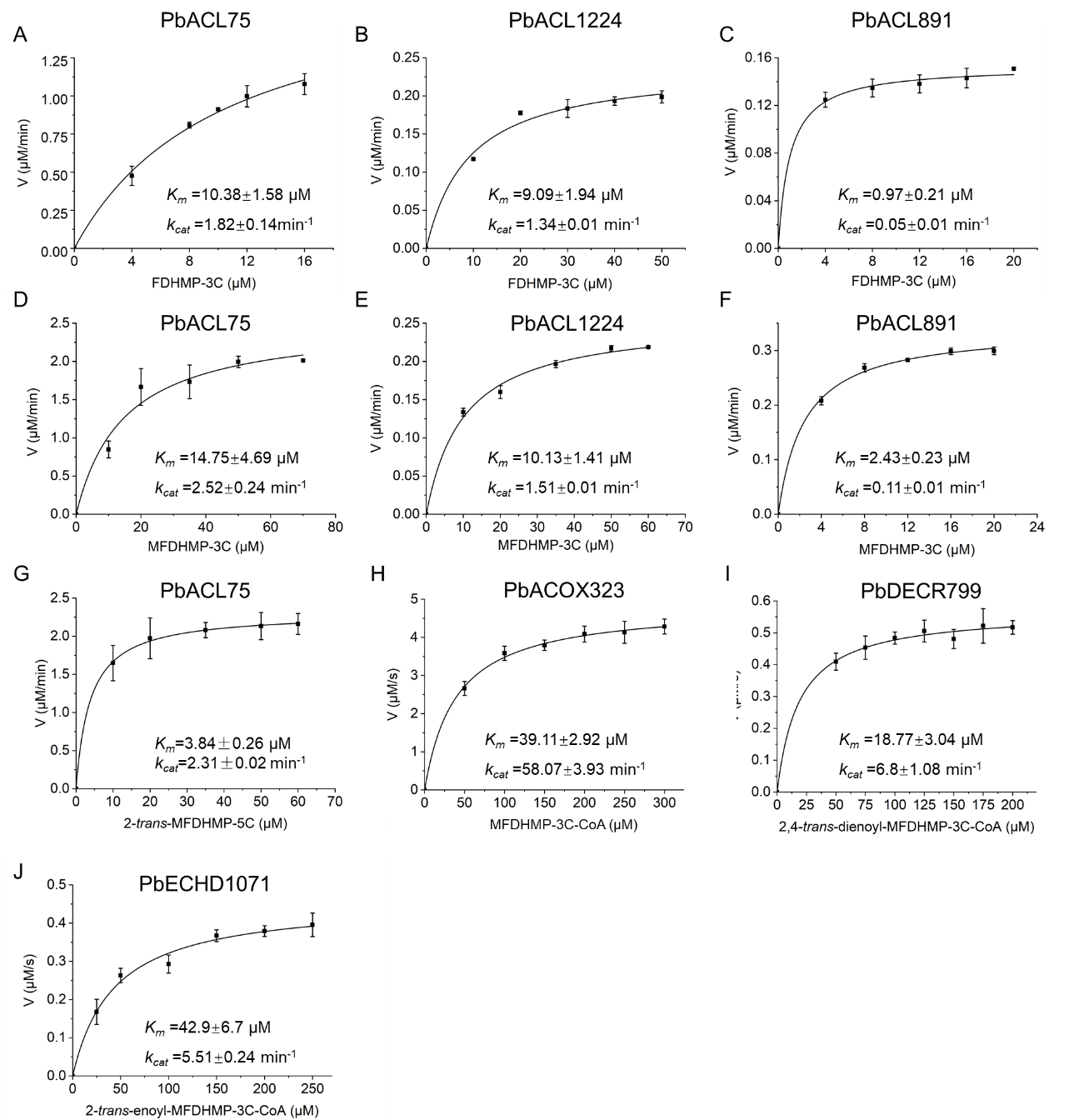


**Figure S10** The steady-state kinetic curves of PbACLs PbACOX323, PbDECR799, and PbECHD1071. A-C), The steady-state kinetic curves of PbACL75, PbACL1224 and PbACL891 toward **3**. D-F), The steady-state kinetic curves of PbACL75, PbACL1224 and PbACL891 toward **2**. G), The steady-state kinetic curves of PbACL75 toward **4**. H), The steady-state kinetic curves of PbACOX323 toward **2**-CoA. I), The steady-state kinetic curves of PbDECR799 toward **5**-CoA. J), The steady-state kinetic curves of PbECHD1071 toward **10**-CoA.


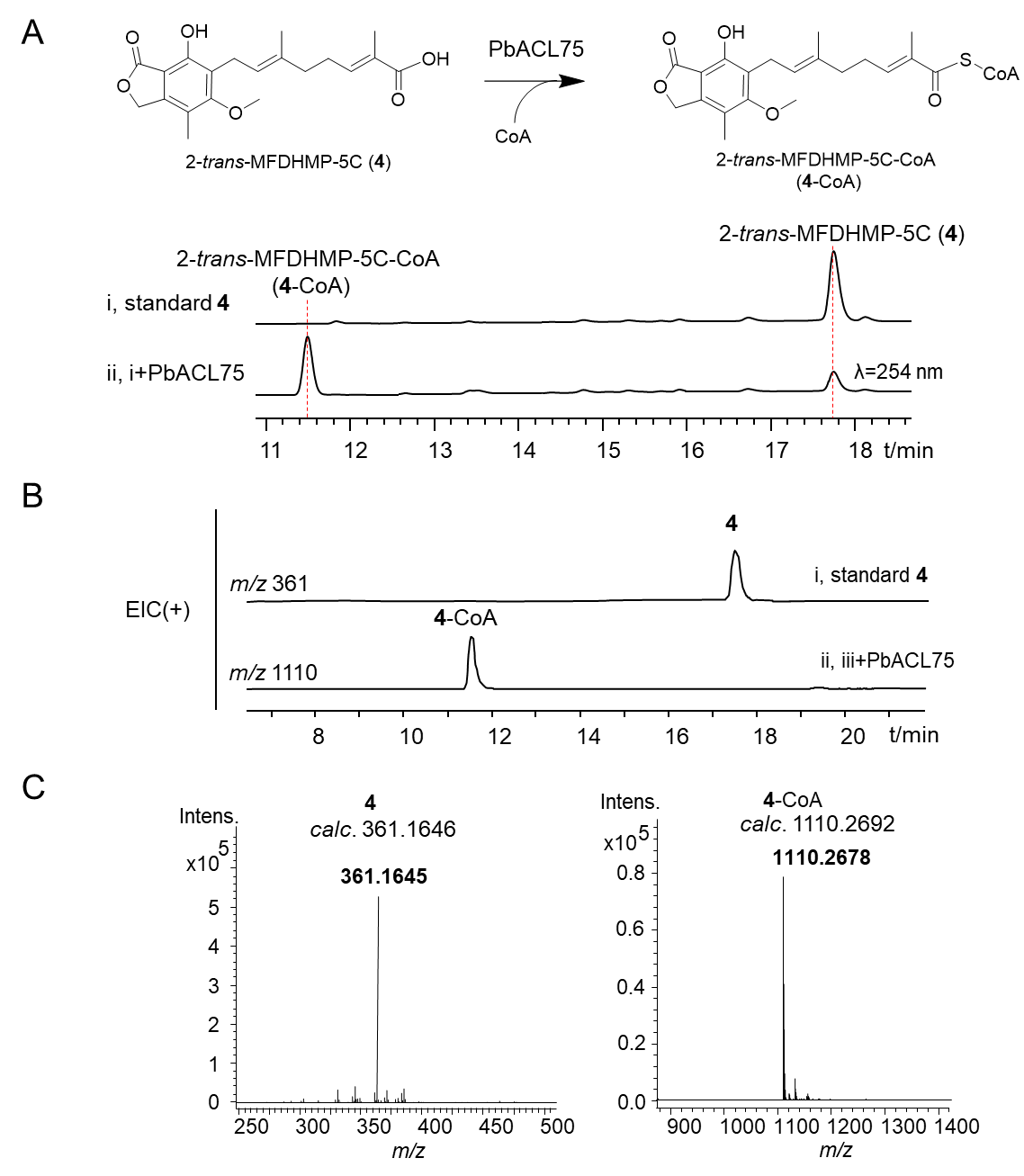


**Figure S11.** HPLC and LC-HRMS analysis (254 nm) reaction of 2-*trans*-MFDHMP-5C (**4**) with PbACL75. A), HPLC analysis (254 nm) reaction of **4** with PbACL75. (i) Standard of **4**; (ii) The reaction of **4** with PbACL75. B), (i) The EIC of 2-*trans*-MFDHMP-5C (**4**) in the control reaction with boiled PbACL75; (ii) The EIC of 2-*trans*-MFDHMP-5C-CoA (**4**-CoA) in the reaction with PbACL75. C), The *m/z* values of **4**-CoA: [M+H]^+^= 1110.2678 (*obs*.), *calc*. 1110.2692; and **4**: [M+H]^+^ = 361.1645 (*obs*.), *calc*. 361.1646.


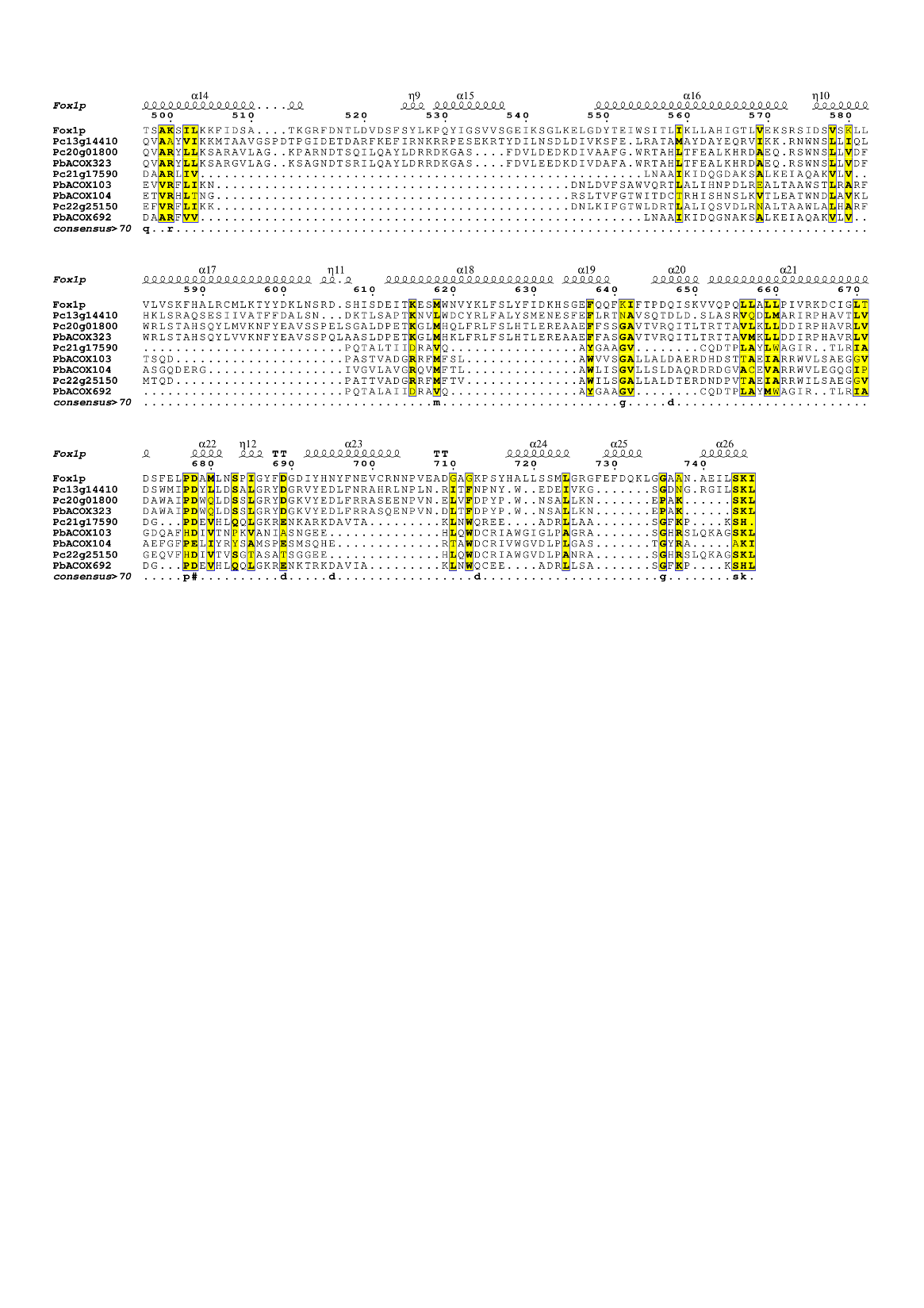

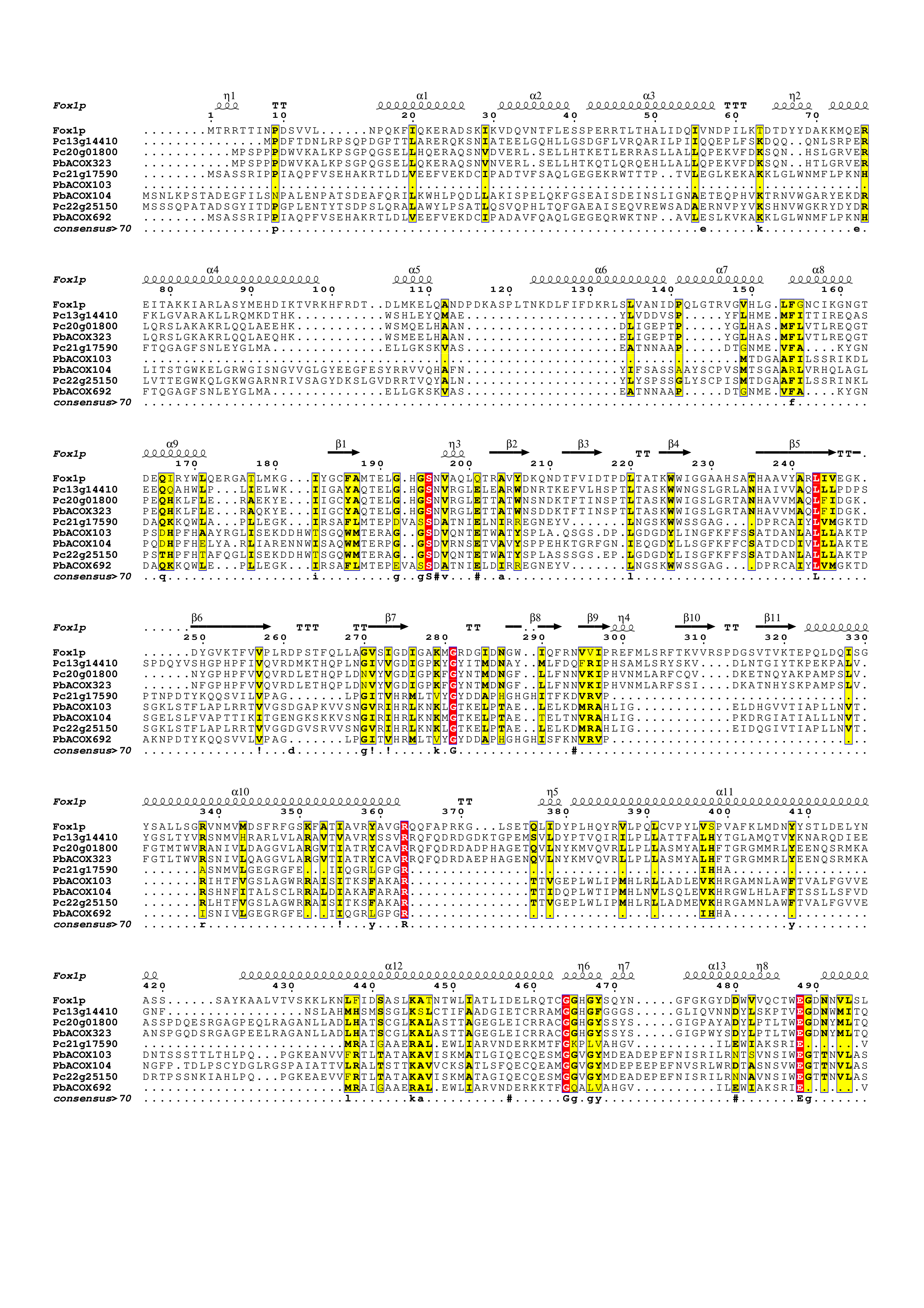


**PTS1**

**Figure S12.** Protein sequence alignment of PbACOXs from *Pb*864 with Fox1p/Pox1p (Gene ID: 852667) from *S. cerevisiae* S288C^[14]^ and Pc13g14410, Pc21g01800, Pc21g17590, Pc22g25150 from *P. chrysogenum* (Red boxes: PTS1-Type I Peroxisome target signal).


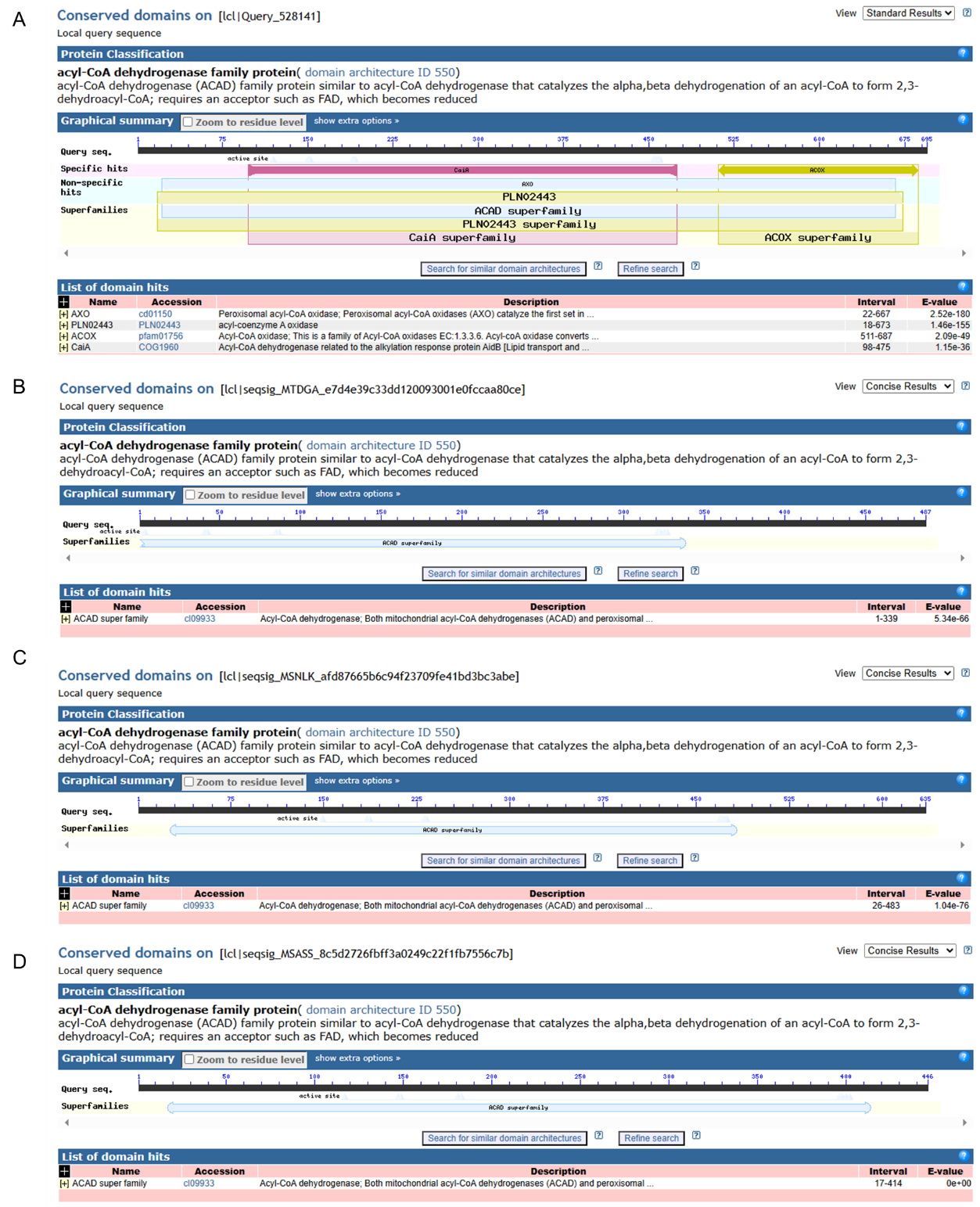


**Figure S13.** Pfam analysis the conserved domains of ACOX: PbACOX323 (A), PbACOX103 (B), PbACOX104 (C), PbACOX692 (D).


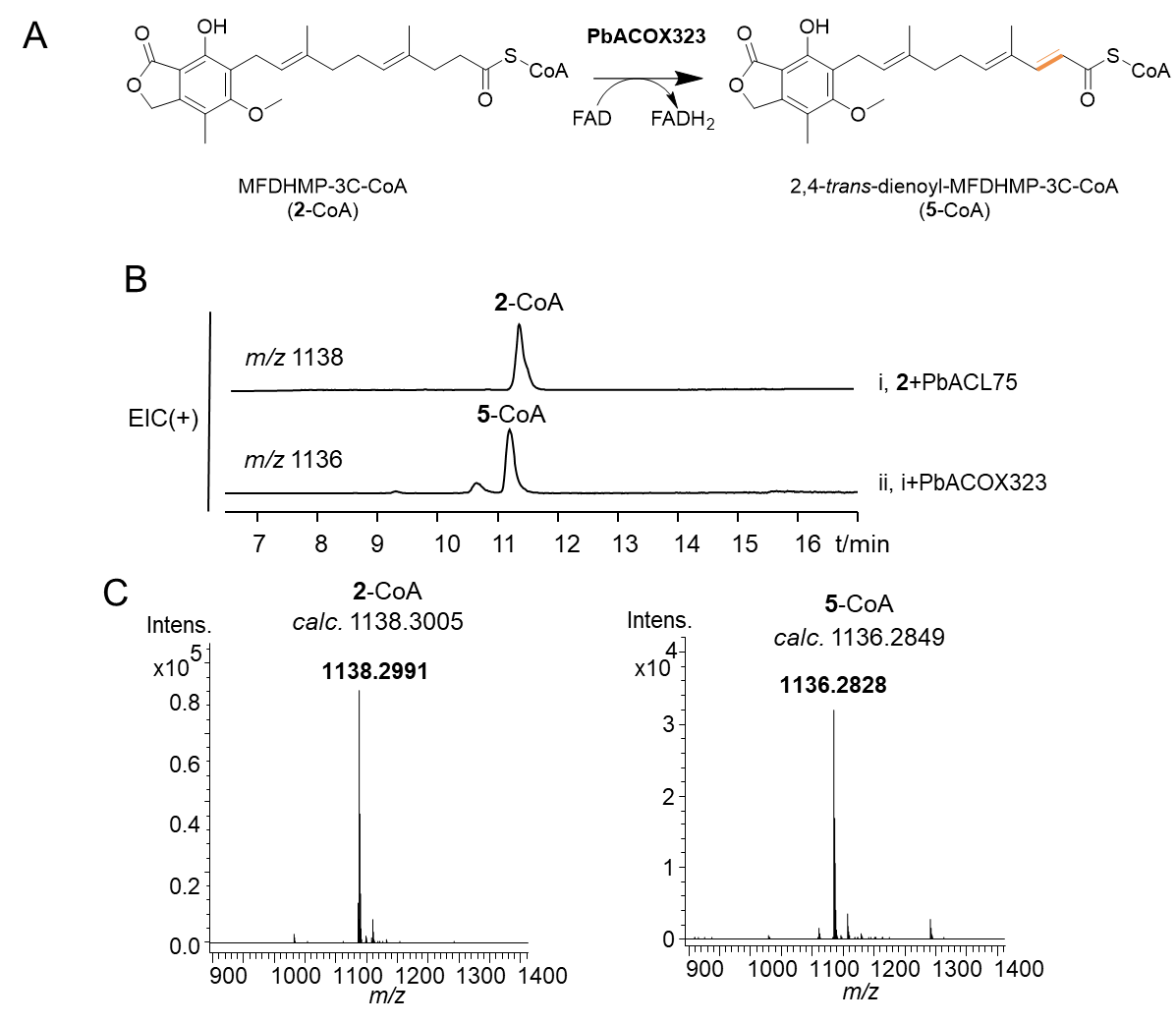


**Figure S14.** LC-HRMS analysis of the *in vitro* conversion from MFDHMP-3C-CoA (**2**-CoA) to **5**-CoA. A), The reaction schematic from **2**-CoA to **5**-CoA. B), (i) The EIC of **2**-CoA in the control reaction with boiled PbACOX323; (ii) The EIC of **5**-CoA in the reaction with PbACOX323. C), The *m/z* values of **2**-CoA, [M+H]^+^= 1138.2991 (*obs*.), *calc*. 1138.3005; **5**-CoA, [M+H]^+^= 1136.2828 (*obs*.), *calc*. 1136.2849.


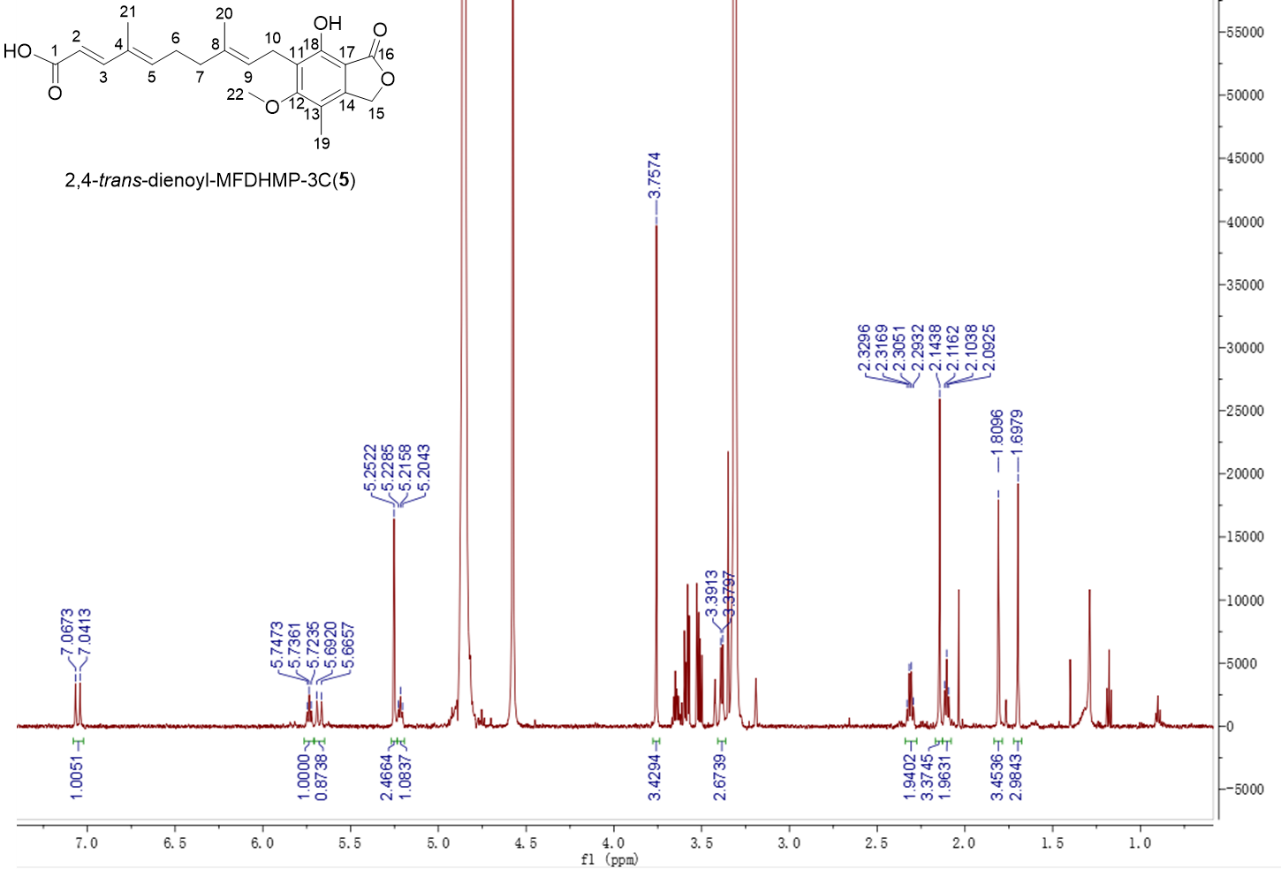


**Figure S15.** ^1^H NMR spectrum of 2,4-*trans*-dienoyl-MFDHMP-3C (**5**) in CD_3_OD (600 MHz).

**
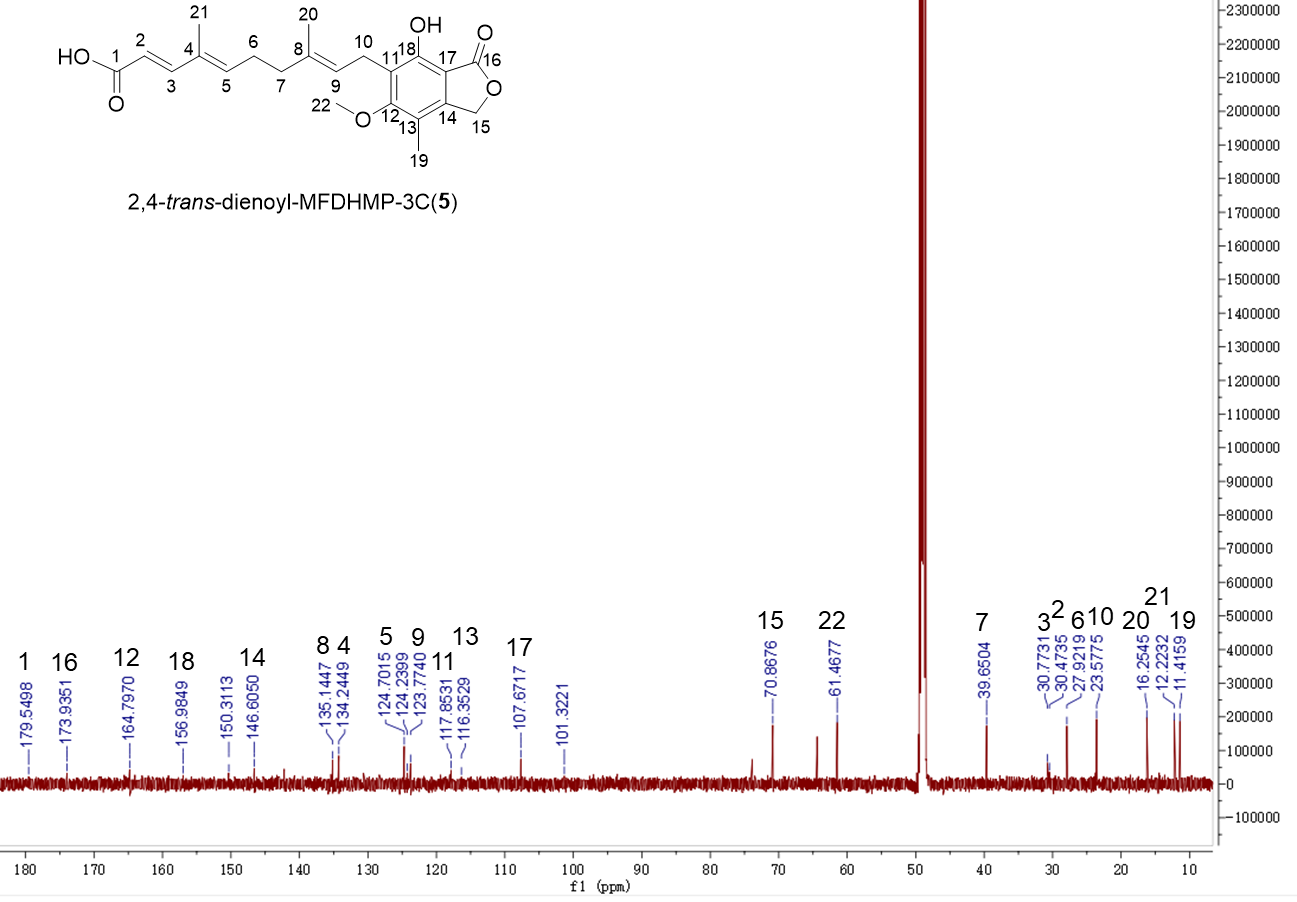
**

**Figure S16.** ^13^C NMR spectrum of 2,4-*trans*-dienoyl-MFDHMP-3C (**5**) in CD_3_OD (150 MHz).


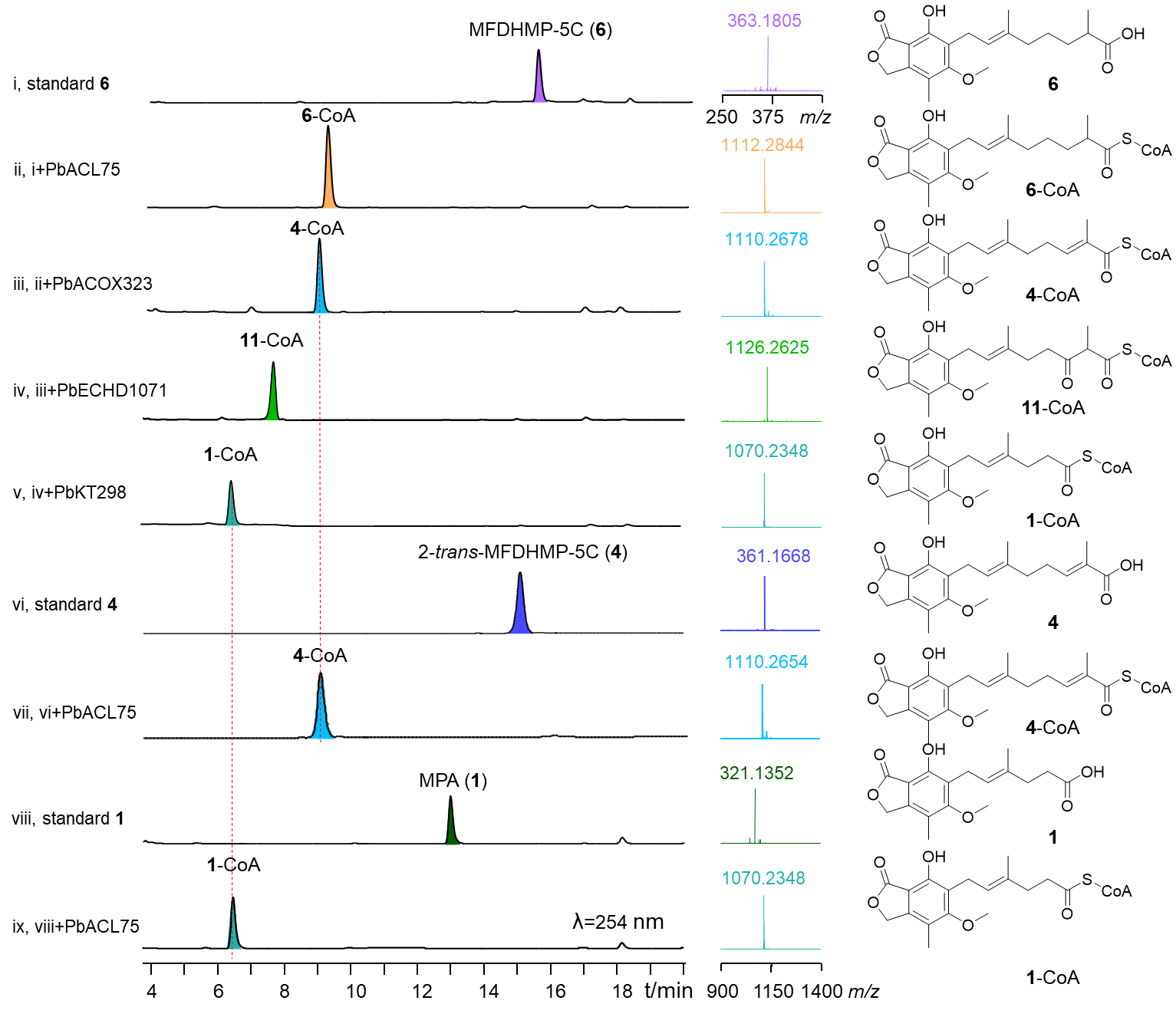


**Figure S17.** HPLC analysis of the *in vitro* conversion from MFDHMP-5C (**6**) to **1**-CoA catalyzed by purified PbACL75, PbACOX323, PbECHD1071 and PbKT298. i) The standard of MFDHMP-5C (**6**); ii) CoA ligation reaction mediated by PbACL75 toward MFDHMP-5C (**6**); iii) Acyl-CoA oxidase PbACOX323 was added into the step (ii) reaction finished mixture, converting **6**-CoA to **4**-CoA; iv) Enoyl-CoA hydratase and 3-hydroxyacyl-CoA dehydrogenase enzyme PbECHD1071 was introduced into the step (iii) reaction finished mixture, resulting in the installation of the 3-keto group of **11**-CoA; v) Addition of 3-ketoacyl-CoA thiolase PbKT298 to the step (iv) reaction finished mixture led to the detection of the two carbon-shorter product **1**-CoA together with Propionyl-CoA (see Figure S42); vi) The standard of 2-*trans*-MFDHMP-5C (**4**); vii) CoA ligation reaction mediated by PbACL75 toward 2-*trans*-MFDHMP-5C (**4**); viii) The standard of **1**; ix) CoA ligation reaction mediated by PbACL75 toward **1**.


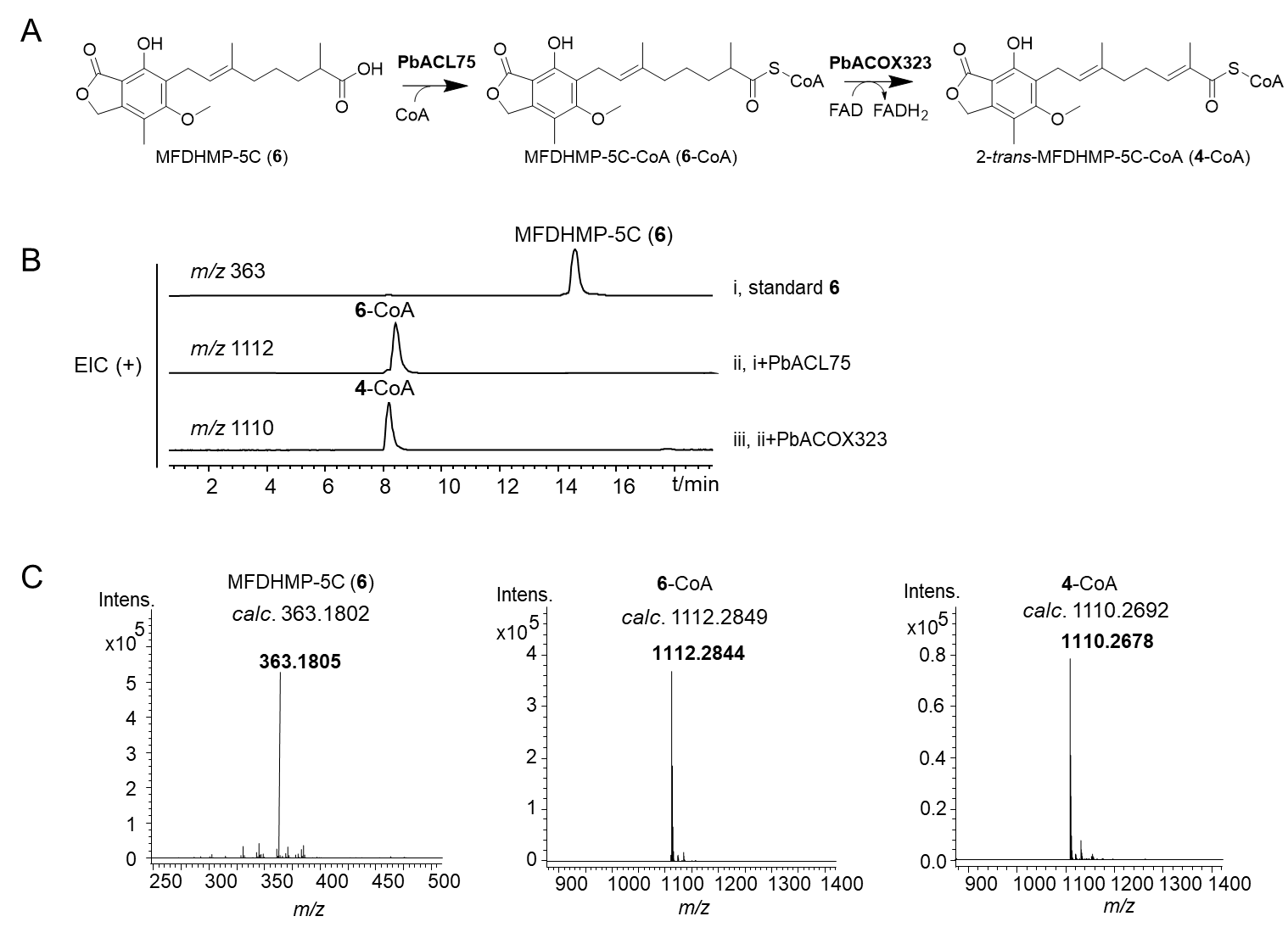


**Figure S18.** LC-HRMS analysis of the *in vitro* conversion from MFDHMP-5C (**6**) to 2-*trans*-FDHMP-5C-CoA (**4**-CoA) catalyzed by purified PbACL75 and PbACOX323. A), The reaction schematic from **6** to **4**-CoA. B), (i) The standard of **6**; (ii) The EIC of **6**-CoA in the reaction with PbACL75; (iii) The EIC of **4**-CoA in the reaction with PbACOX323. C), The *m/z* values of **6**, [M+H]^+^= 363.1805 (*obs*.), *calc*. 363.1802; **6**-CoA, [M+H]^+^= 1112.2844 (*obs*.), *calc*. 1112.2849; **4**-CoA, [M+H]^+^= 1110.2678 (*obs*.), *calc*. 1110.2692.


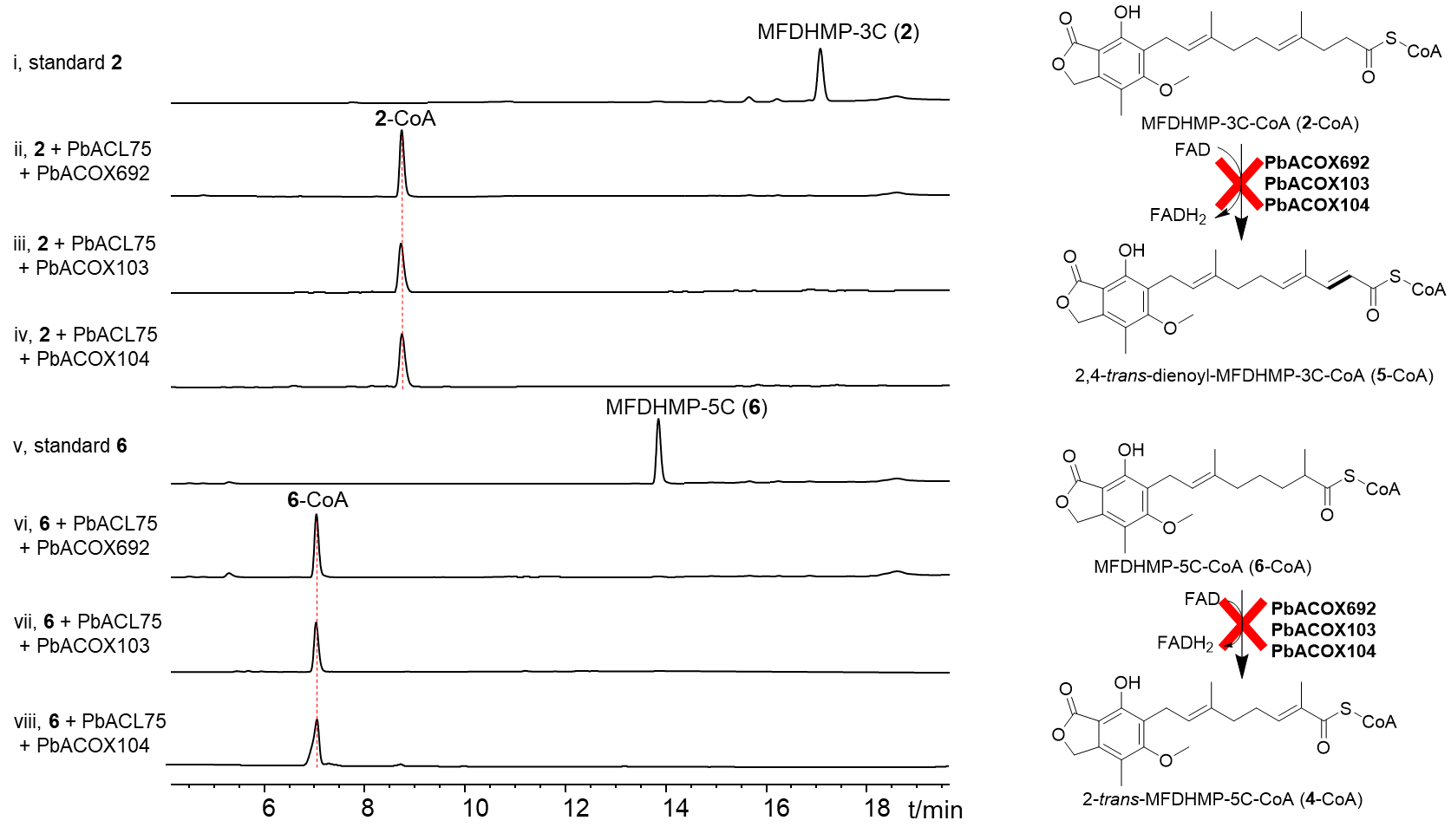


**Figure S19.** HPLC analysis of the *in vitro* conversion from **2**-CoA or **6**-CoA to **5**-CoA or **4**-CoA catalyzed by purified PbACOX692, PbACOX103 and PbACOX104. (i) Standard of MFDHMP-3C; (ii-iv) The reaction of **2** with PbACOX692, PbACOX103 and PbACOX104 in the presence of PbACL75, ATP, CoA, FAD and Mg^2+^, respectively; (v) Standard of MFDHMP-5C (**6**); (vi-viii) The reaction of **6** with PbACOX692, PbACOX103 and PbACOX104 in the presence of PbACL75, ATP, CoA, FAD and Mg^2+^, respectively.


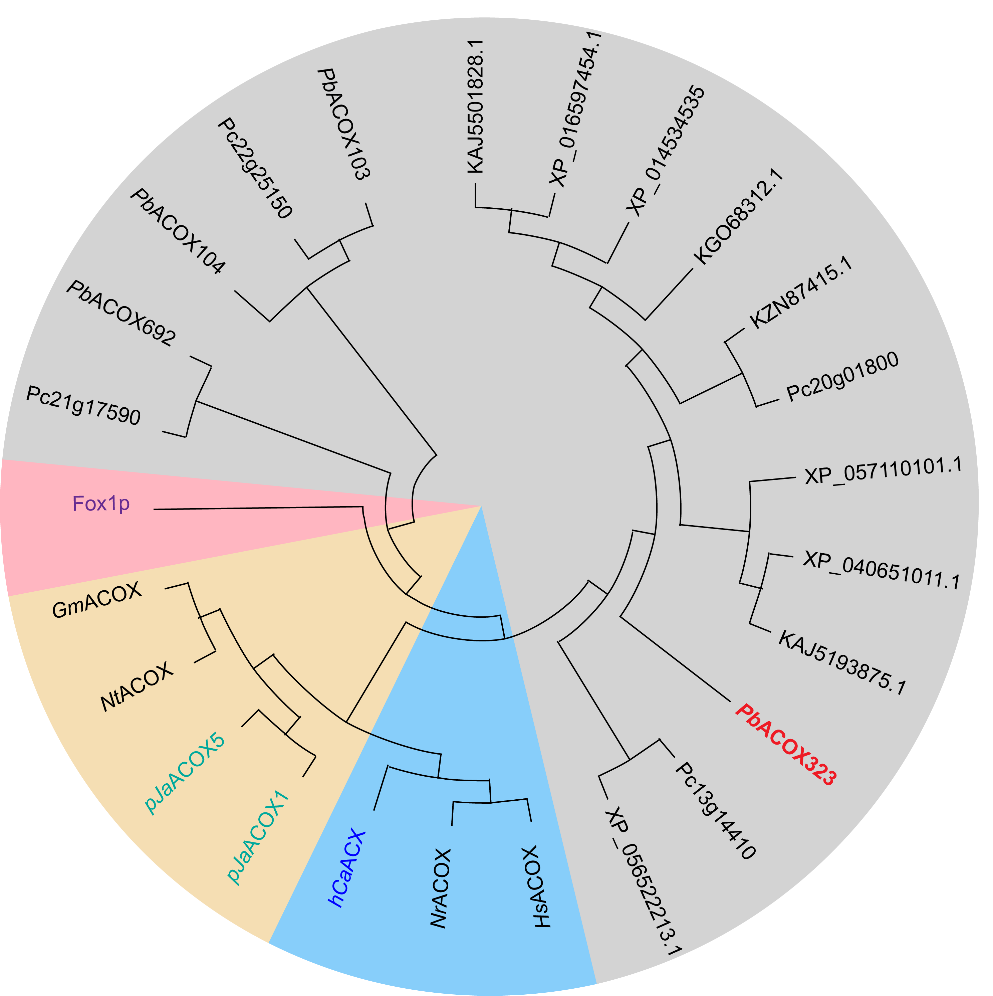


**Figure S20.** The phylogenetic tree of the representative acyl-CoA oxidase. The following sequences were obtained by comparative search in the NCBI database using PbACOX323, PbACOX103, PbACOX104 and PbACOX692 (from *Pb*864) as probes, respectively. Pc13g14410, from *Penicillium chrysogenum*, acyl-CoA oxidase; XP_056522213.1, from *Penicillium bovifimosum*, acyl-CoA oxidase; *Hs*ACOX, from *Homo sapiens*, acyl-CoA oxidase; *Nr*ACOX, from *Norway rat*, acyl-CoA oxidase 1; *hCa*ACX, from *Mus musculus*, acyl-CoA oxidase; *pJa*ACOX1, from *Arabidopsis thaliana*, acyl-CoA oxidase 1; *pJa*ACOX5, from *Arabidopsis thaliana*, acyl-CoA oxidase 5; *Nt*ACOX, from *Nicotiana tabacum*, acyl-CoA oxidase; *Gm*ACOX, from *Glycine max*, acyl-CoA oxidase; Fox1p, from *S. cerevisiae* S288C, acyl-CoA oxidase; Pc21g17590, from *Penicillium chrysogenum*, acyl-CoA oxidase; Pc22g25150, from *Penicillium chrysogenum*, acyl-CoA oxidase; KAJ5501828.1, from *Penicillium expansum*, acyl-CoA oxidase; XP_016597454.1, from *Penicillium expansum*, acyl-CoA oxidase; XP_014534535, from *Penicillium digitatum*, acyl-CoA oxidase; KGO68312.1, from *Penicillium italicum*, acyl-CoA oxidase; KZN87415.1, from *Penicillium chrysogenum*, acyl-CoA oxidase; Pc20g01800, from *Penicillium chrysogenum*, acyl-CoA oxidase; XP_057110101.1, from *Penicillium vulpinum*, acyl-CoA oxidase; XP_040651011.1, from *Penicillium griseofulvum*, acyl-CoA oxidase; KAJ5193875.1, from *Penicillium griseofulvum*, acyl-CoA oxidase.(lightgrey: represents *Penicillium*; lighpink: represents yeast; skyblue: represents animal; wheat: represents plant)


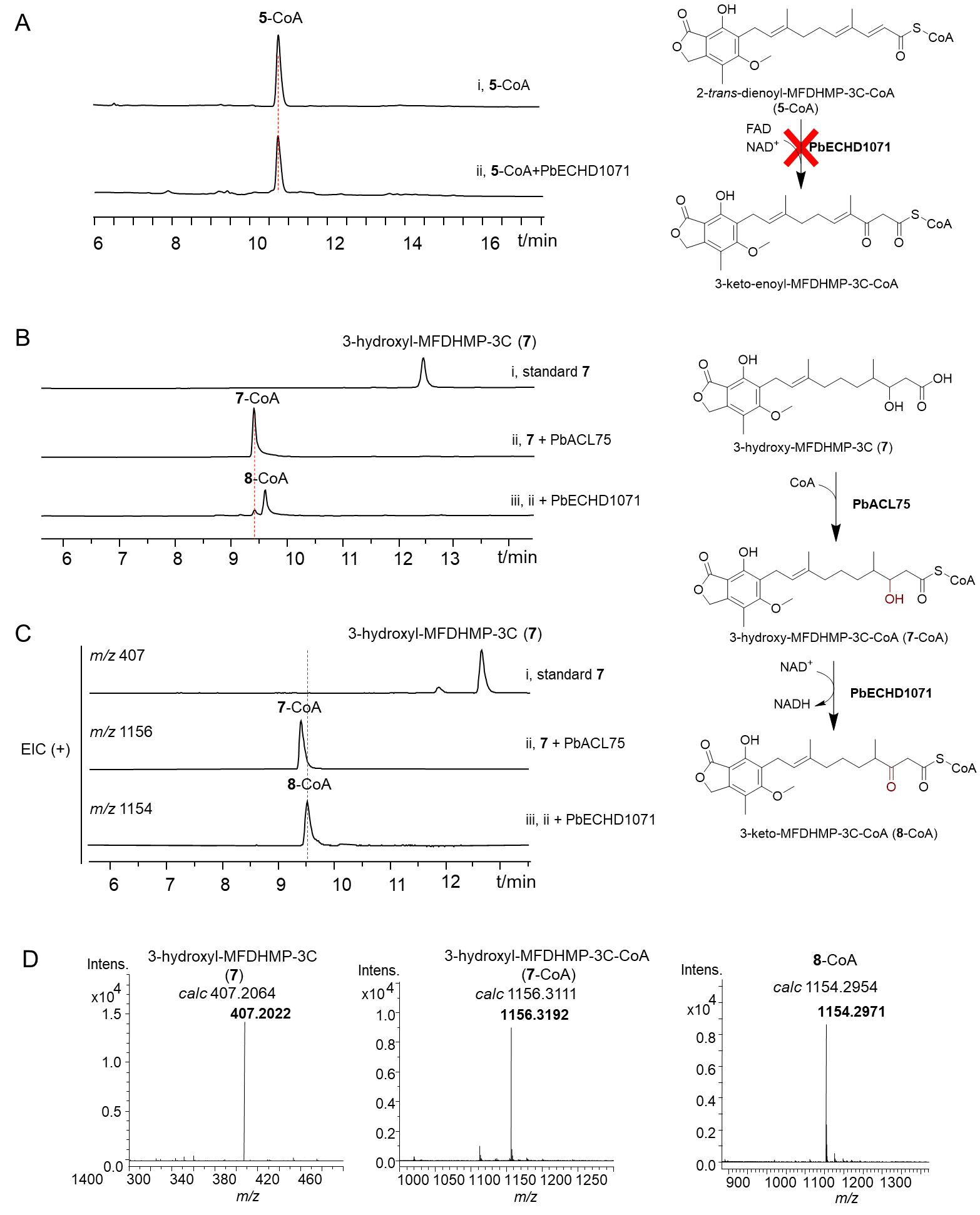


**Figure S21.** LC-HRMS analysis of the *in vitro* conversion from **5**-CoA or **7**-CoA catalyzed by purified PbECHD1071. A), HPLC analysis of the *in vitro* conversion from **5**-CoA catalyzed by purified PbECHD1071. (i) The standard of **5**-CoA; (ii) The reaction of **5**-CoA with PbECHD1071. B), HPLC analysis of the *in vitro* conversion from **7** to **8**-CoA catalyzed by purified PbACL75 and PbECHD1071. (i) The standard of **7**; (ii) The reaction of **7** with PbACL75; (iii) The reaction of **7**-CoA with PbECHD1071. C)**,** (i) The standard of **7**; (ii) The EIC of **7**-CoA in the reaction **7** with PbACL75; (iii) The EIC of **8**-CoA in the reaction **7**-CoA with PbECHD1071. D), The *m/z* values of **8**-CoA, [M+H]^+^= 1154.2971 (*obs*.), *calc.* 1154.2954; **7** [M+H]^+^= 407.2022 (*obs*.), *calc.* 407.2064; **7**-CoA, [M+H]^+^= 1156.3192 (*obs*.), *calc.* 1156.3111.


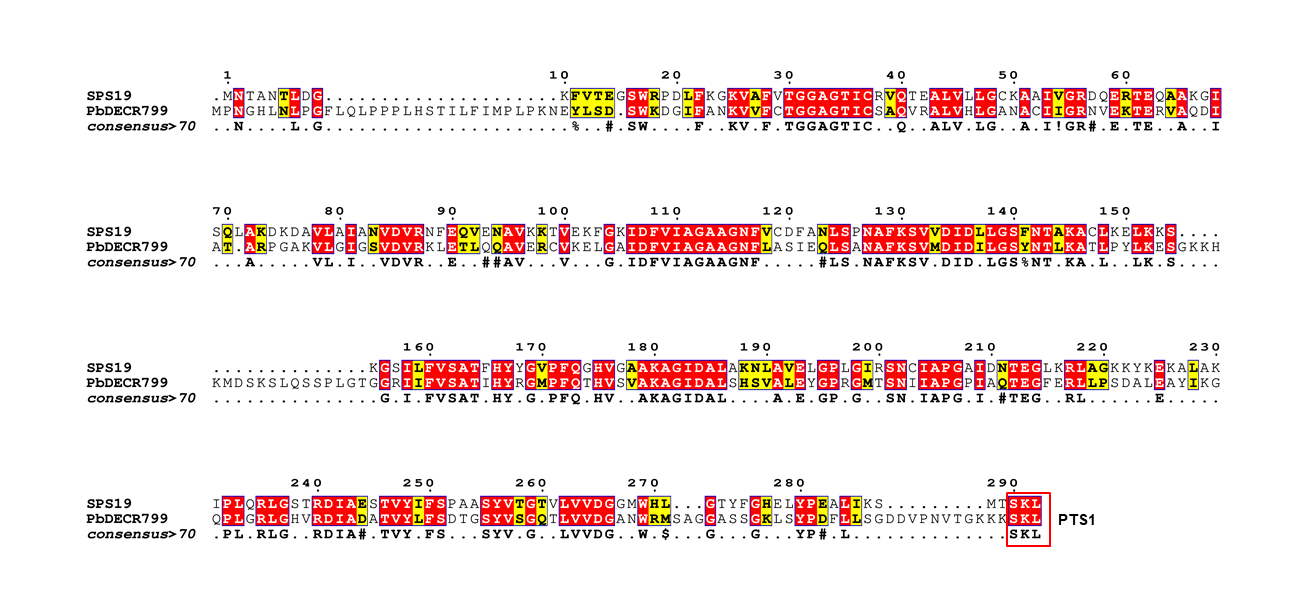


**Figure S22.** Protein sequence alignment of PbDECR799 from *Pb*864 with SPS19 (Gene ID: 855518) from *S. cerevisiae* S288C^[15,16]^. (Red boxes: PTS1-Type I peroxisome target signal)


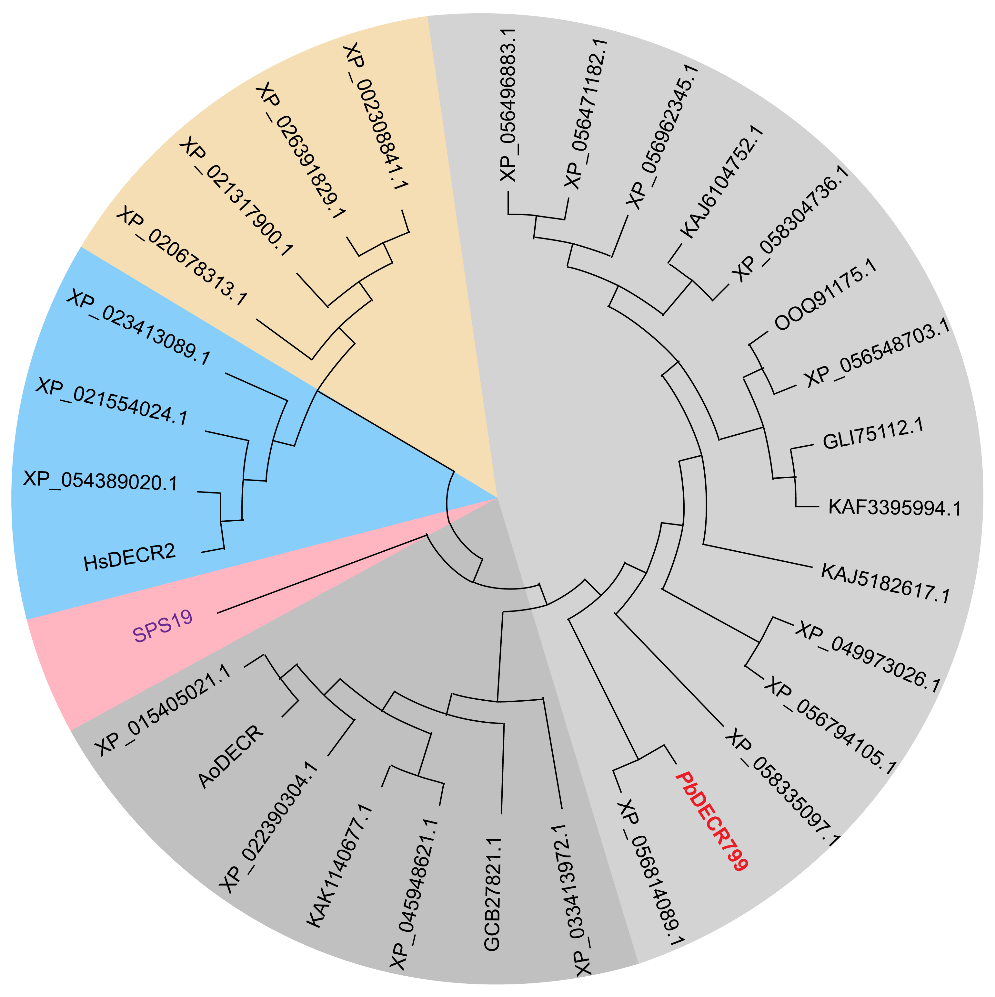


**Figure S23.** The phylogenetic tree of the representative 2,4-dienoyl-CoA reductase. The following sequences were obtained by comparative search in the NCBI database using PbDECR799 (from *Pb*864) as probe. XP_056814089.1, from *Penicillium brevicompactum*, uncharacterized protein; XP_033413972.1, from *Aspergillus lentulus*, peroxisomal 2,4-dienoyl-CoA reductase SPS19; GCB27821.1, from *Aspergillus awamori*, peroxisomal 2,4-dienoyl-CoA reductase SPS19; XP_045948621.1, from *Aspergillus melleus*, peroxisomal 2 4-dienoyl-CoA reductase sps19; KAK1140677.1, from *Aspergillus melleus,* peroxisomal 2 4-dienoyl-CoA reductase sps19; XP_022390304.1, from *Aspergillus bombycis*, peroxisomal 2 4-dienoyl-CoA reductase; AoDECR, from *Aspergillus oryzae*, peroxisomal 2 4-dienoyl-CoA reductase; XP_015405021.1, from *Aspergillus nomiae* NRRL 13137, peroxisomal 2,4-dienoyl-CoA reductase SPS19; SPS19, from from *S. cerevisiae* S288C, peroxisomal 2 4-dienoyl-CoA reductase; HsDECR2, from from *Homo sapiens*, peroxisomal 2 4-dienoyl-CoA reductase; XP_054389020.1, from *Sumatran orangutan*, 2 4-dienoyl-CoA reductase 2; XP_021554024.1, from *Hawaiian monk seal*, 2 4-dienoyl-CoA reductase 2; XP_023413089.1, from *African savanna elephant*, 2 4-dienoyl-CoA reductase; XP_020678313.1, from *Dendrobium catenatum*, peroxisomal 2,4-dienoyl-CoA reductase; XP_021317900.1, from *sorghum*, 2,4-dienoyl-CoA reductase; XP_026391829.1, from *opium poppy*, 2,4-dienoyl-CoA reductase; XP_002308841.1, from *black cottonwood*, 2,4-dienoyl-CoA reductase; XP_056496883.1, from *Penicillium citrinum*, 2,4-dienoyl-CoA reductase; XP_056471182.1, from *Penicillium argentinense*, Peroxisomal 2-4-dienoyl-CoA reductase; XP_056962345.1, from *Penicillium manginii*, 2-4-dienoyl-CoA reductase; KAJ6104752.1, from *Penicillium* sp. IBT 18751x, 2-4-dienoyl-CoA reductase; XP_058304736.1, from *Penicillium cinerascens*, 2-4-dienoyl-CoA reductase; OOQ91175.1, from *Penicillium brasilianum*, 2,4-dienoyl-CoA reductase; XP_056548703.1, from *Penicillium canariense*, Peroxisomal 2-4-dienoyl-CoA reductase; GLI75112.1, from *Penicillium ochrochloron*, 2-4-dienoyl-CoA reductase; KAF3395994.1, from *Penicillium rolfsii*, 2-4-dienoyl-CoA reductase; KAJ5182617.1, from *Penicillium capsulatum*, 2-4-dienoyl-CoA reductase; XP_049973026.1, from *Penicillium oxalicum*, 2-4-dienoyl-CoA reductase; XP_056794105.1, from *Penicillium diatomitis*, 2-4-dienoyl-CoA reductase; XP_058335097.1, from *Penicillium chermesinum*, 2-4-dienoyl-CoA reductase. (lightgrey: represents *Penicillium*; silver: represents *Aspergillus*; lighpink: represents yeast; skyblue: represents animal; wheat: represents plant)


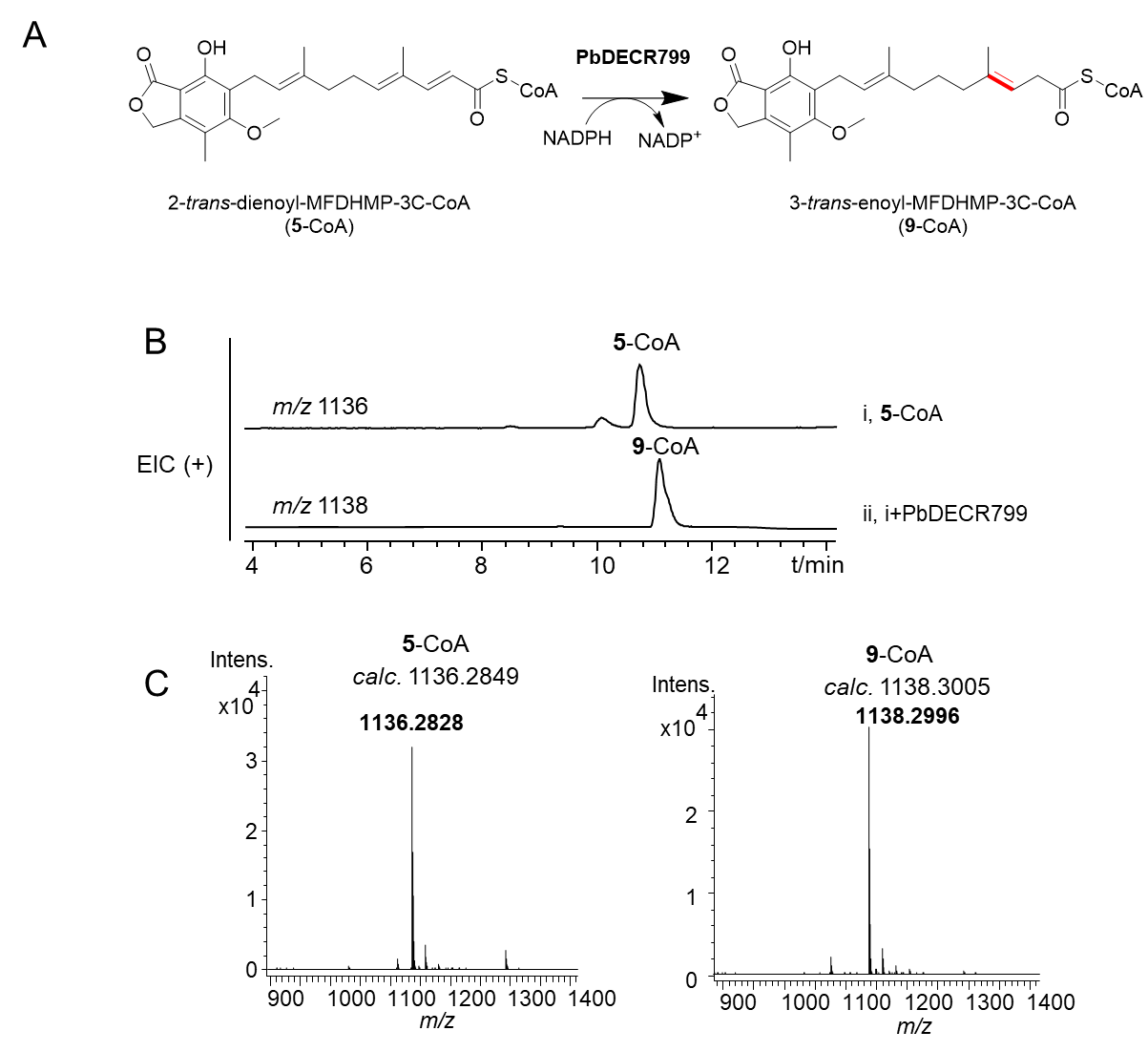


**Figure S24.** LC-HRMS analysis of the *in vitro* conversion from **5**-CoA to **9**-CoA catalyzed by purified PbDECR799. A), The reaction schematic from **5**-CoA to **9**-CoA. B), (i) The EICs of **5**-CoA in the control reaction with boiled PbDECR799; (ii) The EIC of **9**-CoA in the reaction with PbDECR799. C), The *m/z* values of **5**-CoA, [M+H]^+^= 1136.2828 (*obs*.), *calc*. 1136.2849; **9**-CoA, [M+H]^+^= 1138.2996 (*obs*.), *calc*. 1138.3005.


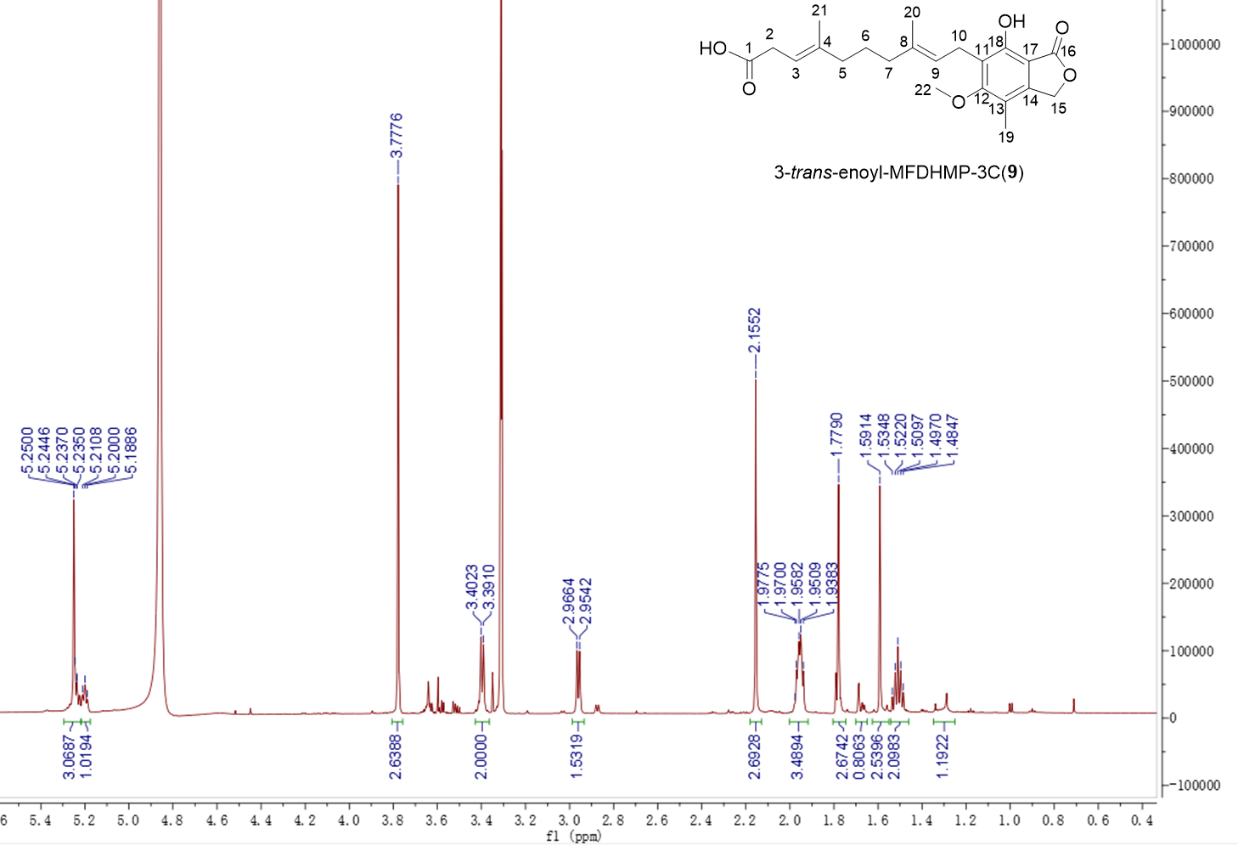


**Figure S25.** ^1^H NMR spectrum of 3-*trans*-enoyl-MFDHMP-3C (**9**) in CD_3_OD (600 MHz).


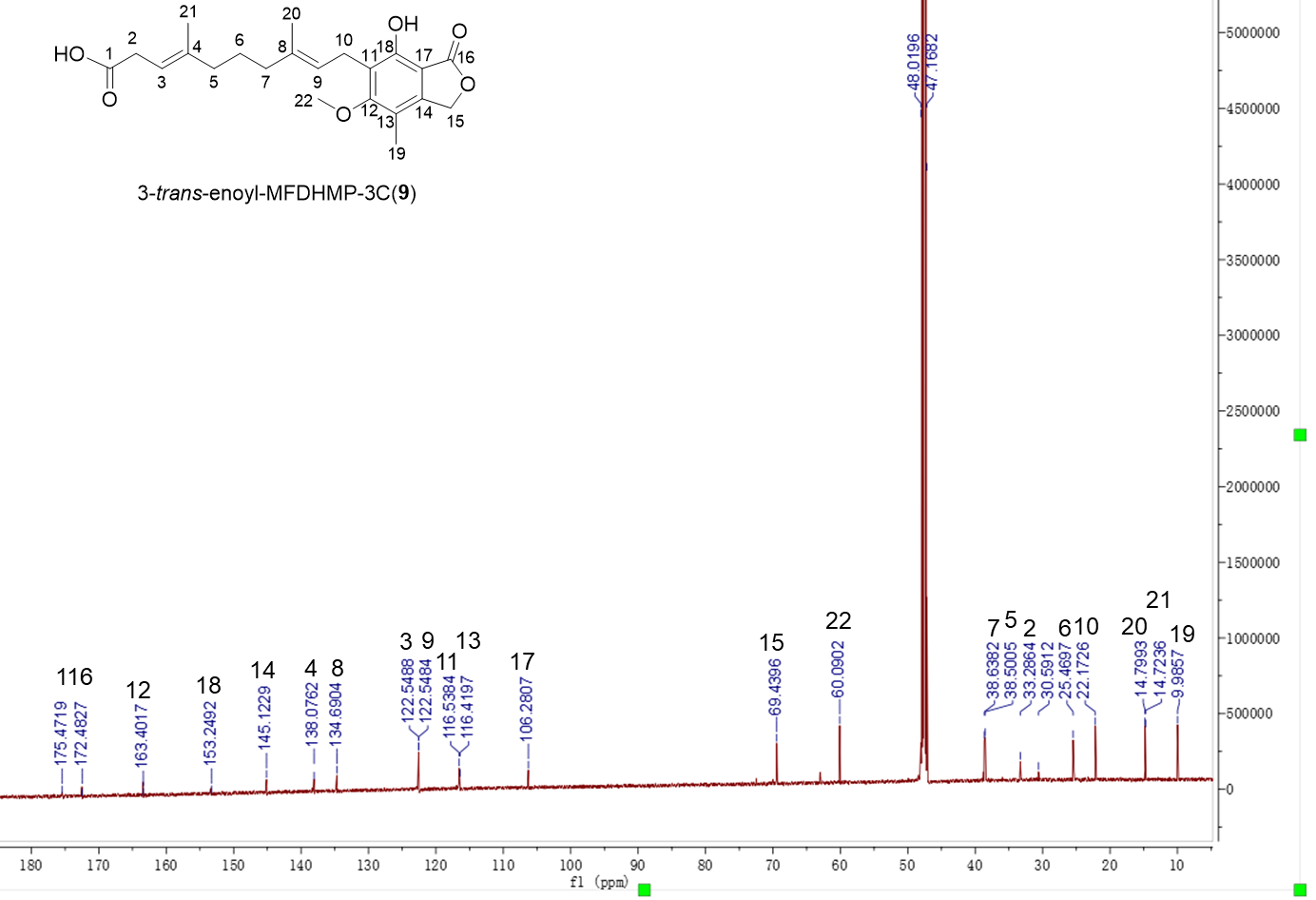


**Figure S26.** ^13^C NMR spectrum of 3-*trans*-enoyl-MFDHMP-3C (**9**) in CD_3_OD (150 MHz).


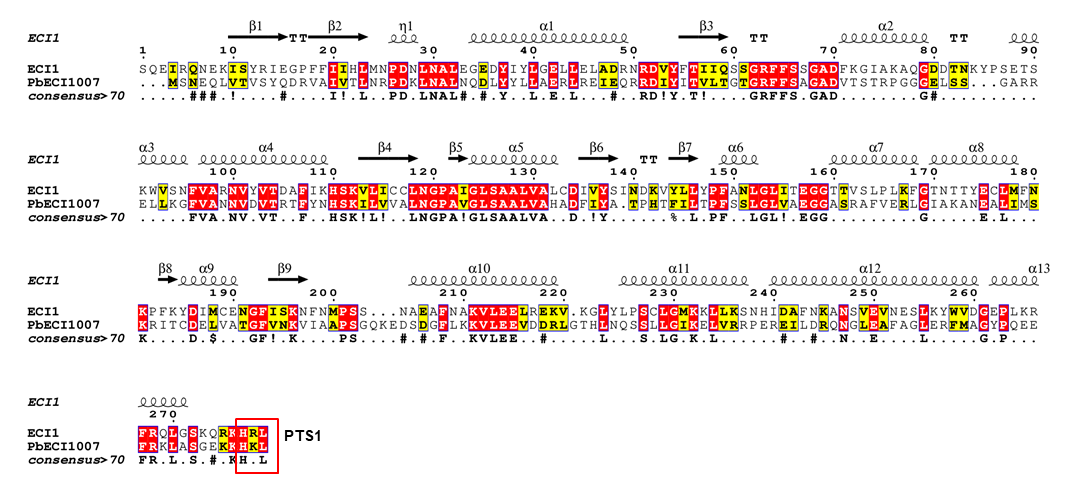


**Figure S27.** Protein sequence alignment of PbECI1007 from *Pb*864 with ECI1 (Gene ID: 850990) from *S. cerevisiae* S288C^[17,18]^ (Red boxes: PTS1-Type I peroxisome target signal).


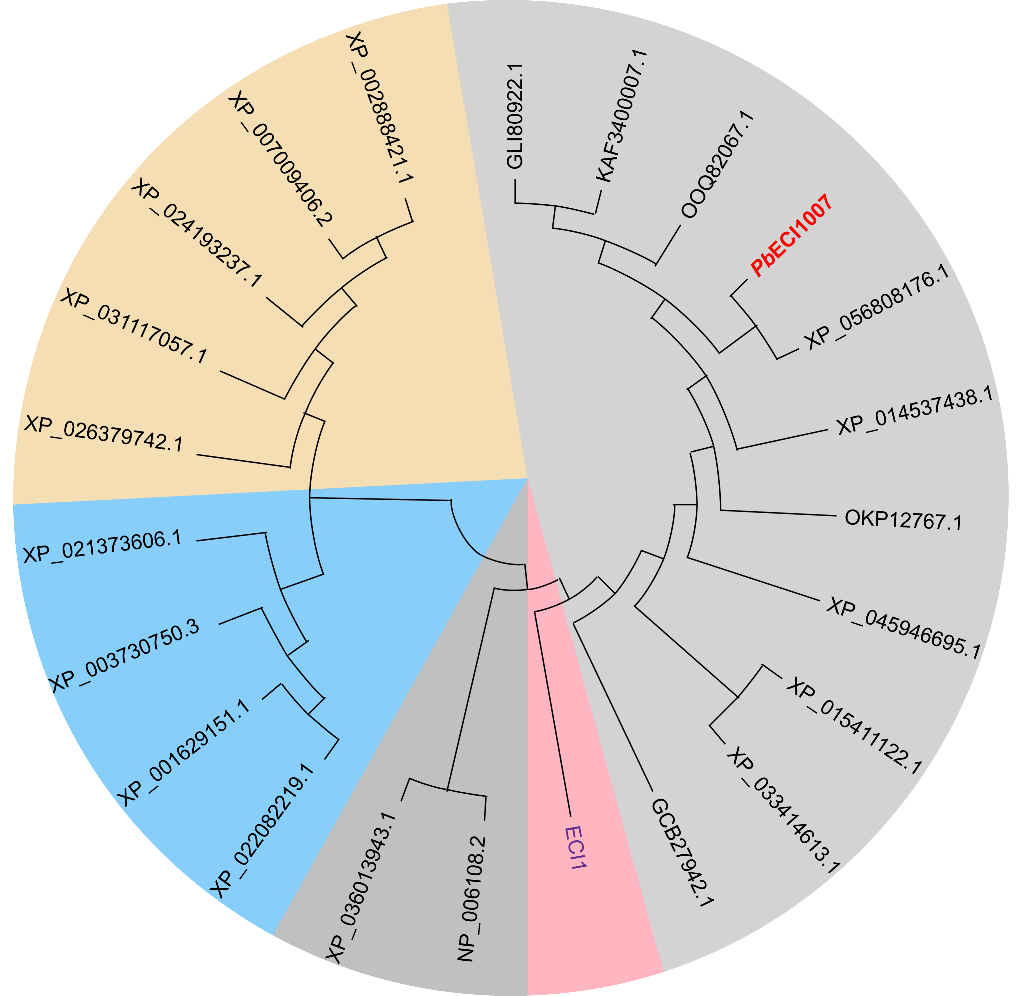


**Figure S28.** The phylogenetic tree of the representative enoyl-CoA isomerase. The following sequences were obtained by comparative search in the NCBI database using PbECI1007 (from *Pb*864) as probe. XP_056808176.1, from *Penicillium brevicompactum*, D3,D2-enoyl-CoA isomerase; XP_014537438.1, from *Penicillium digitatum*, D3,D2-enoyl-CoA isomerase; OKP12767.1, from *Penicillium subrubescens,* 3,2-trans-enoyl-CoA isomerase; XP_045946695.1, from *Aspergillus melleus*, dodecenoyl-CoA isomerase; XP_015411122.1, from *Aspergillus nomiae* NRRL 13137*,* D3,D2-enoyl-CoA isomerase; XP_033414613.1, from *Aspergillus lentulus*, 3,2-trans-enoyl-CoA isomerase; GCB27942.1, from *Aspergillus awamori*, 3,2-trans-enoyl-CoA isomerase; ECI1, from *S. cerevisiae* S288C, 3,2-trans-enoyl-CoA isomerase; NP_006108.2, from *Homo sapiens*, enoyl-CoA delta isomerase 2; XP_036013943.1, from *Mus musculus*, enoyl-CoA delta isomerase 2; XP_022082219.1, from *Acanthaster planci*, enoyl-CoA delta isomerase 2; XP_001629151.1, from *Nematostella vectensis*, enoyl-CoA delta isomerase 1; XP_003730750.3, from *Strongylocentrotus purpuratus*, enoyl-CoA delta isomerase 2; XP_021373606.1, from *Mizuhopecten yessoensis*, enoyl-CoA delta isomerase 1; XP_026379742.1, from *Papaver somniferum*, enoyl-CoA delta isomerase 2; XP_031117057.1, from *Ipomoea triloba*, enoyl-CoA delta isomerase 1; XP_024193237.1, from *Rosa chinensis*, enoyl-CoA delta isomerase 1; XP_007009406.2, from *Theobroma cacao*, enoyl-CoA delta isomerase 1; XP_002888421.1, from *Arabidopsis lyrata subsp. lyrata*, enoyl-CoA delta isomerase 1; GLI80922.1, from *Penicillium ochrochloron*, dodecenoyl-CoA isomerase; KAF3400007.1, from *Penicillium rolfsii*, 3,2-trans-enoyl-CoA isomerase; OOQ82067.1, *Penicillium brasilianum*, peroxisomal D3,D2-enoyl-CoA isomerase. (lightgrey: represents *Penicillium*; silver: represents *Aspergillus*; lighpink: represents yeast; skyblue: represents animal; wheat: represents plant).


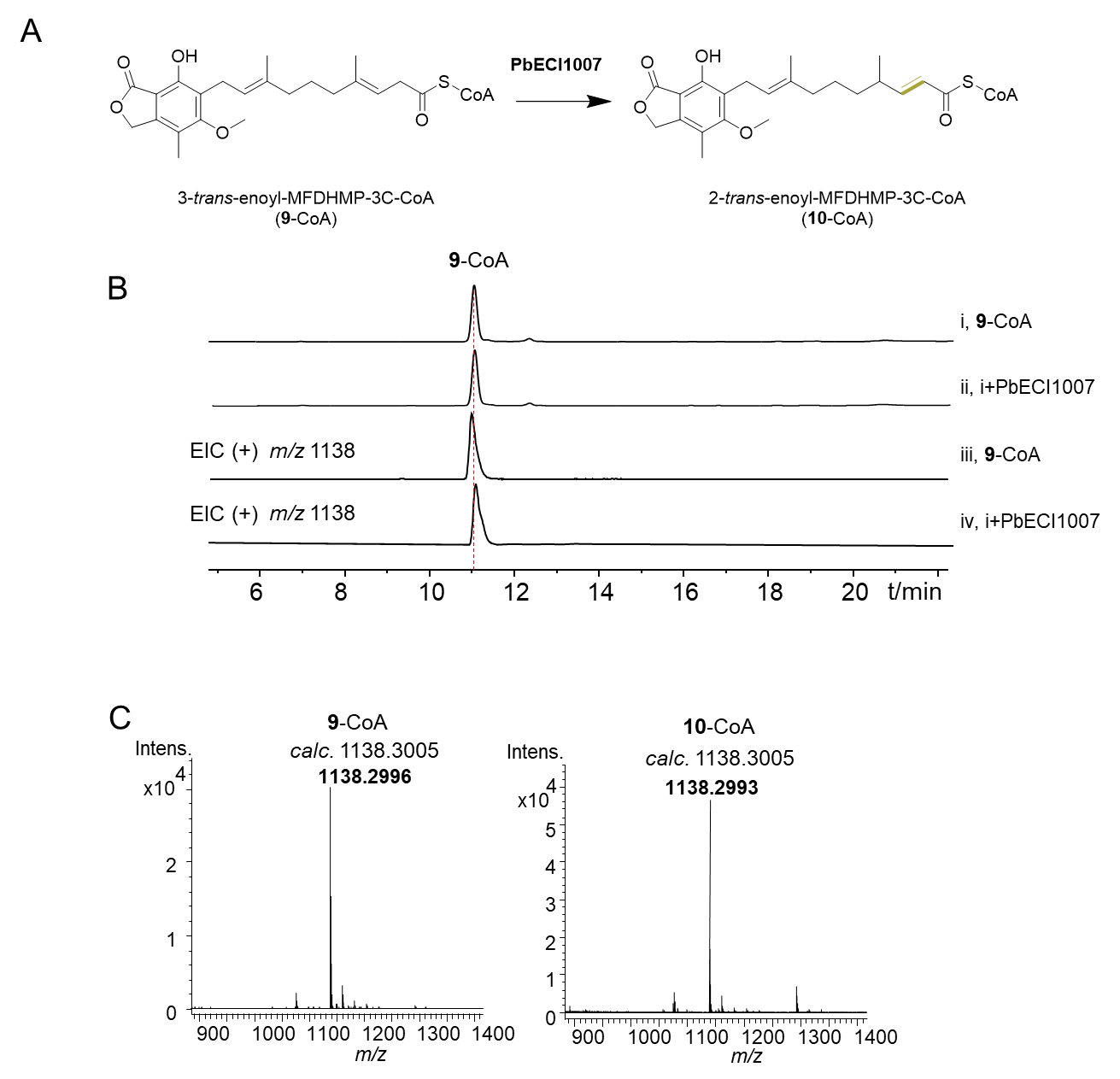


**Figure S29.** LC-HRMS analysis of the *in vitro* conversion from **9**-CoA to **10**-CoA catalyzed by purified PbECI1007. A), The reaction schematic from **9**-CoA to **10**-CoA. B), HPLC and LC-HRMS analysis of the *in vitro* conversion from **9**-CoA to **10**-CoA catalyzed by PbECI1007. (i) HPLC analysis the control reaction of **7**-CoA with boiled PbECI1007; (ii) HPLC analysis the reaction of **9**-CoA with PbECI1007; (iii) The EIC of **10**-CoA in the reaction with boiled PbECI1007; (iv) The EIC of **10**-CoA in the reaction with PbECI1007. C), The *m/z* values of **9**-CoA, [M+H]^+^= 1138.2996 (*obs*.), *calc*. 1138.3005; **10**-CoA, [M+H]^+^= 1138.2993 (*obs*.), *calc*. 1138.3005.


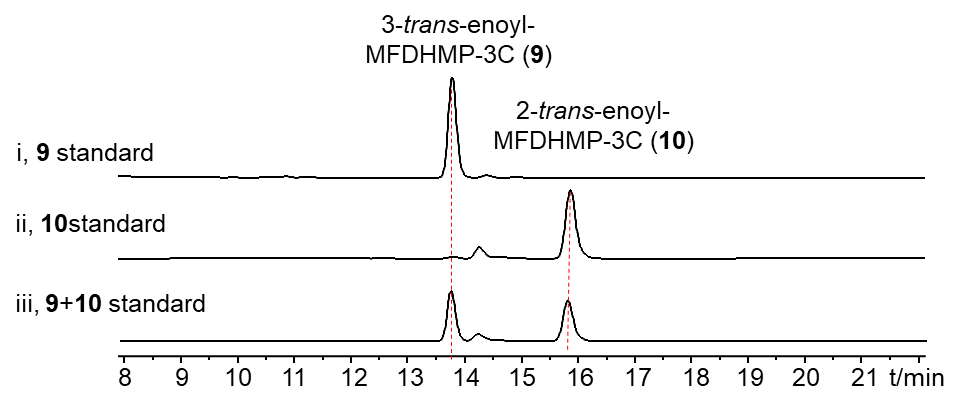


**Figure S30.** Chiral HPLC analysis of **9** and **10**. (i) The standard of **9**; (ii) The standard of **10**; (iii) The co-injection analysis of mixed standards of **9** and **10**.


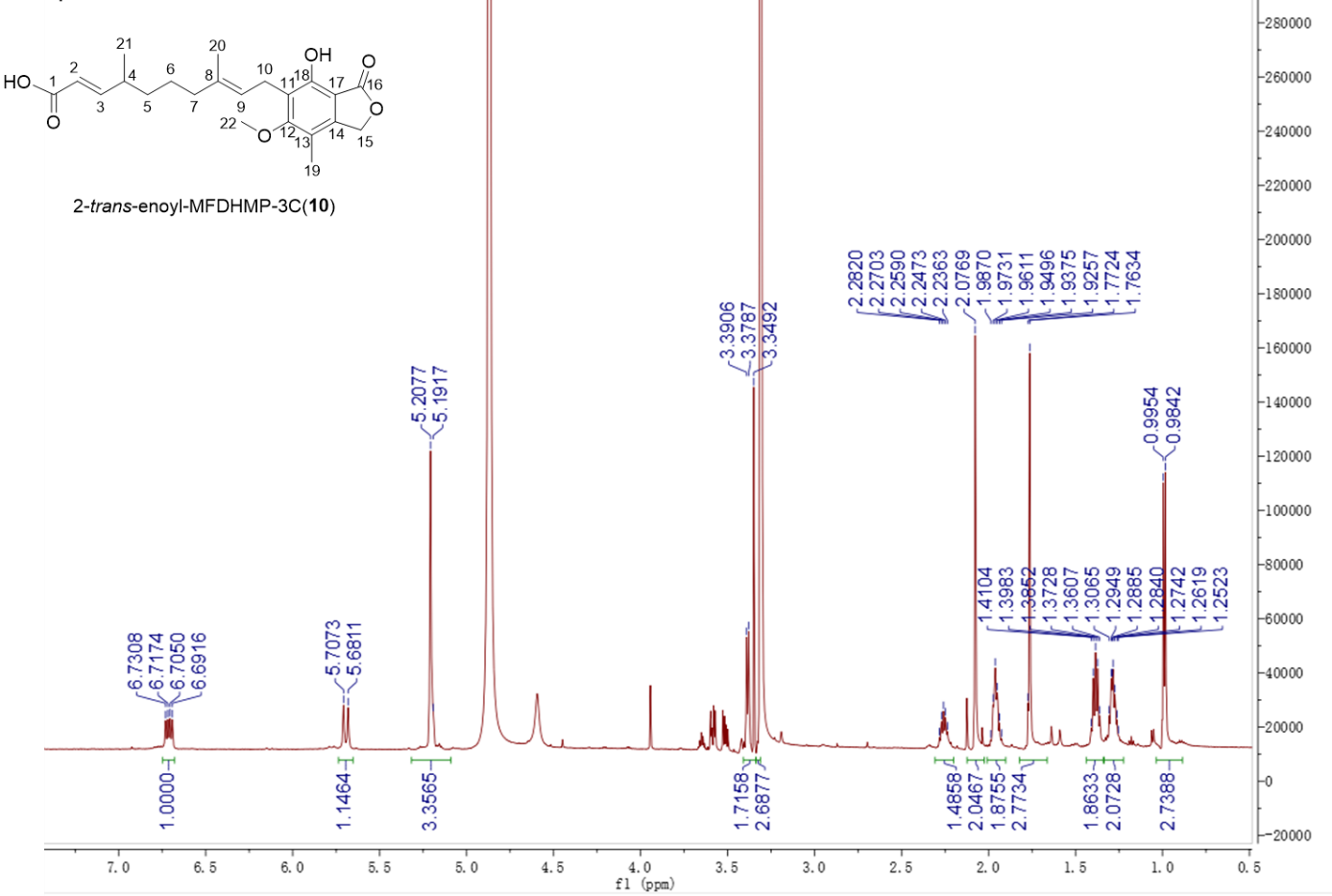


**Figure S31.** ^1^H NMR spectrum of 2-*trans*-enoyl-MFDHMP-3C (**10**) in CD_3_OD (600 MHz).


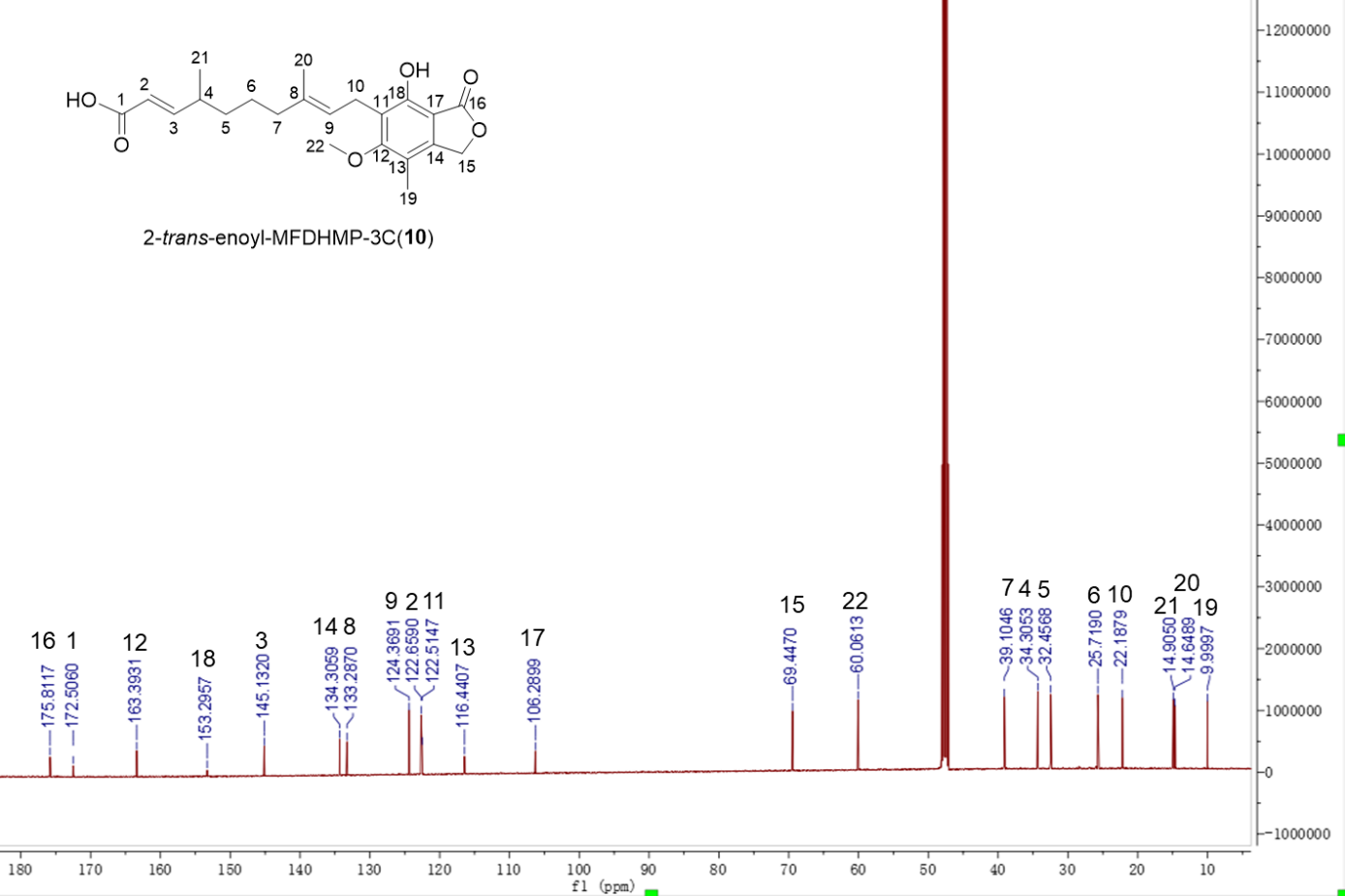


**Figure S32.** ^13^C NMR spectrum of 2-*trans*-enoyl-MFDHMP-3C (**10**) in CD_3_OD (150 MHz).


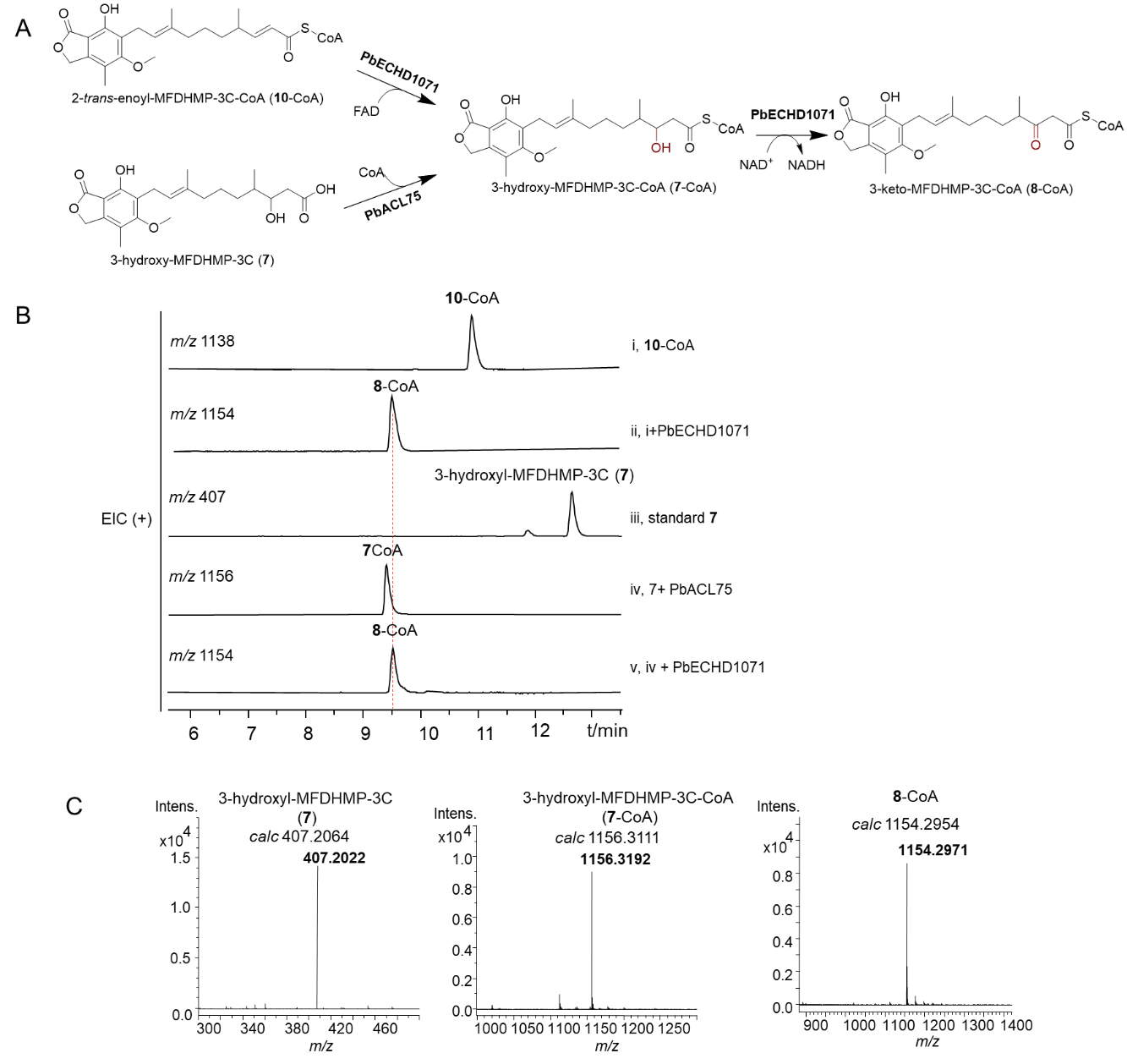


**Figure S33.** LC-HRMS analysis of the *in vitro* conversion from 2-*trans*-enoyl-MFDHMP-3C-CoA (**10**-CoA) to 3-keto-MFDHMP-3C-CoA (**8**-CoA) catalyzed by purified PbECHD1071. A), The reaction schematic from **10**-CoA to **8**-CoA. B), (i) The EIC of **10**-CoA in the control reaction with boiled PbECHD1071; (ii) The EIC of **8**-CoA in the reaction with PbECHD1071; (iii) The EIC of standard **7**; (iv) The EIC of **7**-CoA in the reaction with PbACL75; (v) The EIC of **8**-CoA in the reaction with PbACL75, PbECHD1071. C), the *m/z* values of **8**-CoA, [M+H]^+^= 1154.2971 (*obs*.), *calc.* 1154.2954; **7** [M+H]^+^= 407.2022 (*obs*.), *calc.* 407.2064; **7**-CoA, [M+H]^+^= 1156.3192 (*obs*.), *calc.* 1156.3111.


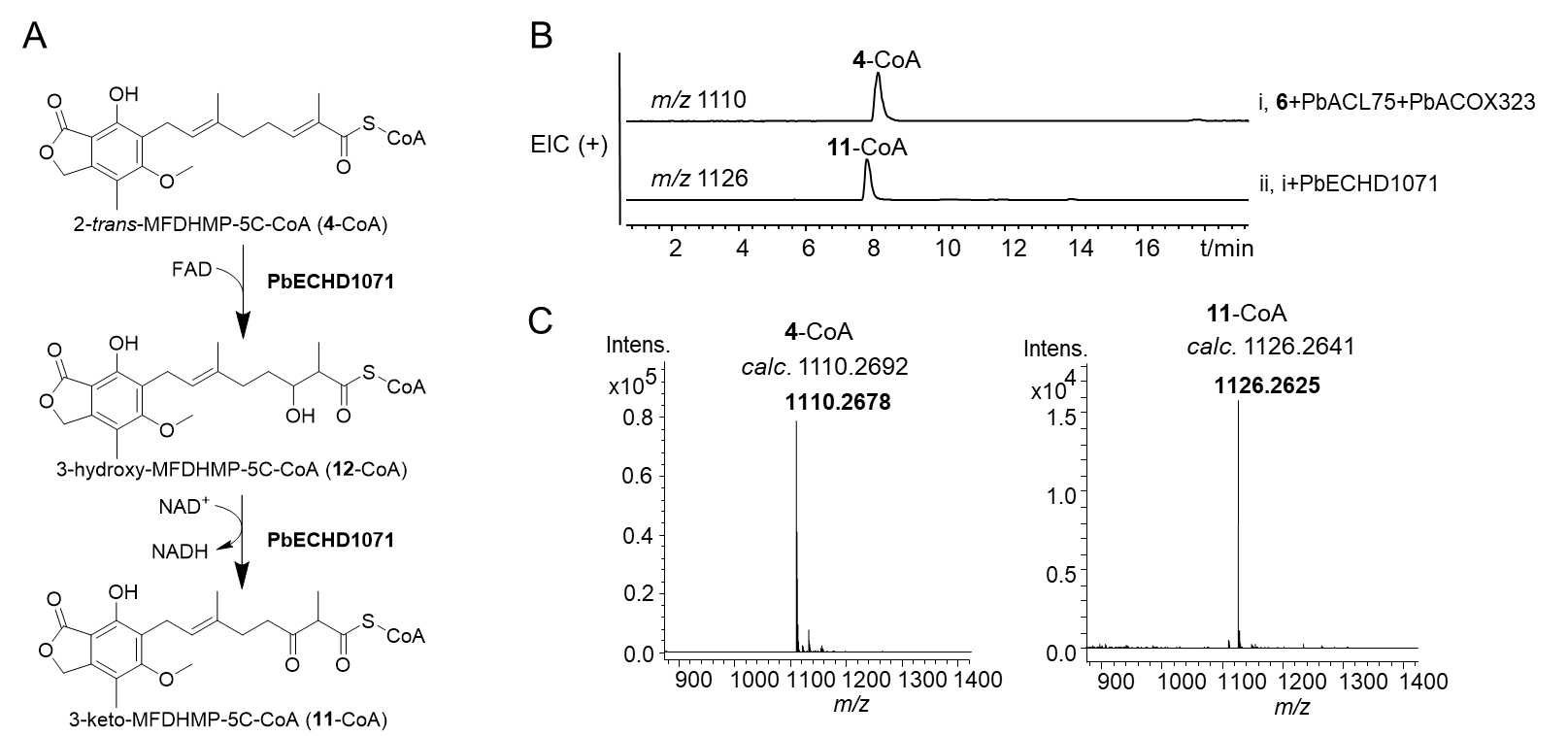


**Figure S34.** LC-HRMS analysis of the *in vitro* conversion from 2-*trans*-MFDHMP-5C-CoA (**4**-CoA) to 3-keto-MFDHMP-5C-CoA (**11**-CoA) catalyzed by purified PbECHD1071. A), The reaction schematic from **4**-CoA to **11**-CoA. B), (i) The EIC of **4**-CoA in the reaction of **6** with PbACL75, PbACOX323 and boiled PbECHD1071; (ii) The EIC of **11**-CoA in the reaction of **6** with PbACL75, PbACOX323, PbECHD1071. C), the *m/z* values of **4**-CoA, [M+H]^+^= 1110.2678 (*obs*.), *calc*. 1110.2692; **11**-CoA, [M+H]^+^= 1126.2625 (*obs*.), *calc.* 1126.2641.


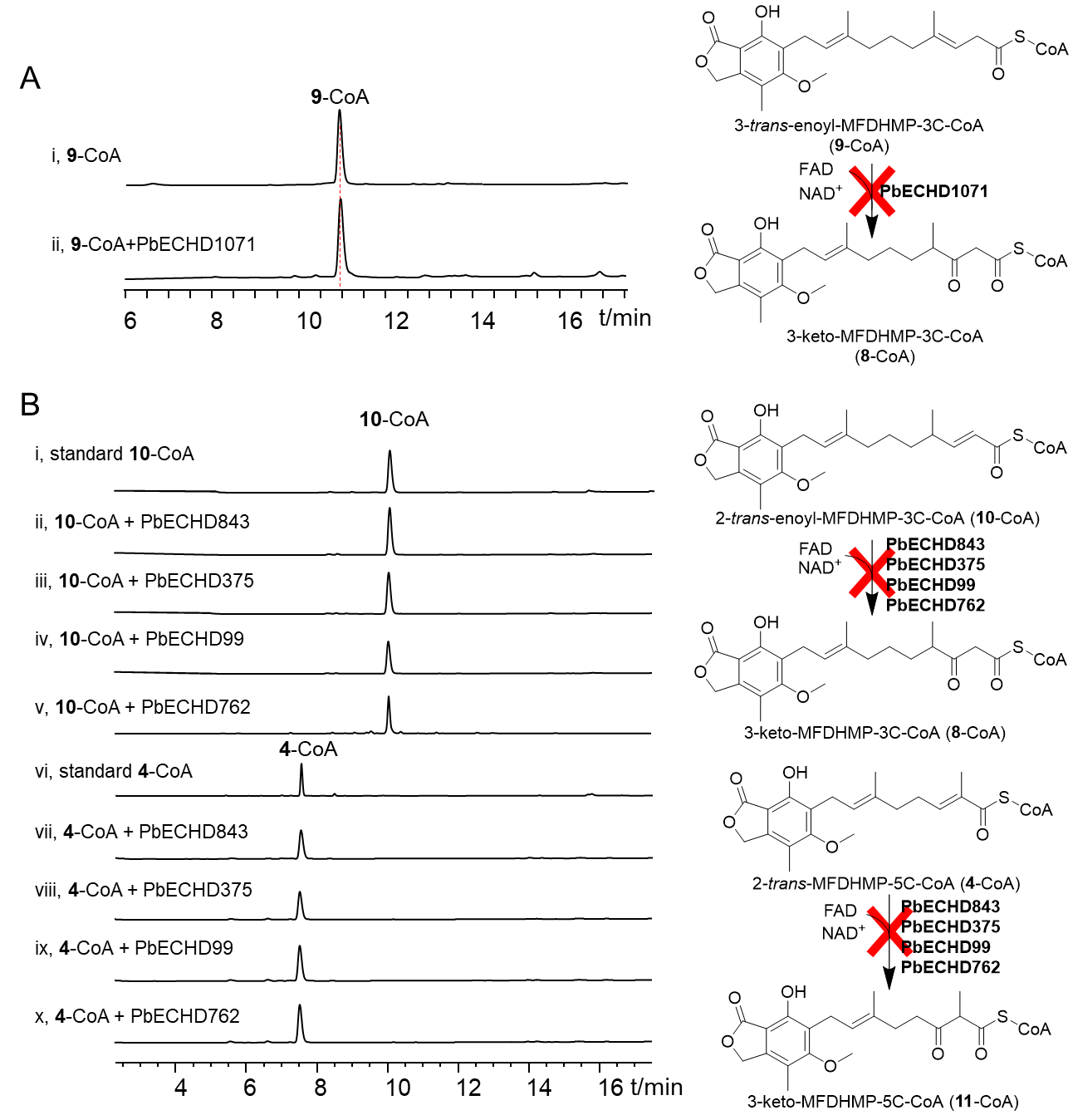


**Figure S35.** A), HPLC analysis the reaction of PbECHD1071 with **7**-CoA. (i) Standard of **7**-CoA; (ii) The reaction of **7**-CoA with PbECHD1071 in the presence of FAD and NAD^+^. B), HPLC analysis the reaction of PbECHDs with **8**-CoA or **4**-CoA. (i) Standard of **10**-CoA; (ii-v) The reaction of **10-**CoA with PbECHD843, PbECHD375, PbECHD99 and PbECHD762 in the presence of FAD and NAD^+^, respectively; (vi) Standard of **4**-CoA; (vii-x) The reaction of **4**-CoA with PbECHD843, PbECHD375, PbECHD99 and PbECHD762 in the presence of FAD and NAD^+^, respectively.


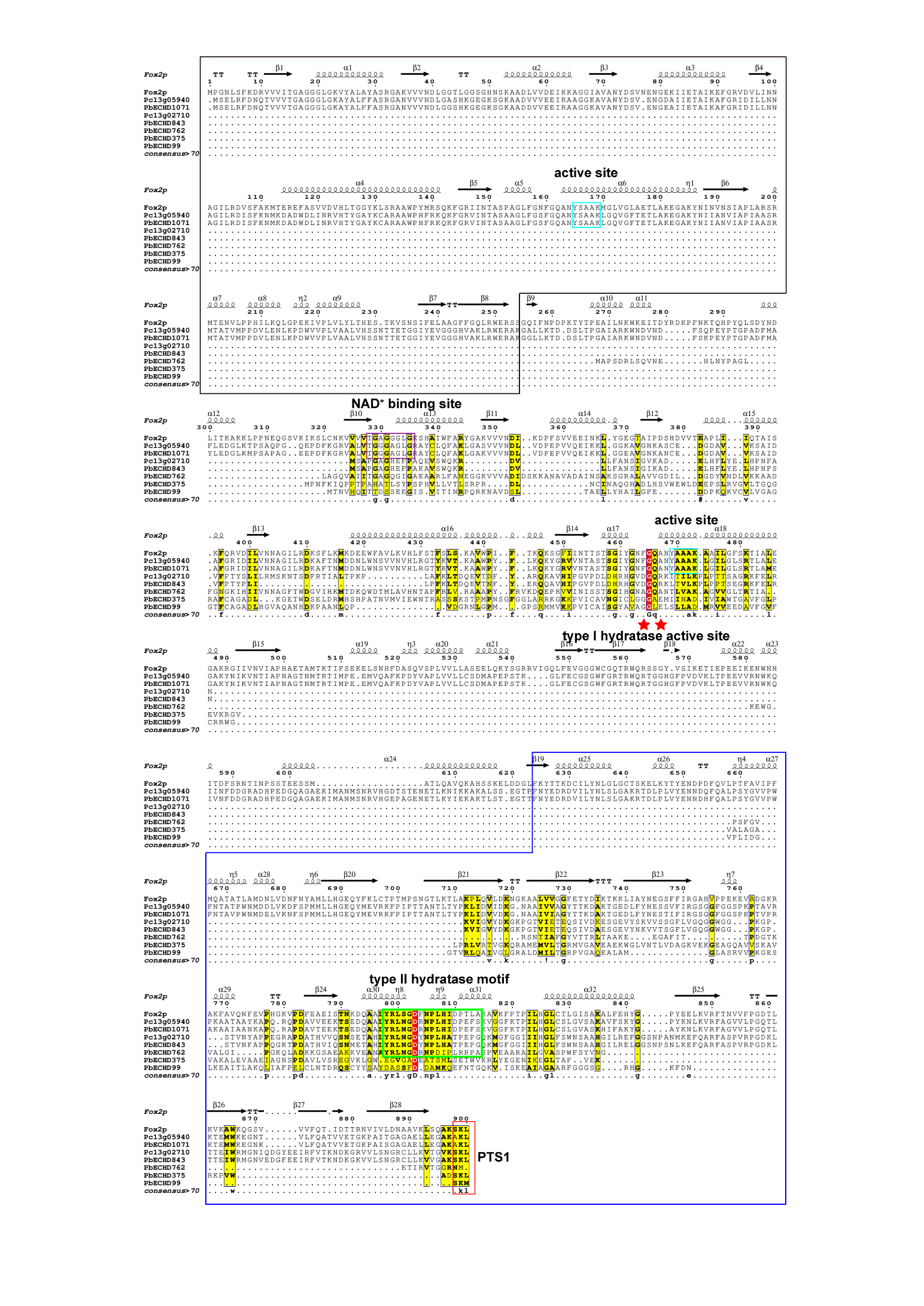


**Figure S36.** Protein sequence alignment of PbECHD1071 PbECHD375, PbECHD762, PbECHD843 and PbECHD99 from *Pb*864 with Fox2p (Gene ID: 853878) from *S. cerevisiae* S288C together with Pc13g05940 and Pc13g02710 from *P. chrysogenum*^[17,19-21]^. Pfam analysis the conserved domains of PbECHD1071 contains two dehydrogenase domains (*N*-terminal) and a type II hydratase domain (*C*-terminal). The PbECHD762 and PbECHD843 only contains the type II hydratase motif [Y/F]-X_1,2_-[L/V/I/G]-[S/T/G/C]-G-D-X-N-P-[L/I/V]-H-X_5_-[A/S]^[20]^ and belongs to type II hydratase. The PbECHD375 and PbECHD99 contains the type I hydratase active site and belongs to type II hydratase. The dehydrogenase domain is highlighted in black boxes and the hydratase domain is highlighted in blue boxes. The type II hydratase motif is highlighted in green boxes and the dehydrogenase active site is highlighted in cyan boxes and the NAD^+^ binding site is highlighted in purple boxes. The type I hydratase active site is marked with a red pentagram. PTS1-The Type I Peroxisome target signal is highlighted in red boxes.


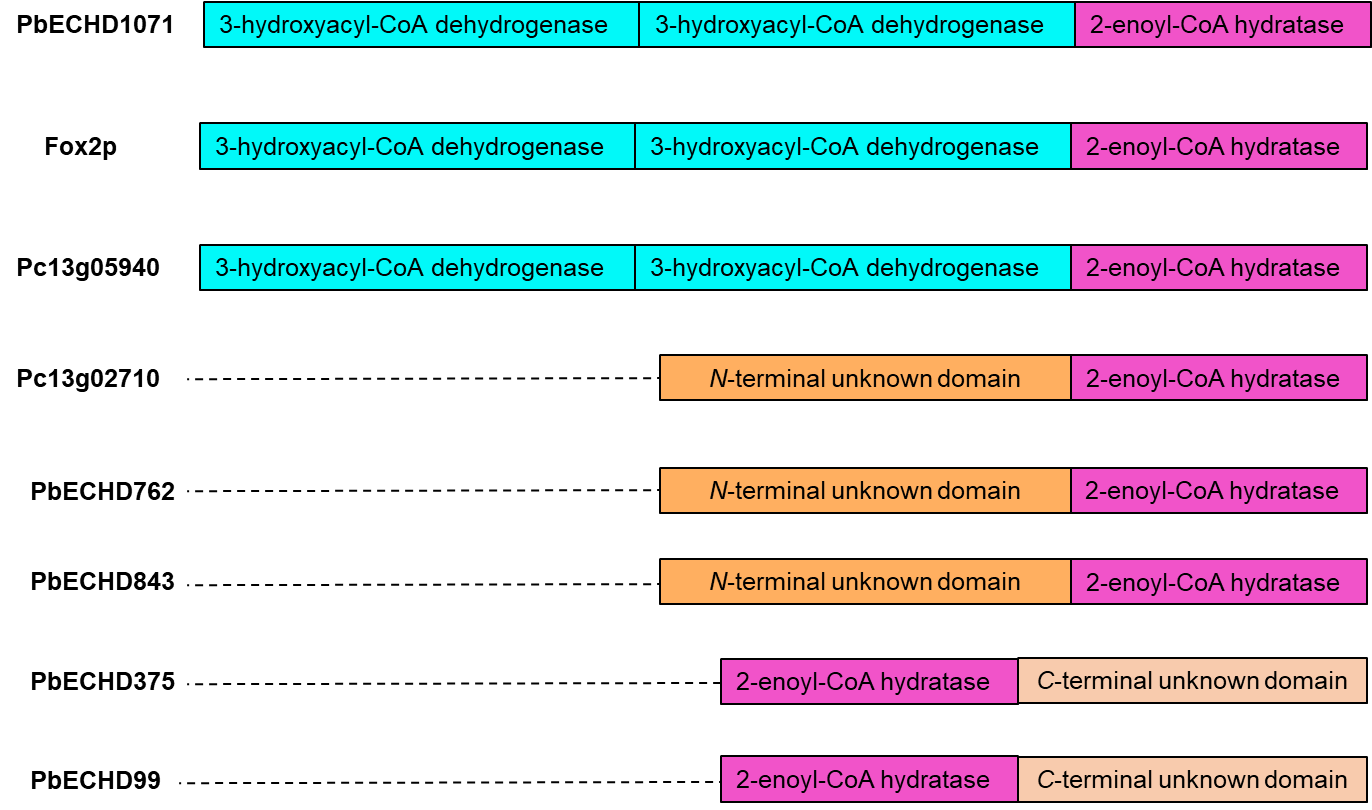


**Figure S37.** Structural analysis of PbECHD1071, Fox2p, Pc13g05940, Pc13g02710, PbECHD762, PbECHD843, PbECHD375 and PbECHD99. PbECHD1071 (from *Pb*864), Fox2p (from *S. cerevisiae* S288C), Pc13g05940 (from *P. chrysogenum*) were contains three domains. Pc13g02710 (from *P. chrysogenum*), PbECHD762, PbECHD843, PbECHD375 and PbECHD99 were contains hydratase domains and one unknown domain.


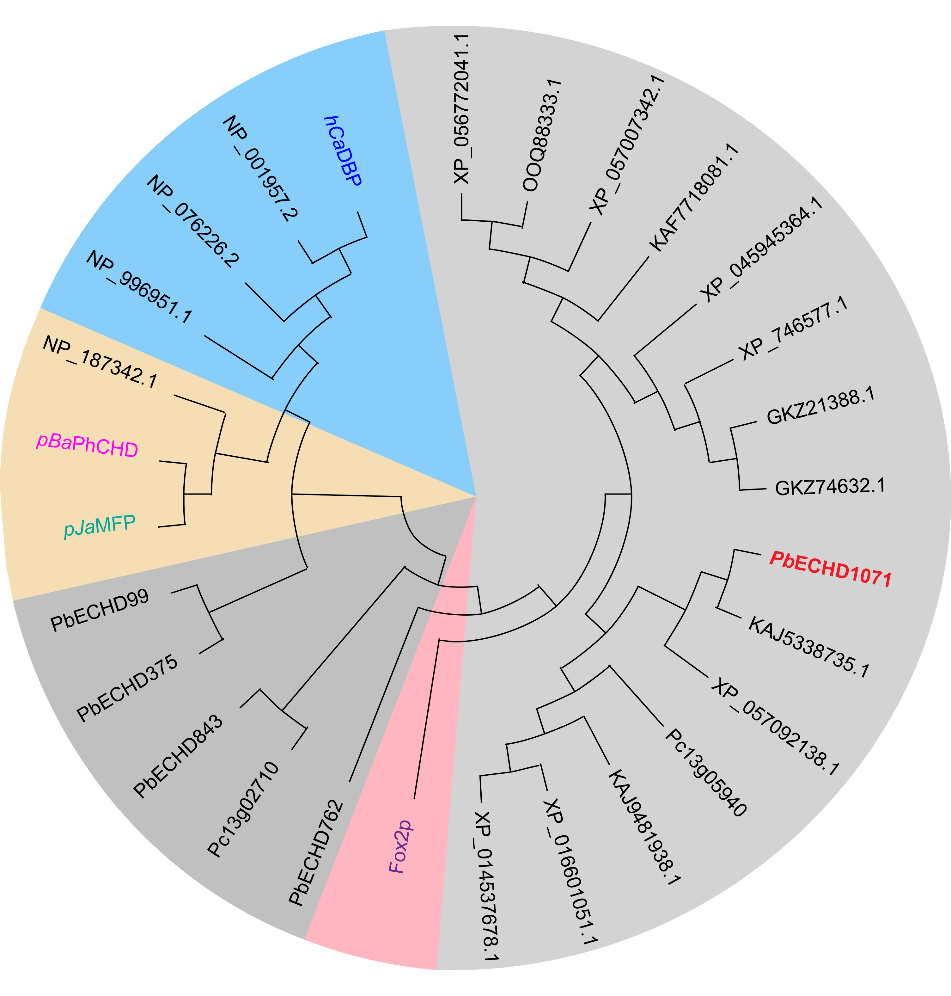


**Figure S38.** The phylogenetic tree of the representative peroxisomal hydratase-dehydrogenase. The following sequences were obtained by comparative search in the NCBI database using PbECHD1071, PbECHD375, PbECHD99, PbECHD843 and PbECHD762 (from *Pb*864) as probes. KAJ5338735.1, from *Penicillium brevicompactum*, hypothetical protein; KAF7718081.1, from *Penicillium ucsense*, Multifunctional Enoyl-CoA hydratase 2/D-3-hydroxyacyl-CoA dehydrogenase; XP_057007342.1, from *Penicillium subrubescens,* Peroxisomal hydratase-dehydrogenase-epimerase; XP_056772041.1, from *Penicillium daleae*, Peroxisomal hydratase-dehydrogenase-epimerase; OOQ88333.1, from *Penicillium brasilianum,* Peroxisomal hydratase-dehydrogenase-epimerase; XP_045945364.1, from *Aspergillus melleus*, bifunctional hydroxyacyl-CoA dehydrogenase/enoyl-CoA hydratase fox2; XP_746577.1, from *Aspergillus fumigatus* Af293, peroxisomal multifunctional beta-oxidation protein (MFP); GKZ21388.1, from *Aspergillus brasiliensis*, bifunctional hydroxyacyl-CoA dehydrogenase/enoyl-CoA hydratase fox2; GKZ74632.1, from *Aspergillus niger*, bifunctional hydroxyacyl-CoA dehydrogenase/enoyl-CoA hydratase fox2; Fox2p, from *S. cerevisiae* S288C, Peroxisomal hydratase-dehydrogenase; Pc13g02710, from *Penicillium chrysogenum*, Peroxisomal hydratase-dehydrogenase; *pJa*MFP, from *Arabidopsis thaliana*, Peroxisomal hydratase-dehydrogenase; *pBa*PhCHD, from *Petunia hybrida*, Peroxisomal hydratase-dehydrogenase; NP_187342.1, from *Arabidopsis thaliana*, multifunctional protein 2 (MFP2); NP_996951.1, from *Danio rerio*, peroxisomal bifunctional enzyme; NP_076226.2, from *Mus musculus*, peroxisomal bifunctional enzyme; NP_001957.2, from *Homo sapiens*, peroxisomal bifunctional enzyme; hCaDBP, from *Rattus norvegicus*, peroxisomal bifunctional enzyme; XP_016601051.1, from *Penicillium expansum*, Short-chain dehydrogenase/reductase; XP_014537678.1, from *Penicillium digitatum*, Peroxisomal multifunctional beta-oxidation protein (MFP); KAJ9481938.1, from *Penicillium thymicola*, Peroxisomal hydratase-dehydrogenase-epimerase; Pc13g05940, from *Penicillium chrysogenum*, Peroxisomal hydratase-dehydrogenase; XP_057092138.1, from *Penicillium soppii*, Short-chain dehydrogenase/reductase SDR. (lightgrey: represents *Penicillium*; silver: represents *Aspergillus*; lighpink: represents yeast; skyblue: represents animal; wheat: represents plant).


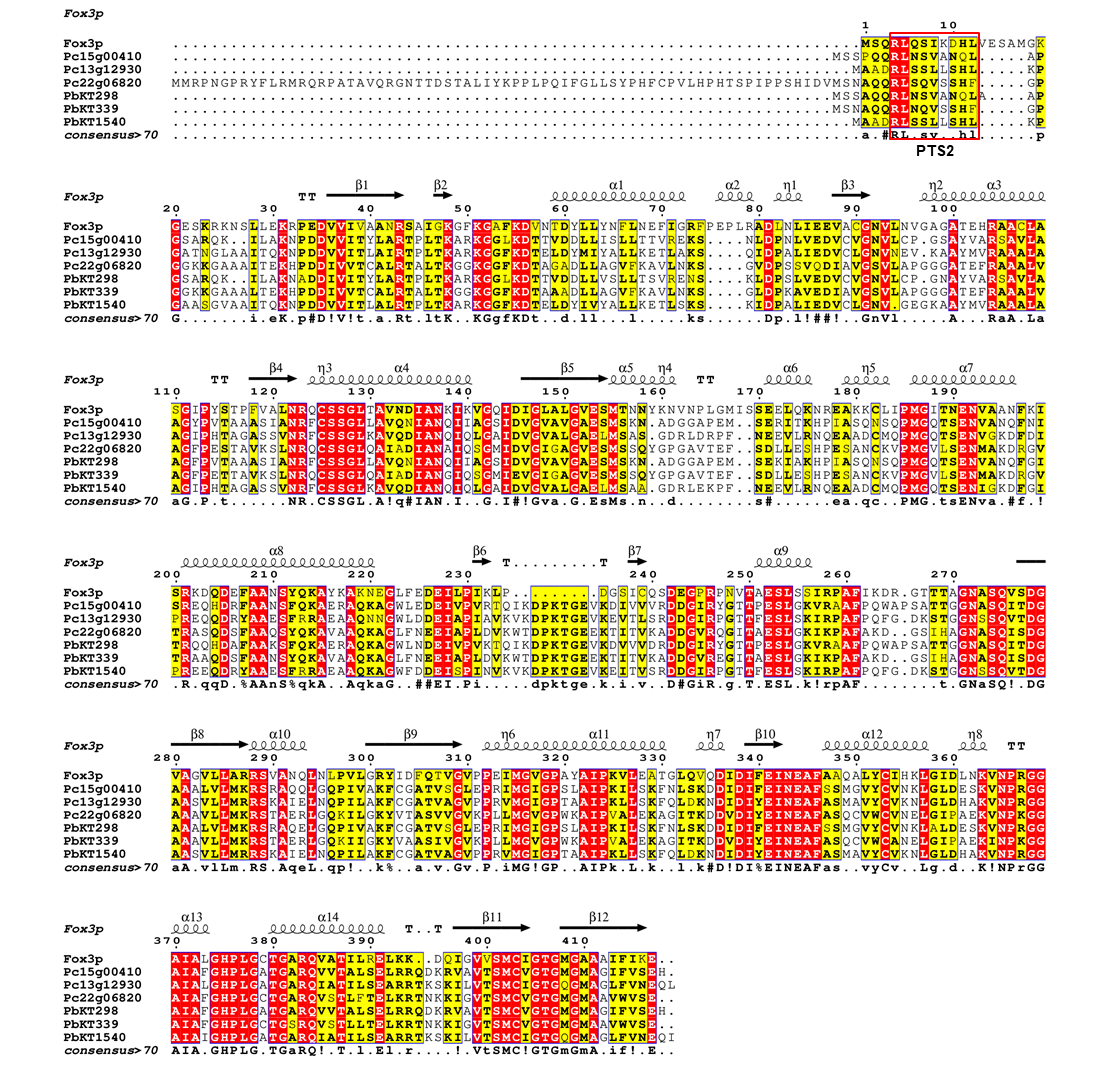


**Figure S39.** Protein sequence alignment of PbKT298, PbKT339, PbKT1540 from *Pb*864 with Fox3p (Gene ID: 854646) from *S. cerevisiae* S288C, and Pc15g00410, Pc22g06820, Pc13g12930 from *P. chrysogenu*^[21,22]^ (Red boxes: Type II Peroxisome target signal)


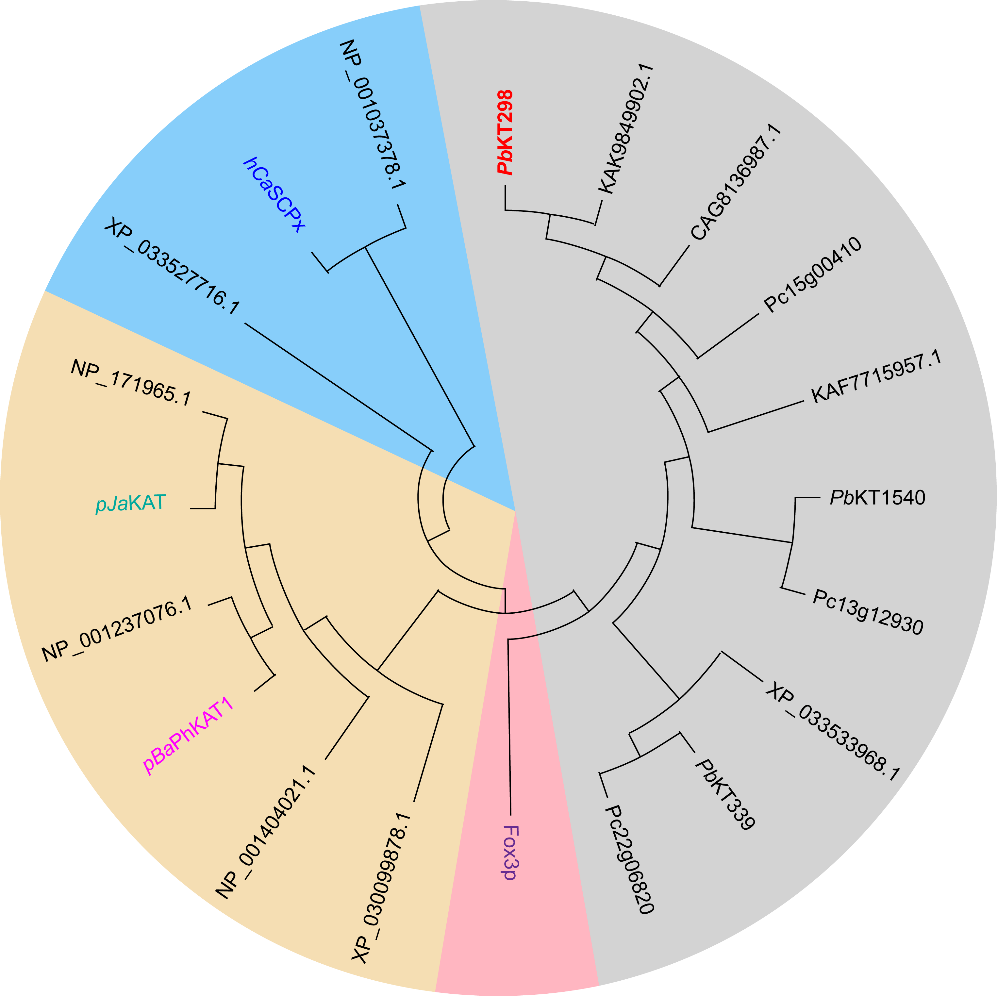


**Figure 40.** The phylogenetic tree of the representative peroxisomal 3-ketoacyl-CoA thiolase. The following sequences were obtained by comparative search in the NCBI database using PbKT298, PbKT339 and PbKT1540 (from *Pb*864) as probes. KAK9849902.1, from *Penicillium brevicompactum*, Thiolase; CAG8136987.1, from *Penicillium salamii*, unnamed protein product; Pc15g00410, from *Penicillium chrysogenum,* Thiolase; KAF7715957.1, from *Penicillium ucsense*, 3-ketoacyl-CoA thiolase; Pc13g12930, from *Penicillium chrysogenum,* Thiolase; XP_033533968.1, from *Eremomyces bilateralis*, 3-ketoacyl-CoA thiolase-like protein; Pc22g06820, from *Penicillium chrysogenum,* Thiolase; XP_030099878.1, from *Papaver somniferum*, 3-ketoacyl-CoA thiolase A; NP_001404021.1, from *Oryza sativa Japonica Group*, 3-ketoacyl-CoA thiolase 2; Fox3p, from *S. cerevisiae* S288C, Thiolase; *pBa*PhKAT1, from *Petunia hybrida*, 3-ketoacyl-CoA thiolase; *pJa*KAT, from *Arabidopsis thaliana*, 3-ketoacyl-CoA thiolase; NP_001237076.1, from *Glycine max*, 3-ketoacyl-CoA thiolase; NP_171965.1, from *Arabidopsis thaliana*, peroxisomal 3-ketoacyl-CoA thiolase 4; XP_033527716.1, from *Dothidotthia symphoricarpi*, 3-ketoacyl-CoA thiolase B; *hCa*SCPx, from *Homo sapiens*, Sterol carrier protein X-related thiolase; NP_001037378.1, from *Bombyx mori*, Sterol carrier protein X-related thiolase. (lightgrey: represents *Penicillium*; lighpink: represents yeast; skyblue: represents animal; wheat: represents plant).


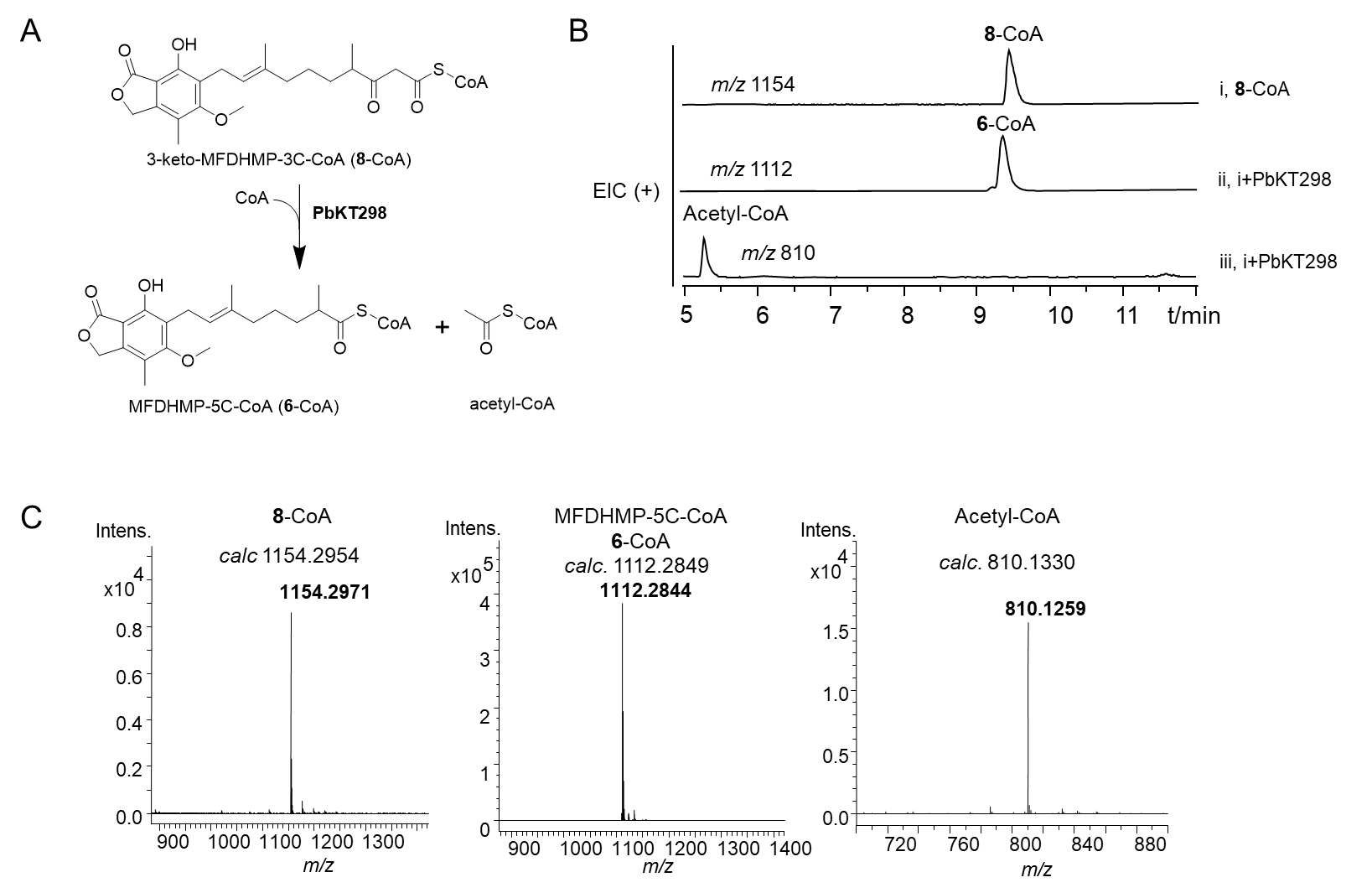


**Figure S41.** LC-HRMS analysis of the *in vitro* conversion from **8**-CoA to **6**-CoA catalyzed by purified PbKT298. A), The reaction schematic from **8**-CoA to **6**-CoA. B), (i) The EIC of **8**-CoA in the control reaction with boiled PbKT298; (ii) The EIC of **6**-CoA in the reaction of PbKT298 with **8**-CoA; (iii) The EIC of acetyl-CoA in the reaction of PbKT298 with **8**-CoA. C), The *m/z* values of **8**-CoA, [M+H]^+^= 1154.2971 (*obs*.), *calc.* 1154.2954; **6**-CoA, [M+H]^+^= 1112.2844 (*obs*.), *calc*. 1112.2849; acetyl-CoA, [M+H]^+^= 810.1259 (*obs*.), *calc*. 810.1330.


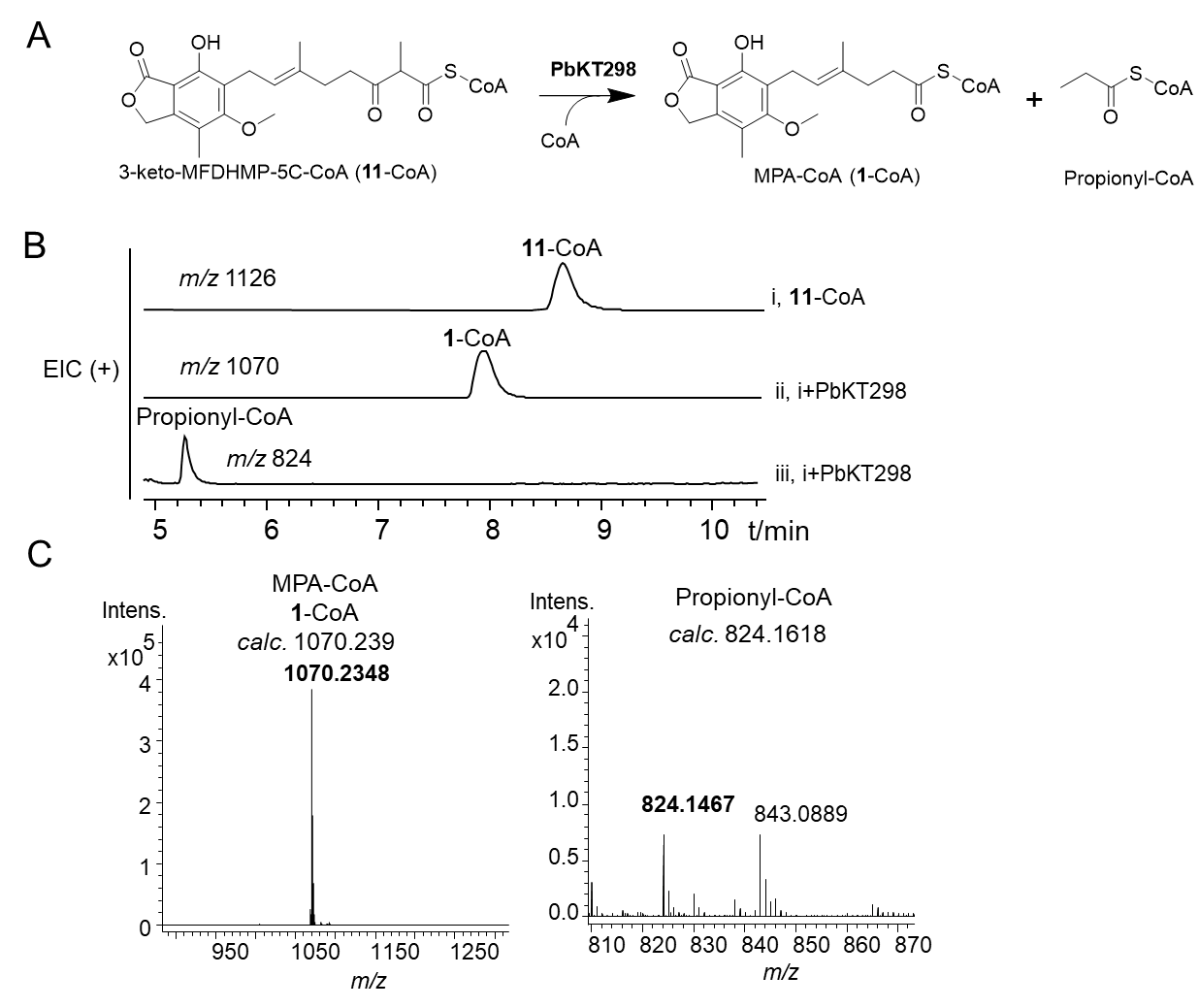


**Figure S42.** LC-HRMS analysis of the *in vitro* conversion from **11**-CoA to **1**-CoA catalyzed by PbKT298. A), The reaction schematic from **11**-CoA to **1**-CoA. B), (i) The EIC of **10**-CoA in the reaction of **6** with PbACL75, PbACOX323, PbECHD1071 and boiled PbKT298; (ii) The EIC of **1**-CoA in the reaction of **6** with PbACL75, PbACOX323, PbECHD1071 and PbKT298; (iii) The EIC of Propionyl-CoA in the reaction of **6** with PbACL75, PbACOX323, PbECHD1071 and PbKT298. C), The *m/z* values of **1**-CoA, [M+H]^+^ 1070.2348 (*obs*.), *calc.* 1070.239; Propionyl-CoA, [M+H]^+^ 824.1467 (*obs*.), *calc.* 824.1618.


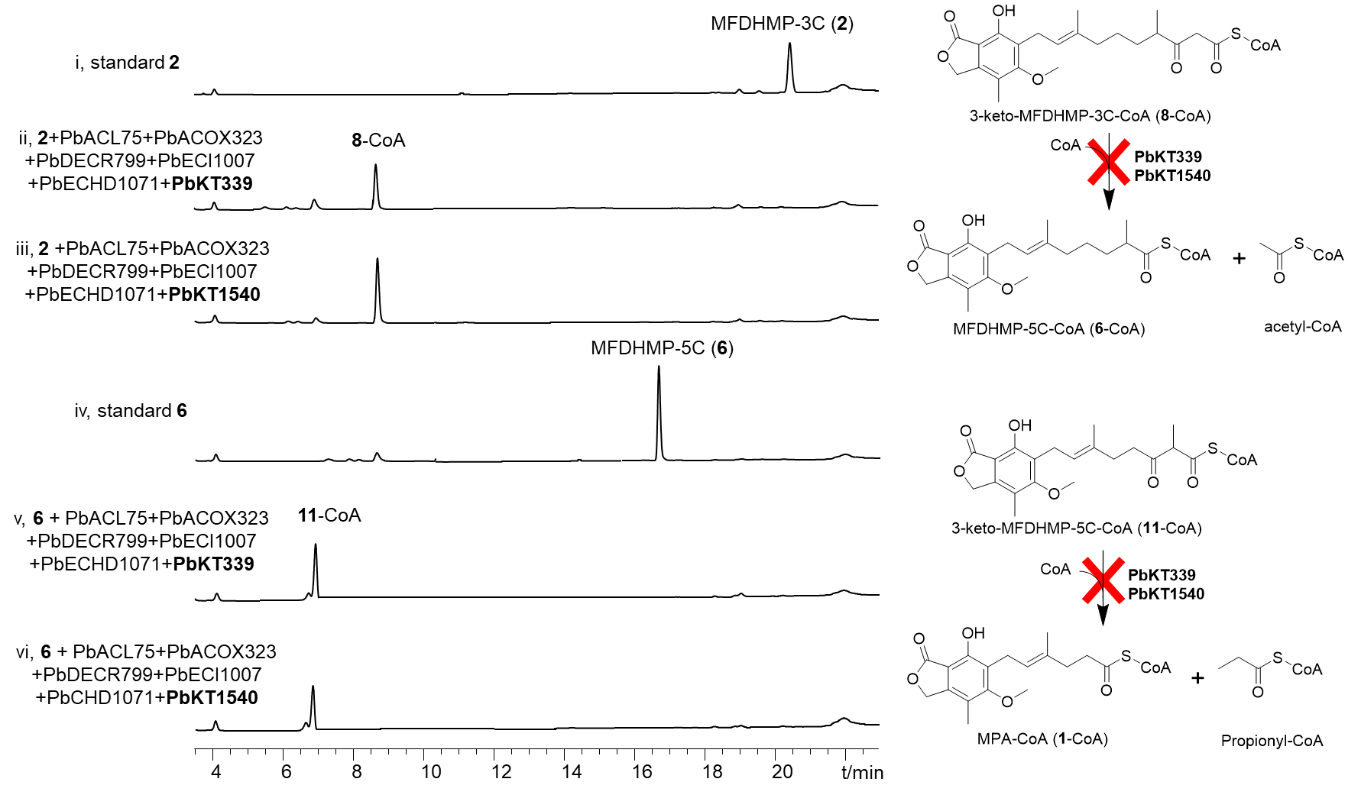


**Figure S43.** HPLC analysis the reaction of PbKT339 or PbKT1540 with **8**-CoA or **11**-CoA. i) Standard of **2**; ii-iii) The reaction of **2** with PbKT339 or PbKT1540 in the presence of PbACL75, PbACOX323, PbDECR799, PbECI1007, PbECHD1071, FAD, NADPH, ATP, CoA, Mg^2+^ and NAD^+^, respectively; iv) Standard of **6**; v-vi) The reaction of **6** with PbKT339 or PbKT1540 in the presence of PbACL75, PbACOX323, PbDECR799, PbECI1007, PbECHD1071, FAD, NADPH, ATP, CoA, Mg^2+^ and NAD^+^, respectively.


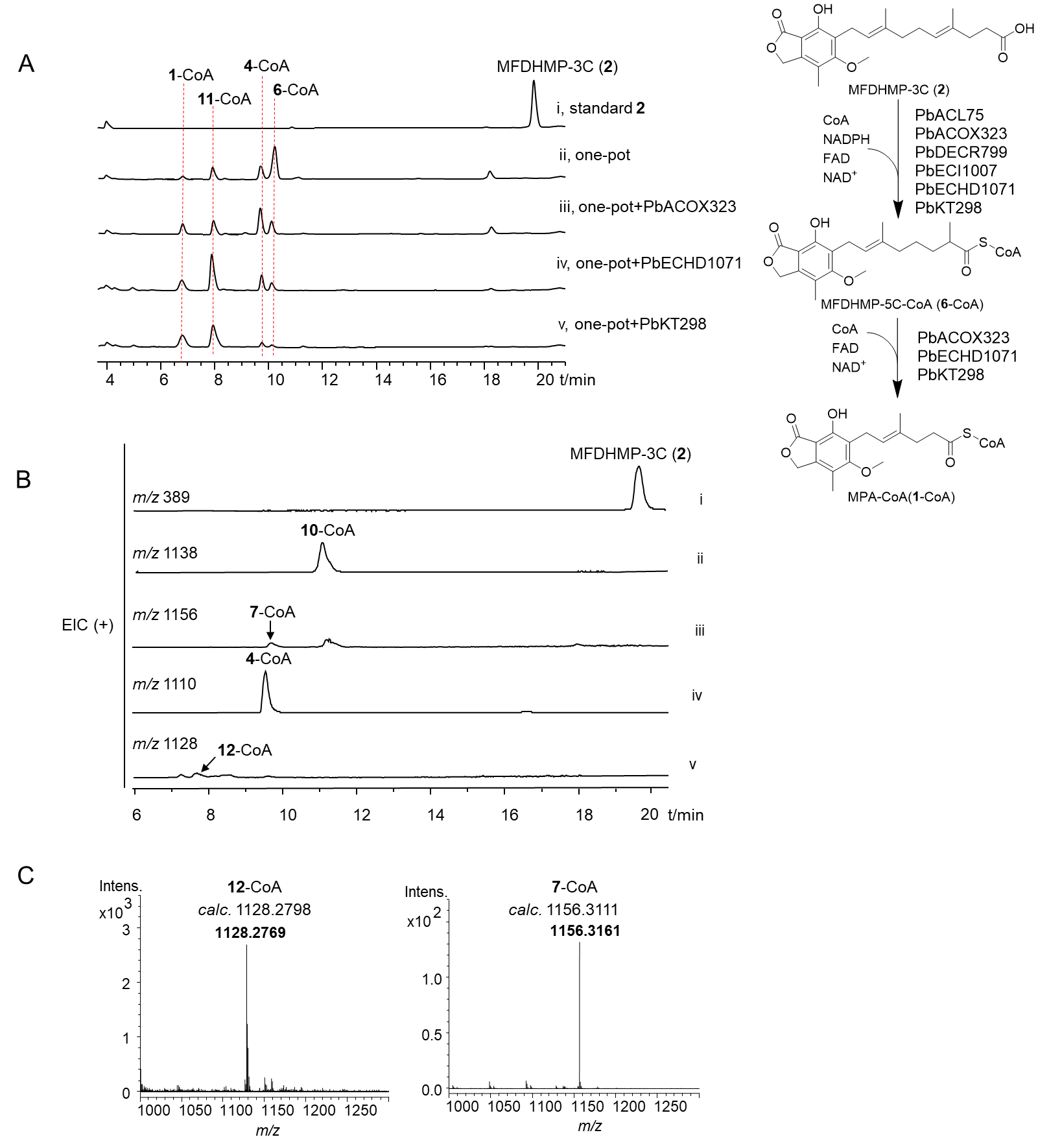


**Figure S44.** The six *β*-oxidation enzymes, MpaH’ (from *Pb*864) and cofactors together with MFDHMP-3C in a one-pot enzymatic reaction. A), HPLC analysis (254 nm) analysis of the one-pot enzymatic reaction. (i) The one-pot reaction control including the boiled identified 6 peroxisomal enzymes (5 μM each) toward substrate **2** for 3 h; (ii) The one-pot reaction including the identified 6 peroxisomal enzymes (5 μM each) toward substrate **2** for 3 h; (iii) after the initial 3 h “one-pot” enzymatic incubation, another 5 μM of PbACOX323 added to the ii; (iv) after the initial 3 h “one-pot” enzymatic incubation, another 5 μM PbECHD1071 enzyme added to the ii; (v) after the initial 3 h “one-pot” enzymatic incubation, another 5 μM PbKT298 enzyme added to the ii. B), LC-HRMS analysis of the one-pot enzymatic reaction. (i) The EIC of **2** in the control reaction with boiled *β*-oxidation enzymes; (ii-v) The EICs of **10**-CoA/**7**-CoA/**4**-CoA/**12**-CoA in the one-pot enzymatic reaction (Figure S44A, trace ii). C), The *m/z* value of **12**-CoA, [M+H]^+^= 1128.2769 (*obs*.), *calc* 1128.2798; **11**-CoA, [M+H]^+^= 1156.3161 (*obs*.), *calc* 1156.3111.


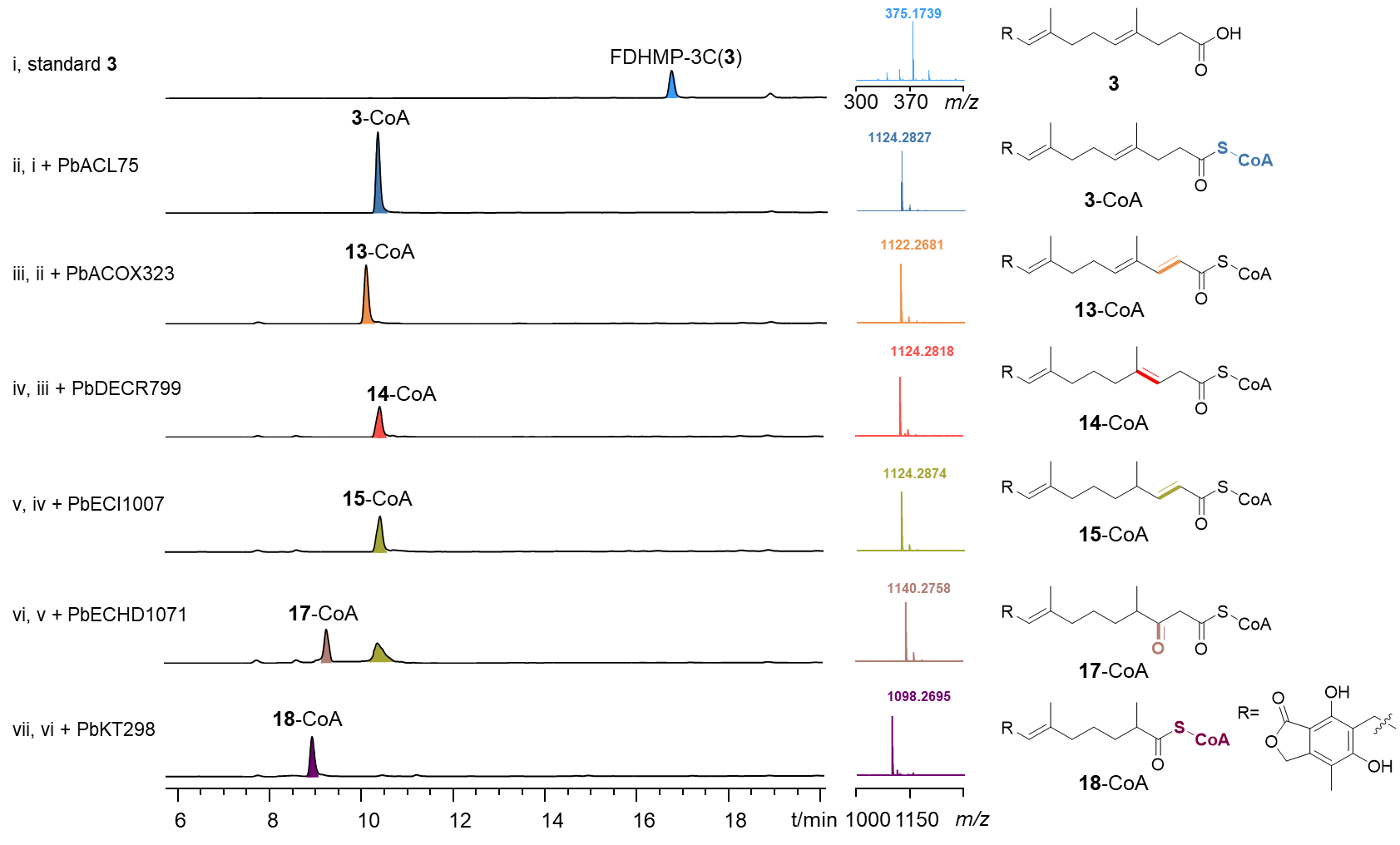


**Figure S45.** HPLC analysis (254 nm) of enzymatic reactions of peroxisomal *β*-oxidative production of **18**-CoA. i) The standard of FDHMP-3C; ii) CoA ligation reaction mediated by PbACL75 toward **3**; iii) Acyl-CoA oxidase PbACOX323 was added into the step (ii) reaction finished mixture, converting **3**-CoA to **13**-CoA; iv) Peroxisomal 2,4-*trans*-dienoyl-CoA reductase PbDECR799 was added into the step (iii) reaction finished mixture, which led to the production of 3-*trans*-enoyl-CoA esters (**14**-CoA); v) Enoyl-CoA isomerase PbECI1007 was further introduced into the step (iv) reaction finished mixture, isomerizing 3-*trans*-enoyl-CoA (**14**-CoA) to 2-*trans*-enoyl-CoA (**15**-CoA); vi) Enoyl-CoA hydratase and 3-hydroxyacyl-CoA dehydrogenase enzyme PbECHD1071 was introduced into the step (v) reaction finished mixture, resulting in the installation of the 3-keto group of the **17**-CoA; vii) Addition of 3-ketoacyl-CoA thiolase PbKT298 to the step (vi) reaction finished mixture led to the detection of the two carbon-shorter product **18**-CoA together with acetyl-CoA (see Figure S46).


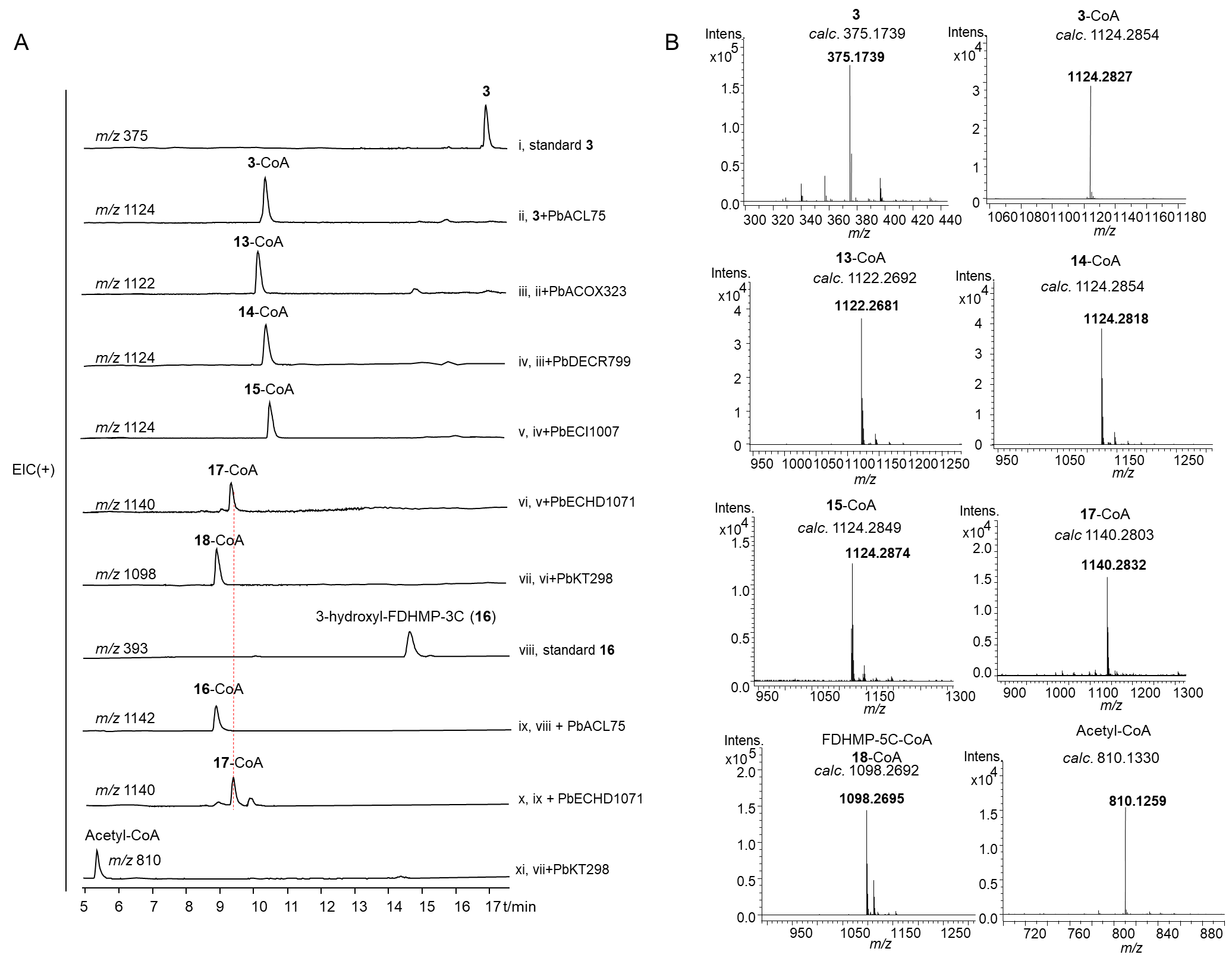


**Figure S46.** LC-HRMS analysis of the *in vitro* conversion from **3** to **18**-CoA catalyzed by purified PbACL75, PbACOX323, PbDECR799, PbECI1007, PbECHD1071 and PbKT298. A), (i) The EIC of standard **3**; (ii) The EIC of **3**-CoA in the reaction of **3** with PbACL75; (iii) The EIC of **13**-CoA in the reaction of **3** with PbACL75, PbACOX323; (iv) The EIC of **14**-CoA in the reaction of **3** with PbACL75, PbACOX323, PbDECR799; (v) The EIC of **15**-CoA in the reaction of **3** with PbACL75, PbACOX323, PbDECR799, PbECI1007; (vi) The EIC of **17**-CoA in the reaction of **3** with PbACL75, PbACOX323, PbDECR799, PbECI1007 and PbECHD1071; (vii) The EIC of **18**-CoA in the reaction of **3** with PbACL75, PbACOX323, PbDECR799, PbECI1007, PbECHD1071 and PbKT298; (viii) The EIC of standard **16**; (ix) The EIC of **16**-CoA in the reaction of **16** with PbACL75; (x) The EIC of **17**-CoA in the reaction of **16** with PbACL75 and PbECHD1071; (xi) The EIC of acetyl-CoA in the reaction of **3** with PbACL75, PbACOX323, PbDECR799, PbECI1007, PbECHD1071 and PbKT298. B), The *m/z* values of **3**: [M+H]^+^ = 375.1739 (*obs*.), *calc*. 375.1808; **3**-CoA: [M+H]^+^= 1124.2827 (*obs*.), *calc*. 1124.2854; **13**-CoA, [M+H]^+^= 1122.2681 (*obs*.), *calc*. 1122.2692; **14**-CoA, [M+H]^+^= 1124.2818 (*obs*.), *calc.* 1124.2854; **15**-CoA: [M+H]^+^= 1124.2874 (*obs*.), *calc*. 1124.2849; **17**-CoA, [M+H]^+^= 1140.2832 (*obs*.), *calc.* 1140.2803; **18**-CoA, [M+H]^+^= 1098.2695 (*obs*.), *calc*. 1098.2692; and acetyl-CoA, [M+H]^+^= 810.1259 (*obs*.), *calc*. 810.1330.

**Figure S47.** The complete *β*-oxidative biosynthesis pathway of DMMPA mediated by peroxisomal enzymes from *Pb*864. PbACL, acyl-CoA ligase; PbACOX, acyl-CoA oxidase; PbDECR, 2,4-dienoyl-CoA reductase; PbECI, enoyl-CoA isomerase; PbECHD, enoyl-CoA hydratase and 3-hydroxyacyl-CoA dehydrogenase; PbKT, 3-ketoacyl-CoA thiolase; MpaH’, acyl-CoA hydrolase.


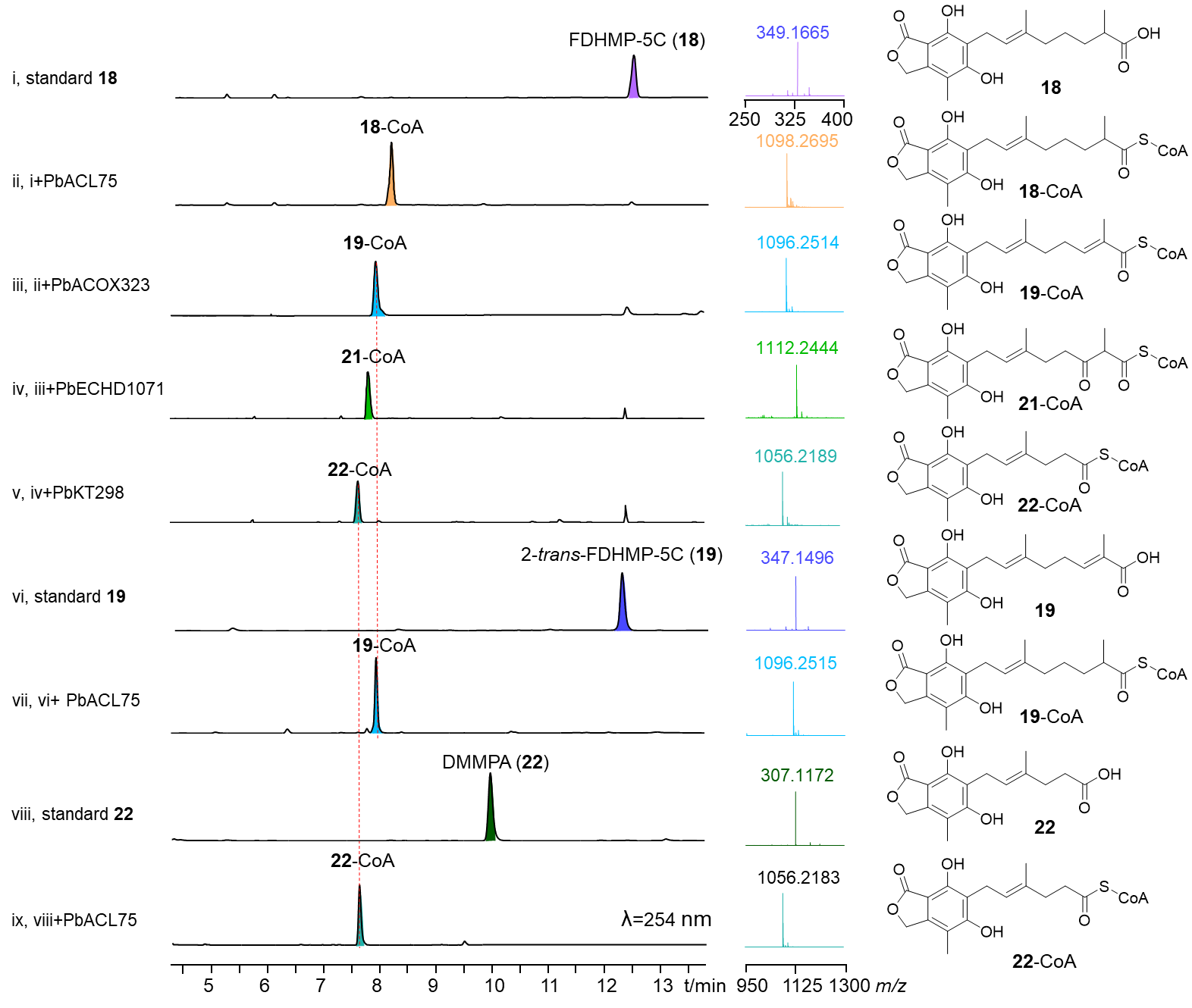


**Figure S48.** HPLC analysis of the *in vitro* conversion from FDHMP-5C (**18**) to **22**-CoA catalyzed by PbACL75, PbACOX323, PbECHD1071 and PbKT298. i) The standard of FDHMP-5C (**18**); ii) CoA ligation reaction mediated by PbACL75 toward **18**; iii) Acyl-CoA oxidase PbACOX323 was added into the step (ii) reaction finished mixture, converting **18**-CoA to 1**9**-CoA; iv) Enoyl-CoA hydratase and 3-hydroxyacyl-CoA dehydrogenase enzyme PbECHD1071 was introduced into the step (iii) reaction finished mixture, resulting in the installation of the 3-keto group of **21**-CoA; v) Addition of 3-ketoacyl-CoA thiolase PbKT298 to the step (iv) reaction finished mixture led to the detection of the two carbon-shorter product **22**-CoA together with Propionyl-CoA (see Figure S49); vi) The standard of 2-*trans*-MFDHMP-5C (**19**); vii) CoA ligation reaction mediated by PbACL75 toward 2-*trans*-MFDHMP-5C (**19**); viii) The standard of DMMPA (**22**); ix) CoA ligation reaction mediated by PbACL75 toward DMMPA (**22**).


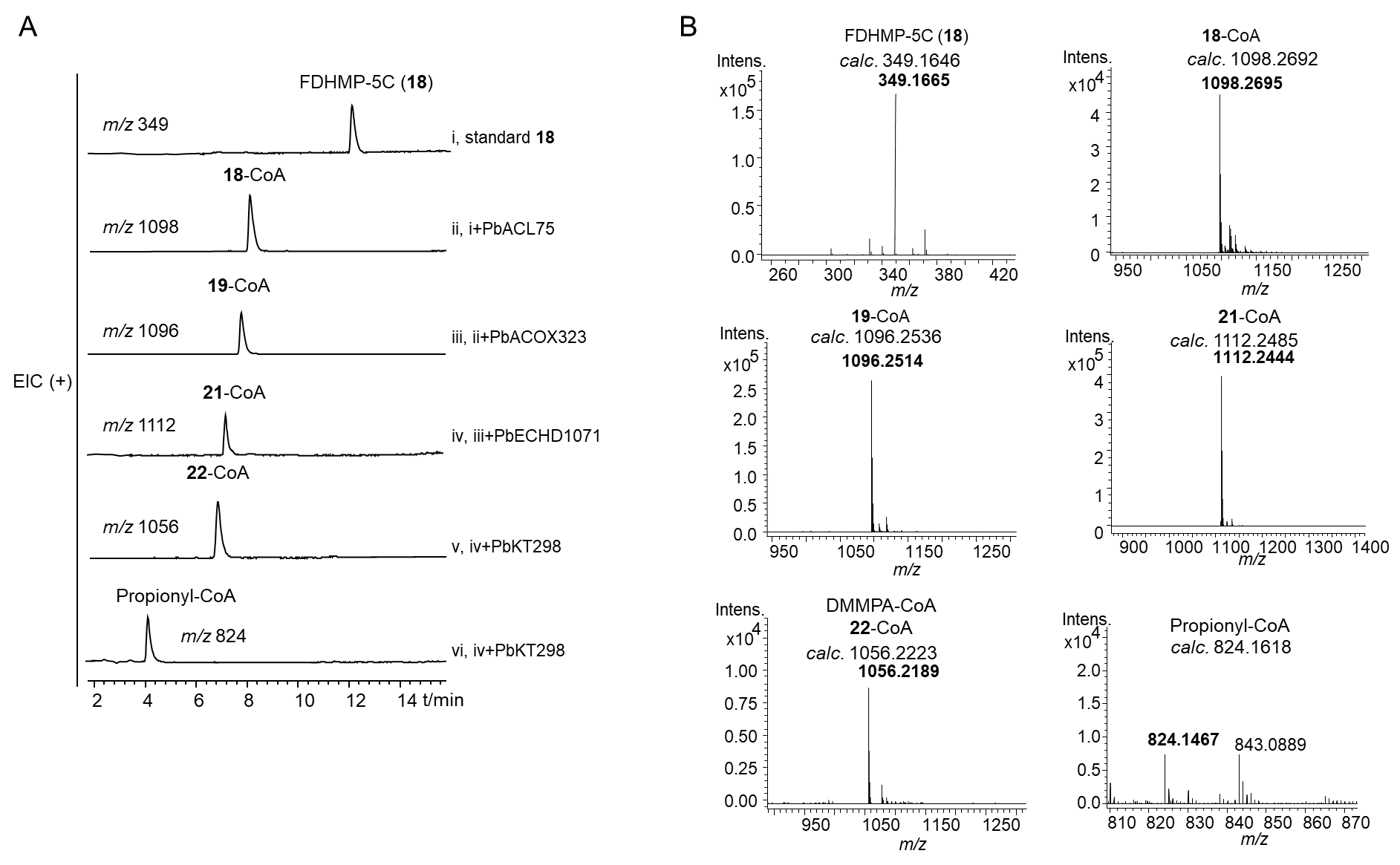


**Figure S49.** LC-HRMS analysis of the *in vitro* conversion from **18** to **22**-CoA catalyzed by PbACL75, PbACOX323, PbECHD1071 and PbKT298. A), (i) The EIC of standard **18**; (ii) The EIC of **18**-CoA in the reaction of **18** with PbACL75; (iii) The EIC of **19**-CoA in the reaction of **18** with PbACL75, PbACOX323; (iv) The EIC of **21**-CoA in the reaction of **18** with PbACL75, PbACOX323 and PbECHD1071; (v) The EIC of **22**-CoA in the reaction of **18** with PbACL75, PbACOX323, PbECHD1071 and PbKT298; (vi) The EIC of propionyl-CoA in the reaction of **3** with PbACL75, PbACOX323, PbECHD1071 and PbKT298. B), The *m/z* values of **18**, [M+H]^+^= 349.1665 (*obs*.), *calc*. 349.1646; **18**-CoA, [M+H]^+^= 1098.2695 (*obs*.), *calc*. 1098.2692; 1**9**-CoA, [M+H]^+^= 1096.2514 (*obs*.), *calc*. 1096.2536; **21**-CoA, [M+H]^+^= [M+H]^+^= 1112.2444 (*obs*.), *calc.* 1112.2485; **22**-CoA, [M+H]^+^= 1056.2289 (*obs*.), *calc.* 1056.2223; Propionyl-CoA, [M+H]^+^ 824.1467 (*obs*.), *calc.* 824.1618.


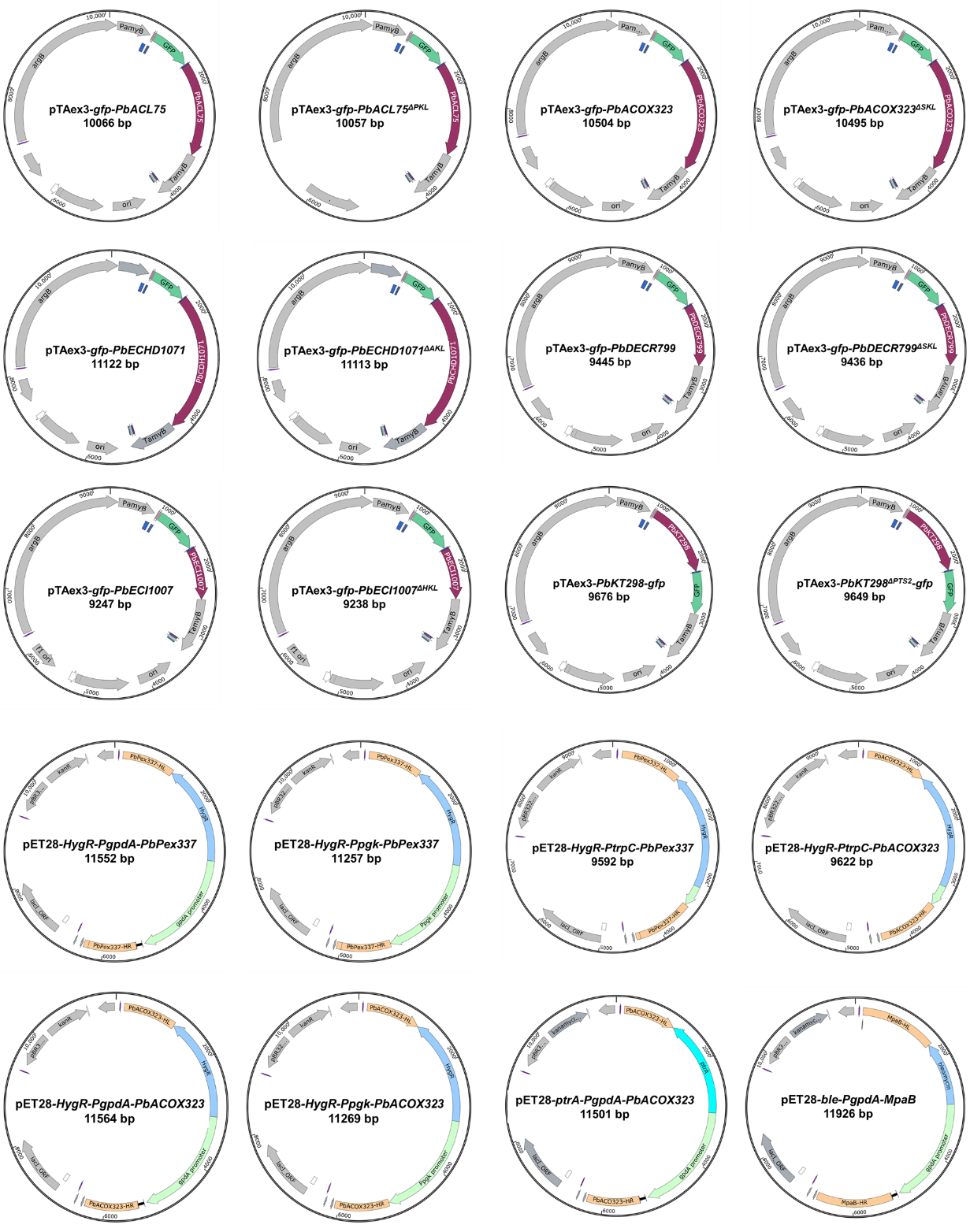


**Figure S50.** The map of constructed vectors in this study


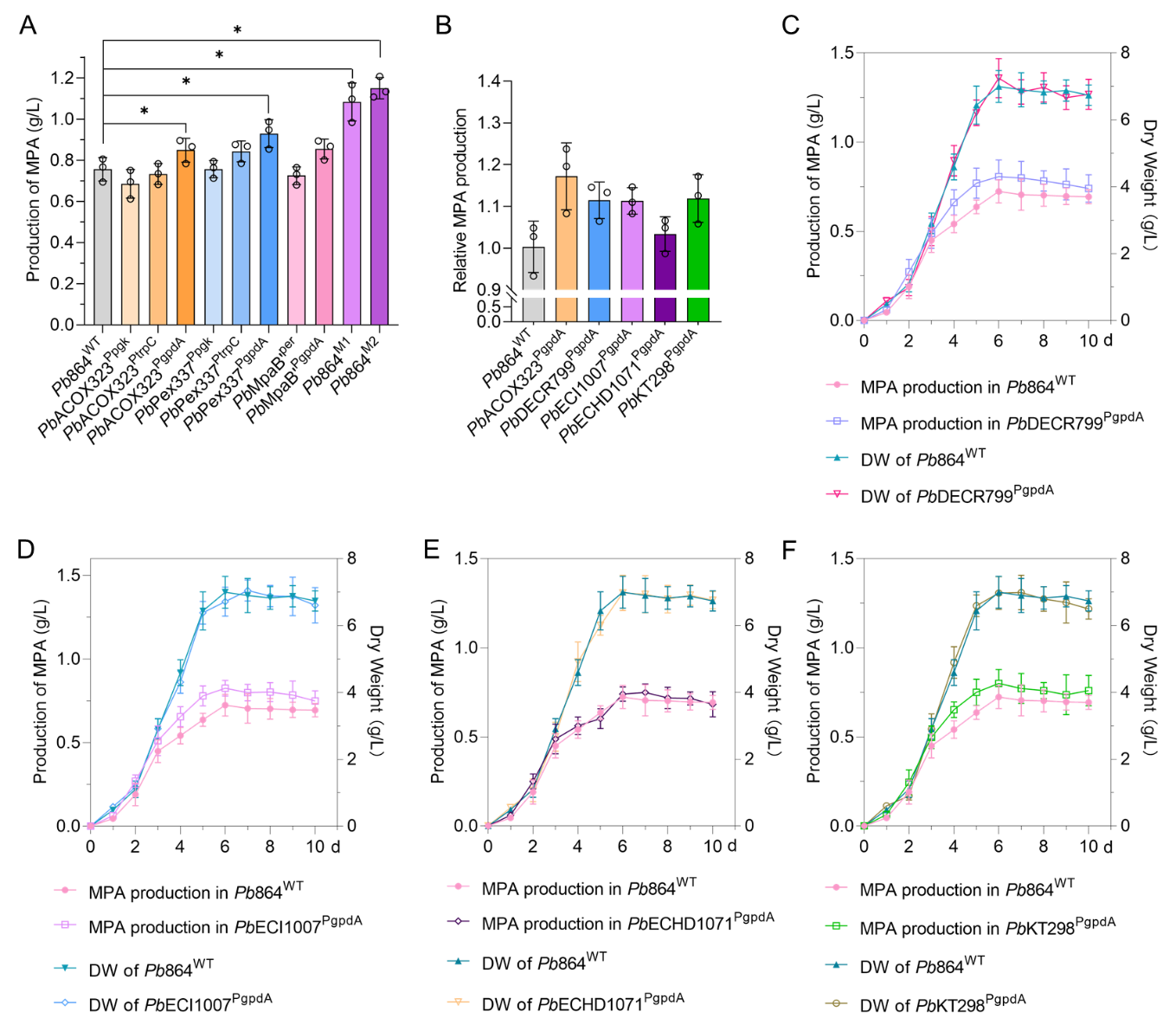


**Figure S51.** Compartmentalization engineering to increase the production of **1** in *Pb*864. A), The production of **1** by *Pb*864^WT^ and mutants *Pb*ACOX323^PtrpC^, *Pb*ACOX323^Ppgk^, *Pb*ACOX323^PgpdA^, *Pb*Pex337^Ppgk^, *Pb*Pex337^PtrpC^, *Pb*Pex337^PgpdA^, *Pb*MpaB’^PgpdA^, *Pb*MpaB’^per^, *Pb*864^M1^ and *Pb*864^M2^ at 6 days of fermentation. The ER-bound MpaB’ was spatially redirected to the outer membrane of the peroxisome by fusing the anchoring protein of Pex15 to the *C*-termini, resulting in the corresponding mutant *Pb*MpaB’^per^. B), The relative production of **1** by *Pb*864^WT^ and mutants *Pb*ACOX323^PgpdA^, *Pb*DECR7999^PgpdA^, *Pb*ECI1007^PgpdA^, *Pb*ECHD1071^PgpdA^, and *Pb*KT298^PgpdA^ by 6 days of fermentation. C-F), Dry cell weight (DW) and the production of **1** by *Pb*864^WT^, *Pb*DECR7999^PgpdA^, *Pb*ECI1007^PgpdA^, *Pb*ECHD1071^PgpdA^, and *Pb*KT298^PgpdA^ during 10 days of fermentation. The production of **1** was calculated by plotting the integrated peak area to the standard curve; chloramphenicol was used as an internal standard (* 0.01< *p* <0.05). Data are shown as the mean ± SD of triplicate measurements.

**Supplementary Tables**

**Table S1** The *β*-Oxidation Enzyme Gen

| *β*-oxidation enzymes | Probe Source | Gene probe | *Pb*864 Gene | Targeting signal | Identities/  Positives % | Fpkm | Function |
| --- | --- | --- | --- | --- | --- | --- | --- |
| CoA Ligase | *S. cerevisiae* | *FAA2* | *PbACL75* | PKL | 23/39 | 11 | Yes |
|  |  |  | *PbACL891* | AKL | 72/85 | 48 | Yes |
|  |  |  | *PbACL243* | AKL | 25/40 | 17 | No |
|  |  |  | *PbACL791* | AKL | 25/41 | 10 | No |
|  |  |  | *PbACL1224* | ARL | 26/39 | 8 | Yes |
|  |  |  | *PbACL1333* | SKL | 22/35 | 20 | No |
|  |  |  | *PbACL1637* | ARL | 32/52 | 6 | No |
| Acyl-CoA Oxidase | *S. cerevisiae* | *Fox1p* | *PbACOX323* | SKL | 31/50 | 20 | Yes |
|  | *P. chrysogenum* | *Pc13g14410* | *PbACOX323* | SKL | 44/63 | 20 | Yes |
|  |  |  | *PbACOX692* | SHL | 26/43 | 106 | No |
|  |  | *Pc20g01800* | *PbACOX323* | SKL | 92/97 | 20 | Yes |
|  |  |  | *PbACOX103* | AKL | 85/90 | 35 | No |
|  |  |  | *PbACOX104* | AKL | 50/66 | 46 | No |
|  |  | *Pc21g17590* | *PbACOX103* | AKL | 46/67 | 35 | No |
|  |  | *Pc22925150* | *PbACOX104* | AKL | 79/81 | 46 | No |
| Enoyl-CoA Hydratase/3-Hydroxyacyl-CoA Dehydrogenase | *S. cerevisiae* | *Fox2p* | *PbECHD1071* | AKL | 48/62 | 93 | Yes |
|  |  |  | *PbECHD375* | SKL | 27/39 | 23 | No |
|  |  |  | *PbECHD99* | SKM | 21/37 | 6 | No |
|  | *P. chrysogenum* | *Pc13g05940* | *PbECHD1071* | AKL | 94/97 | 93 | Yes |
|  |  |  | *PbECHD762* | RL-X_5_-HL | 39/55 | 33 | No |
|  |  | *Pc13g02710* | *PbECHD843* | SKL | 84/90 | 5 | No |
| Thiolase | *S. cerevisiae* | *Fox3p* | *PbKT298* | RL-X_5_-QL | 43/59 | 154 | Yes |
|  |  |  | *PbKT339* | RL-X_5_-HF | 46/63 | 76 | No |
|  | *P. chrysogenum* | *Pc15g00410* | *PbKT298* | RL-X_5_-QL | 94/97 | 154 | Yes |
|  |  | *Pc22g06820* | *PbKT339* | RL-X_5_-HF | 95/98 | 76 | No |
|  |  | *Pc13g12930* | *PbKT1540* | RL-X_5_-HF | 94/98 | 45 | No |
| 2,4-dienoyl-CoA reductase | *S. cerevisiae* | *SPS19* | *PbDECR799* | SKL | 75/80 | 106 | Yes |
| Enoyl-CoA isomerase | *S. cerevisiae* | *ECI1* | *PbECI1007* | HKL | 83/85 | 21 | Yes |

**Table S2** The Steady-State Kinetic Analysis

| Enzyme | Substrate | *K*_m_  (μM) | *k*_cat_  (min^-1^) | *k*_cat_/*K*_m_  (mM^-1^ min^-1^) |
| --- | --- | --- | --- | --- |
| PbACL75 | MFDHMP-3C | 14.75±4.69 | 2.52±0.24 | 171.01 |
|  | FDHMP-3C | 10.38±1.58 | 1.82±0.14 | 175.61 |
|  | 2-trans-MFDHMP-5C | 3.84±0.26 | 2.31±0.02 | 601.10 |
| PbACL891 | MFDHMP-3C | 2.43±0.23 | 0.11±0.01 | 46.65 |
|  | FDHMP-3C | 0.97±0.21 | 0.05±0.01 | 52.57 |
| PbACL1224 | MFDHMP-3C | 10.13±1.41 | 1.51±0.01 | 149.06 |
|  | FDHMP-3C | 9.09±1.94 | 1.34±0.01 | 147.41 |
| PbACOX323 | MFDHMP-3C-CoA | 39.11±2.92 | 58.07±3.93 | 1484.78 |
| PbDECR799 | 2,4-*trans*-dienoyl-MFDHMP-3C-CoA | 18.77±3.04 | 6.8±1.08 | 362.49 |
| PbECHD1071 | 2-*trans*-enoyl-MFDHMP-3C-CoA | 42.9±6.7 | 5.51±0.24 | 128.42 |

**Table S3** ^1^H (600 MHz) and ^13^C (150 MHz) NMR Data of 2,4-*trans*-Dienoyl-MFDHMP-3C (**5**) in CD_3_OD.

| 2,4-*trans*-dienoyl-MFDHMP-3C (**5**) | | |
| --- | --- | --- |
| Position | *δ*_C_ | *δ*_H_, *J* (Hz) |
| 1 | 179.54 |  |
| 2 | 30.47 | 5.69, *d*, (15.5) |
| 3 | 30.77 | 7.06, *d*, (15.4) |
| 4 | 134.24 |  |
| 5 | 124.7 | 5.73, *t*, (7.3) |
| 6 | 27.92 | 2.31, m |
| 7 | 39.65 | 2.10, *t*, (6.6) |
| 8 | 135.14 |  |
| 9 | 123.77 | 5.21, *t*, (7.1) |
| 10 | 23.57 | 3.39, *d*, (6.7) |
| 11 | 117.85 |  |
| 12 | 164.79 |  |
| 13 | 116.35 |  |
| 14 | 146.6 |  |
| 15 | 70.86 | 5.25, *s* |
| 16 | 173.93 |  |
| 17 | 107.67 |  |
| 18 | 156.98 |  |
| 19 | 11.41 | 2.14, *s* |
| 20 | 16.25 | 1.69, *s* |
| 21 | 12.22 | 1.80, *s* |
| 22 | 61.47 | 3.76, *s* |

**Table S4** ^1^H (600 MHz) and ^13^C (150 MHz) NMR Data of 3-*trans*-Enoyl-MFDHMP-3C (**9**) in CD_3_OD.

| 3-*trans*-enoyl-MFDHMP-3C (**9**) | | |
| --- | --- | --- |
| Position | *δ*_C_ | *δ*_H_, *J* (Hz) |
| 1 | 175.47 |  |
| 2 | 33.286 | 2.96, *d*, (7.1) |
| 3 | 122.54 | 5.20, *t*, (6.8) |
| 4 | 138.07 |  |
| 5 | 38.50 | 1.95, *t*, (overlapped) |
| 6 | 25.46 | 1.50, m |
| 7 | 38.63 | 1.95, *t*, (7.7) |
| 8 | 134.69 |  |
| 9 | 122.54 | 5.25, *t*, (overlapped) |
| 10 | 22.17 | 3.40, *d*, (6.8) |
| 11 | 116.53 |  |
| 12 | 163.40 |  |
| 13 | 116.41 |  |
| 14 | 145.12 |  |
| 15 | 69.43 | 5.25, *s* |
| 16 | 172.48 |  |
| 17 | 106.28 |  |
| 18 | 153.25 |  |
| 19 | 9.98 | 2.16, *s* |
| 20 | 14.79 | 1.77, *s* |
| 21 | 14.72 | 1.59, *s* |
| 22 | 60.1 | 3.77, *s* |

**Table S5** ^1^H (600 MHz) and ^13^C (150 MHz) NMR Data of 2-*trans*-Enoyl-MFDHMP-3C (**10**) in CD_3_OD.

| 2-*trans*-enoyl-MFDHMP-3C (**10**) | | |
| --- | --- | --- |
| Position | *δ*_C_ | *δ*_H_, *J* (Hz) |
| 1 | 172.50 |  |
| 2 | 122.65 | 5.70, *d*, (15.3) |
| 3 | 145.13 | 6.71, *dd*, (15.6) |
| 4 | 34.30 | 2.28, m |
| 5 | 32.45 | 1.36, *t* |
| 6 | 25.71 | 1.26, m |
| 7 | 39.10 | 1.97, *t*, (6.6) |
| 8 | 133.28 |  |
| 9 | 124.36 | 5.20, *t*, (overlapped) |
| 10 | 22.18 | 3.39, *d*, (6.7) |
| 11 | 122.51 |  |
| 12 | 163.39 |  |
| 13 | 116.34 |  |
| 14 | 134.30 |  |
| 15 | 69.44 | 5.20, *s* |
| 16 | 175.81 |  |
| 17 | 106.28 |  |
| 18 | 153.29 |  |
| 19 | 9.99 | 2.07, *s* |
| 20 | 14.64 | 1.77, *s* |
| 21 | 14.90 | 0.99, *d*, (6.72) |
| 22 | 60.06 | 3.31, *s* |

**Table S6** The Plasmids Used in This Study

| Plasmid | Relevant characteristics | Reference |
| --- | --- | --- |
| pTAex3 | Fungal expression vector that harbors the *α*-amylase promoter/terminator (*PamyB/TamyB*) and the *argB* selective marker. | Ref.^[23,24]^ |
| pTAex3-*rfp^SKL^* | pTAex3 harboring *rfp^SKL^* for localization of RFP to peroxisome. | Ref.^[1]^ |
| pTAex3-*gfp* | pTAex3 harboring *gfp* for heterologous expression of GFP in *Ao*M-2-3. | Ref.^[1]^ |
| pTAex3-*gfp-PbACL75* | pTAex3 harboring the *N-gfp-PbACL75-C* fusion gene for studying the subcellular location of PbACL75. | This study |
| pTAex3-*gfp-PbACL75^ΔPKL^* | pTAex3 harboring *N-gfp-PbACL75^ΔPKL^-C* for verifying the role of the *C*-terminal PKL sequence in PbACL75 for its peroxisomal localization. | This study |
| pTAex3-*gfp-PbACOX323* | pTAex3 harboring the *N-gfp-PbACOX323-C* fusion gene for studying the subcellular location of PbACOX323. | This study |
| pTAex3-*gfp-PbACOX323^ΔSKL^* | pTAex3 harboring *N-gfp-PbACOX323^ΔSKL^-C* for verifying the role of the *C*-terminal SKL sequence in PbACOX323 for its peroxisomal localization. | This study |
| pTAex3-*gfp-PbECH1071* | pTAex3 harboring the *N-gfp-PbECHD1071-C* fusion gene for studying the subcellular location of PbECHD1071. | This study |
| pTAex3-*gfp-PbECH1071^ΔAKL^* | pTAex3 harboring *N-gfp-PbECH1071^ΔAK^-C* for verifying the role of the *C*-terminal AKL sequence in PbECHD1071 for its peroxisomal localization. | This study |
| pTAex3-*gfp-PbDECR799* | pTAex3 harboring the *N-gfp-PbDECR799-C* fusion gene for studying the subcellular location of PbDECR799. | This study |
| pTAex3-*gfp-PbDECR799^ΔSKL^* | pTAex3 harboring *N-gfp-PbDECR799^ΔSK^-C* for verifying the role of the *C*-terminal SKL sequence in PbDECR799 for its peroxisomal localization. | This study |
| pTAex3-*gfp-PbECI1007* | pTAex3 harboring the *N-gfp-PbECI1007-C* fusion gene for studying the subcellular location of PbECI1007. | This study |
| pTAex3-*gfp-PbECI1007^ΔHKL^* | pTAex3 harboring *N-gfp-PbECI1007^ΔHKL^-C* for verifying the role of the *C*-terminal HKL sequence in PbECI1007 for its peroxisomal localization. | This study |
| pTAex3*-PbKT298*-*gfp* | pTAex3 harboring the *N-PbKT298*-*gfp-C* fusion gene for studying the subcellular location of PbKT298. | This study |
| pTAex3*-PbKT298^ΔPTS2^*-*gfp* | pTAex3 harboring *N-PbKT298^ΔPTS2^*-*gfp-C* for verifying the role of the *C*-terminal PTS2 sequence in PbKT298 for its peroxisomal localization. | This study |
| pET28b*-HygR-PgpdA-PbPex337* | pET28b harboring the *HygR* resistance gene cassette which is flanked by the upstream, *gpdA* promoter and downstream sequences of *PbPex337* for in-frame insertion of *PgpdA* in *Pb*864 by split-marker recombination strategy. | This study |
| pET28b*-HygR-Ppgk-PbPex337* | pET28b harboring the *HygR* resistance gene cassette which is flanked by the upstream, *pgk* promoter and downstream sequences of *PbPex337* for in-frame insertion of *Ppgk* in *Pb*864 by split-marker recombination strategy. | This study |
| pET28b*-HygR-PtrpC-PbPex337* | pET28b harboring the *HygR* resistance gene cassette which is flanked by the upstream, *trpC* promoter and downstream sequences of *PbPex337* for in-frame insertion of *PtrpC* in *Pb*864 by split-marker recombination strategy. | This study |
| pET28b*-HygR-PgpdA-PbACOX323* | pET28b harboring the *HygR* resistance gene cassette which is flanked by the upstream, *gpdA* promoter and downstream sequences of *PbACOX323* for in-frame insertion of *PgpdA* in *Pb*864 by split-marker recombination strategy. | This study |
| pET28b*-HygR-Ppgk-PbACOX323* | pET28b harboring the *HygR* resistance gene cassette which is flanked by the upstream, *pgk* promoter and downstream sequences of *PbACOX323* for in-frame insertion of *Ppgk* in *Pb*864 by split-marker recombination strategy. | This study |
| pET28b*-HygR-PtrpC-PbACOX323* | pET28b harboring the *HygR* resistance gene cassette which is flanked by the upstream, *trpC* promoter and downstream sequences of *PbACOX323*for in-frame gene insertion of *PtrpC* in *Pb*864 by split-marker recombination strategy. | This study |
| pET28b-*PbACL891* | pET-28b harboring the codon optimized *PbACL891* gene for heterologous expression of PbACL891 in *E. coli* BL21(DE3). | This study |
| pET28b-*PbACL75* | pET-28b harboring the codon optimized *PbACL75* gene for heterologous expression of PbACL75 in *E. coli* BL21(DE3). | This study |
| pET28b-*PbACL1224* | pET-28b harboring the codon optimized *PbACL1224* gene for heterologous expression of PbACL1224 in *E. coli* BL21(DE3). | This study |
| pET28b-*PbACL243* | pET28b harboring the codon optimized *PbACL243* gene for heterologous expression of PbACL243 in *E. coli* BL21(DE3). | This study |
| pET28b-*PbACL791* | pET28b harboring the codon optimized *PbACL791* gene for heterologous expression of PbACL791 in *E. coli* BL21(DE3). | This study |
| pET28b*-HygR- PgpdA-MpaB’^per^* | pET28b harboring the *HygR* resistance gene cassette which is flanked by the upstream, *gpdA* promoter and downstream sequences of *MpaB’^per^* for in-frame gene insertion of *PgpdA* in *Pb*864 by split-marker recombination strategy. | This study |

**Table S7** Primers Used in This Study

| Primers | Sequence (5’-3’) |
| --- | --- |
| TAex3-GFP-PbACL75-*Nde*I-FP | ggtggtggctggatcCATATGATGATTTTTGAA |
| pTAex3-GFP-PbACL75-*Nde*I-RP | agatccccgggtaccCTAAAGTTTGGGCTT |
| pTAex3-GFP-PbACOX323-*Nde*I-FP | ggtggtggctggatcCATATGATGCCATCTCCT |
| pTAex3-GFP-PbACOX323-*Nde*I-RP | agatccccgggtaccTTACAGCTTGCTCTT |
| pTAex3-GFP-PbDECR799-*Nde*I-FP | ggtggtggctggatcCATATGATGCCGAACGGC |
| pTAex3-GFP-PbDECR799-*Nde*I-RP | cagatccccgggtaccTCAAAGCTTCGACTTCTTCT |
| pTAex3-GFP-PbECI1007-*Nde*I-FP | ggtggtggctggatcCATATGATGTCAAACGAACAG |
| pTAex3-GFP-PbECI1007-*Nde*I-RP | agatccccgggtaccCTAAAGCTTATGCTTCTTCT |
| pTAex3-GFP-PbECHD1071-*Nde*I-FP | ggtggtggctggatcCATATGATGTCCGAACTACGA |
| pTAex3-GFP-PbECHD1071-*Nde*I-RP | actacagatccccgggtaccTTACAGCTTAGCCTT |
| pTAex3-PbKT298-GFP-KpnI-FP | ccgcgcggcagcgagGGTACCATGTCGTCAGCACA |
| pTAex3-PbKT298-GFP-KpnI-RP | gcccttgctcaccatATGCTCAGAAACGAA |
| pTAex3-GFP-PbACL75ΔPKL-RP | agatccccgggtaccCTACTTGATACTCCC |
| pTAex3-GFP-PbACOX323ΔSKL-RP | actacagatccccgggtaccTTACTTCGCAGGCTCGT |
| pTAex3-GFP-PbDECR799ΔSKL-RP | agatccccgggtaccTCACTTCTTCTTGCCGGTCAC |
| pTAex3-GFP-PbECI1007ΔSHL-RP | cagatccccgggtaccCTACTTCTTCTCGCCAGATG |
| pTAex3-GFP-PbECHD1071ΔAKL-RP | actacagatccccgggtaccTTACTTGGCGCCCTCAA |
| pTAex3-PbKT298ΔPTS2-GFP-FP | ccgcgcggcagcgagGGTACCATGTCGTCAGCACA |
| 323HL-PgpdA-FP | ggtggtggtggtggtATGTCCCCAGACATACAT |
| 323HL-PgpdA-RP | cctgggttgctcgagGTAAAGGTGACGTTTCCCGT |
| 323HR-PgpdA-FP | tgagcagacactATGCCCGGGGTTCATAACTG |
| 323HRPgpdA-RP | tccgtcgacaagcttTCAATTGACGAGAAG |
| 323HL-PtrpC-FP | ggtggtggtggtggtATGTCCCCAGACATACAT |
| 323HL-PtrpC-RP | cctgggttgctcgagGTAAAGGTGACGTTTCCCGT |
| 323HR-PtrpC-FP | tattctacccaagcaACTAGTCCCGGGGTTCATAAC |
| 323HR-PtrpC-RP | tccgtcgacaagcttTCAATTGACGAGAAG |
| 323HL-Ppgk-FP | ggtggtggtggtggtATGTCCCCAGACATACAT |
| 323HL-Ppgk-RP | cctgggttgctcgagGTAAAGGTGACGTTTCCCGT |
| 323HR-Ppgk-FP | aacgtcggcggccgcACTAATGCACTTTTTGTGTC |
| 323HR-Ppgk-RP | tccgtcgacaagcttTCAATTGACGAGAAG |
| 337HL-PgpdA-FP | ggtggtggtggtggtTTCGCCTTGTCCAGCTC |
| 337HL-PgpdA-RP | ccagcccctgggttgctcgagGCTCTTCAAGAGCGTC |
| 337HR-PgpdA-FP | tgagcagacactATGGTTGCCGACGCCCTC |
| 337HR-PgpdA-RP | tcgagctccgtcgacACCGGGTCTCCAGCGTAC |
| 337HL-PtrpC-FP | ggtggtggtggtggtTTCGCCTTGTCCAGCTC |
| 337HL-PtrpC-RP | ccagcccctgggttgctcgagGCTCTTCAAGAGCGTC |
| 337HR-PtrpC-FP | tattctacccaagcaACCGGGTCTCCAGCGTAC |
| 337HR-PtrpC-RP | tcgagctccgtcgacACCGGGTCTCCAGCGTAC |
| 337HL-Ppgk-FP | ggtggtggtggtggtTTCGCCTTGTCCAGCTC |
| 337HL-Ppgk-RP | ccagcccctgggttgctcgagGCTCTTCAAGAGCGTC |
| 337HR-Ppgk-FP | aacgtcggcggccgcACCGGGTCTCCAGCGTAC |
| 337HR-Ppgk-RP | tcgagctccgtcgacACCGGGTCTCCAGCGTAC |

**Table S8** The Yield of MPA From *Pb*864 Wild Type and Mutants.

| Time (day) | *Pb*864^WT^ | *Pb*864^M1^ | *Pb*864^M2^ |
| --- | --- | --- | --- |
| 1 | 0.06 | 0.06 | 0.05 |
| 2 | 0.20 | 0.27 | 0.28 |
| 3 | 0.45 | 0.65 | 0.67 |
| 4 | 0.55 | 0.92 | 0.99 |
| 5 | 0.65 | 1.08 | 1.11 |
| 6 | 0.77 | 1.03 | 1.15 |
| 7 | 0.71 | 0.97 | 1.05 |
| 8 | 0.67 | 0.97 | 1.03 |
| 9 | 0.64 | 0.94 | 0.97 |
| 10 | 0.67 | 0.89 | 0.93 |

**Reference**

[1] W. Zhang, L. Du, Z. Qu, et al., “Compartmentalized Biosynthesis of Mycophenolic Acid,” *Proc. Natl. Acad. Sci. U.S.A* **2019**, *116*, 13305-13310.

[2] W. Zhang, S. N. Cao, L. Qiu, et al., “Functional Characterization of MpaG', the O-Methyltransferase Involved in the Biosynthesis of Mycophenolic Acid,” *Chembiochem* **2015**, *16*, 565-569.

[3] Q. X. Zhou, J. T. Xu, Y. B. Kou, et al., “Differential Involvement of *β*-Glucosidases from Hypocrea Jecorina in Rapid Induction of Cellulase Genes by Cellulose and Cellobiose,” *Eukaryot. Cell* **2012**, *11*, 1371-1381.

[4] A. V. Qualley, J. R. Widhalm, F. Adebesin, C. M. Kish, N. Dudareva, “Completion of the Core *β*-Oxidative Pathway of Benzoic Acid Biosynthesis in Plants,” *Proc. Natl. Acad. Sci. U.S.A* **2012**, *109*, 16383-16388.

[5] S. Ferdinandusse, S. Denis, P. L. Faust, R. J. A. Wanders, “Bile Acids: the Role of Peroxisomes,” *J. Lipid Res.* **2009**, *50*, 2139-2147.

[6] J. Leon, “Role of Plant Peroxisomes in the Production of Jasmonic Acid-Based Signals,” *Subcell. Biochem.* **2013**, *69*, 299-313.

[7] T. B. Regueira, K. R. Kildegaard, B. G. Hansen, U. H. Mortensen, C. Hertweck, J. Nielsen, “Molecular Basis for Mycophenolic Acid Biosynthesis in *Penicillium brevicompactum*,” *Appl. Environ. Microbiol.* **2011**, *77*, 3035-3043.

[8] A. Del-Cid, C. Gil-Durán, I. Vaca, et al., “Identification and Functional Analysis of the Mycophenolic Acid Gene Cluster of *Penicillium roqueforti*,” *PLoS One* **2016**, *11*.

[9] X. W. Chen, L. Wang, J. M. Zhang, et al., “Immunosuppressant Mycophenolic Acid Biosynthesis Employs a New Globin-like Enzyme for Prenyl Side Chain Cleavage,” *Acta Pharm. Sin. B.* **2019**, *9*, 1253-1258.

[10] L. Canonica, Kroszczy.W, B. M. Ranzi, B. Rindone, Scolasti.C, “Biosynthesis of Mycophenolic Acid,” *J. Chem. Soc. Chem. Comm.* **1972**, 257-&.

[11] L. Colombo, C. Gennari, C. Scolastico, “Scolastico Biosynthesis of Mycophenolic Acid. Oxidation of 6-Farnesyl-5,7-Dihydroxy-4-Methylphthalide in a Cell-Free Preparation from *Penicillium-brevicompactum*,” *J. Chem. Soc. Chem. Comm.* **1978**, 434-434.

[12] L. Colombo, C. Gennari, D. Potenza, C. Scolastico, F. Aragozzini, “Biosynthesis of Citrinin and Synthesis of Its Biogenetic Precursors,” *J. Chem. Soc. Chem. Comm.* **1979**, 1021-1022.

[13] L. J. Knoll, D. R. Johnson, J. I. Gordon, “Biochemical Studies of Three *Saccharomyces cerevisiae* Acyl-CoA Synthetases, Faa1p, Faa2p, and Faa3p,” *J. Biol. Chem.* **1994**, *269*, 16348-16356.

[14] A. Dmochowska, D. Dignard, R. Maleszka, D. Y. Thomas, “Structure and Transcriptional Control of the *Saccharomyces cerevisiae* *POX1* Gene Encoding Acyl-Coenzyme A Oxidase,” *Gene* **1990**, *88*, 247-252.

[15] M. S. Alphey, W. H. Yu, E. Byres, D. Li, W. N. Hunter, “Structure and Reactivity of Human Mitochondrial 2,4-Dienoyl-CoA Reductase: Enzyme-Ligand Interactions in a Distinctive Short-Chain Reductase Active Site,” *J. Biol. Chem.* **2005**, *280*, 3068-3077.

[16] K. L. Fillgrove, V. E. Anderson, “The Mechanism of Dienoyl-CoA Reduction by 2,4-Dienoyl-CoA Reductase is Stepwise: Observation of a Dienolate Intermediate,” *Biochemistry* **2001**, *40*, 12412-12421.

[17] J. K. Hiltunen, A. M. Mursula, H. Rottensteiner, R. K. Wierenga, A. J. Kastaniotis, A. Gurvitz, “The Biochemistry of Peroxisomal *β*-Oxidation in the Yeast *Saccharomyces cerevisiae*,” *FEMS. Microbiol. Rev.* **2003**, *27*, 35-64.

[18] A. M. Mursula, D. M. F. van Aalten, J. K. Hiltunen, R. K. Wierenga, “The Crystal Structure of Delta(3)-Delta(2)-Enoyl-CoA Isomerase,” *J. Mol. Biol.* **2001**, *309*, 845-853.

[19] P. Kasaragod, W. Schmitz, J. K. Hiltunen, R. K. Wierenga, “The Isomerase and Hydratase Reaction Mechanism of the Crotonase Active Site of the Multifunctional Enzyme (type-1), as Deduced from Structures of Complexes with 3*S*-Hydroxy-Acyl-CoA,” *FEBS J.* **2013**, *280*, 3160-3175.

[20] Y. M. Qin, A. M. Haapalainen, D. Conry, D. A. Cuebas, J. K. Hiltunen, D. K. Novikov, “Recombinant 2-Enoyl-CoA Hydratase Derived from Rat Peroxisomal Multifunctional Enzyme 2: Role of the Hydratase Reaction in Bile Acid Synthesis,” *Biochem. J.* **1997**, *328*, 377-382.

[21] T. Veiga, A. K. Gombert, N. Landes, et al., “Metabolic Engineering of *β*-Oxidation in *Penicillium chrysogenum* for Improved Semi-Synthetic Cephalosporin Biosynthesis,” *Metab. Eng.* **2012**, *14*, 437-448.

[22] J. R. Glover, D. W. Andrews, S. Subramani, R. A. Rachubinski, “Mutagenesis of the Amino Targeting Signal of *Saccharomyces cerevisiae* 3-Ketoacyl-CoA Thiolase Reveals Conserved Amino Acids Required for Import into Peroxisomes *in vivo*,” *J. Biol. Chem.* **1994**, *269*, 7558-7563.

[23] S. Tada, K. Gomi, K. Kitamoto, C. Kumagai, G. Tamura, S. Hara, “Identification of the Promoter Region of the Taka-Amylase A Gene Required for Atarch Induction,” *Agr. Biol. Chem.* **1991**, *55*, 1939-1941.

[24] A. Watanabe, Y. Ono, I. Fujii, U. Sankawa, M. E. Mayorga, W. E. Timberlake, Y. Ebizuka, “Product Identification of Polyketide Synthase Coded by *Aspergillus nidulans* *wA* Gene,” *Tetrahedron Lett.* **1998**, *39*, 7733-7736.
